# Synthesis and Electrochemistry of Copper(I) Complexes with Weakly Basic, Fluorinated, and Multicyclic Arenes

**DOI:** 10.1002/chem.202501134

**Published:** 2025-04-27

**Authors:** Julie Willrett, Malte Sellin, Max Lapersonne, Matthis Seiler, Ingo Krossing

**Affiliations:** ^1^ Institut für Anorganische und Analytische Chemie und Freiburger Materialforschungszentrum (FMF) Albert‐Ludwigs‐Universität Freiburg Albertstr. 21 79104 Freiburg Germany

**Keywords:** arene ligands, copper, cyclic voltammetry, structure elucidation, weakly coordinating anions

## Abstract

The dinitrogen complex [(N_2_)Cu{Al(OR^F^)_4_}] (R^F^ = C(CF_3_)_3_) acts as the precursor for the synthesis of homoleptic, weakly bound Cu(I)‐arene complexes with benzene, fluorinated, and multicyclic arenes. Upon dissolution of [(N_2_)Cu{Al(OR^F^)_4_}] in benzene or the fluorinated arenes xFB (x = number of fluorine atoms, x = 1−4), the complexes [Cu(C_6_H_6_)_2−3_]^+^[Al(OR^F^)_4_]^−^, [Cu(xFB)_2−3_]^+^[Al(OR^F^)_4_]^−^ (x = 1−3), or the ion pair [(xFB)Cu{Al(OR^F^)_4_}] (x = 4) form. Both the benzene and fluorobenzene complexes are also available in larger scales by oxidation of elemental copper in the respective solvents using [NO]^+^[Al(OR^F^)_4_]^−^ as an oxidant. A higher degree of fluorination of the arene ligand leads to a weaker coordination of the Cu(I) atom and thus to higher Cu^+^/Cu redox potentials in solution that reach an unprecedentedly high value of +1.5 V (!) versus the ferrocenium/ferrocene couple in pentafluorobenzene. The versatile applicability of [(N_2_)Cu{Al(OR^F^)_4_}] as a “naked” Cu(I) source is demonstrated by complexation reactions with the multicyclic arenes anthracene and hexaphenylbenzene that yielded multiply charged cations. All complexes presented in this work were characterized by their single‐crystal X‐ray diffraction structures.

## Graphical abstract

**Weak Coordination, High Potentials**: Making use of the weakly coordinated copper(I)‐dinitrogen complex [(N_2_)Cu{Al(OR^F^)_4_}] (R^F^ = C(CF_3_)_3_) as a precursor, homoleptic Cu(I)‐arene complexes with weakly basic (fluorinated) arene ligands were synthesized. The higher the degree of fluorination of the arenes, the weaker are their interaction with Cu(I) and the higher are their Cu^+^/Cu redox potentials reaching +1.5 V versus Fc^+^/Fc in pentafluorobenzene.

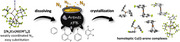

## 1 Introduction

Homoleptic copper salts with arene ligands are still scarce, although several novel copper salts paired with weakly coordinating anions (WCAs) and featuring weakly basic ligands such as white phosphorus,^[^ ^1^ ^]^ ethylene,^[^ ^1^ ^]^ carbon monoxide,^[^ ^2^ ^]^ and/or dichloromethane (DCM)^[^ ^3^ ^]^ were prepared over the last years. This is mainly due to the lack of suitable and accessible Cu(I)‐WCA precursors. Hence, already in the 1960s, the Cu(I)‐benzene complex [Cu(C_6_H_6_)(AlCl_4_)] has been synthesized by reaction of CuCl with AlCl_3_ in benzene.^[^ ^4^ ^]^ Yet, formation of a homoleptic benzene complex is inhibited by a strong coordination of the [AlCl_4_]^−^ anion, highlighting the need for good WCAs. And although “naked” Cu(I) salts of the better WCAs [EF_6_]^−^ (E = As, Sb) are available^[^ ^5^ ^]^ their syntheses involve the handling of AsF_5_ or anhydrous HF, rendering them inaccessible for many preparative chemists.

Using the widely applied fluorinated alkoxyaluminate WCA from our group,^[^ ^6^ ^]^ the first homoleptic Cu(I)arene complex was prepared in 2009 through a metathesis reaction of copper iodide CuI with silver fluoride AgF in the presence of the lithium salt Li^+^[Al(OR^F^)_4_]^−^ (R^F^ = C(CF_3_)_3_) in 1,2‐difluorobenzene (2FB) as solvent, yielding [Cu(2FB)_2_]^+^[Al(OR^F^)_4_]^−^.^[^ ^7^ ^]^ A simpler preparation of [Cu(2FB)_2_]^+^[Al(OR^F^)_4_]^−^ proceeds by oxidizing elemental copper powder with [NO]^+^[Al(OR^F^)_4_]^−^ in 2FB as solvent.^[^ ^8^ ^]^ Using [Cu(2FB)_2_]^+^[Al(OR^F^)_4_]^−^ as a starting material, exotic Cu(I) complexes such as [Cu(S_12_)(S_8_)]^+ [^ ^7^ ^]^ and [Cu_2_Se_19_]^2+ [^ ^9^ ^]^ could be synthesized. Furthermore, [Cu(2FB)_2_]^+^[Al(OR^F^)_4_]^−^ was employed as a starting point for a model system to elucidate the mechanism of the monooxygenation of phenols catalyzed by the copper‐containing enzyme tyrosinase.^[8–10]^ Similar copper‐based oxidases and oxygenases are widespread in nature,^[^ ^11^ ^]^ making molecular copper complexes interesting model compounds for bioinorganic chemistry.

Other known homoleptic Cu(I) arene complexes are limited to the *η* ^2^‐coordinated Cu(I)‐mesitylene complex [Cu(mes)_2_]^+^[PF_6_]^−^, prepared by reaction of copper with [NO]^+^[PF_6_]^−^ in mesitylene^[^ ^12^ ^]^ and the analogously prepared *η* ^2^‐coordinated [Cu(hmb)_2_]^+^[PF_6_]^−^ (hmb = hexamethylbenzene). Addition of PPh_3_ to the latter led to substitution of one hmb‐ligand to form the first unsupported *η* ^6^‐interaction of copper with an arene in the half‐sandwich complex [(hmb)Cu(PPh_3_)]^+^[PF_6_]^−^.^[^ ^13^ ^]^

Yet, the general access to solvent‐free Cu(I)‐WCA salts or synthons thereof, that would offer access to a variety of novel and useful copper complexes, is complicated. Only very strongly deelectronating agents such as the perfluoronaphthalene radical cation [C_10_F_8_]^+∙^ with a formal potential of +2.0 V versus ferrocenium/ferrocene (Fc^+^/Fc) and paired with the least coordinating WCA [F{Al(OR^F^)_3_}_2_]^−^, are able to oxidize copper powder in the polar, but almost noncoordinating solvents^[^ ^14^ ^]^ 1,2,3,4‐tetrafluorobenzene (4FB) or pentafluorobenzene (5FB) and yield the solvent‐free contact ion pair [Cu(F{Al(OR^F^)_3_}_2_)].^[^ ^15^ ^]^ The same reaction using the more accessible WCA [Al(OR^F^)_4_]^−^ is impossible due to the inferior compatibility of [C_10_F_8_]^+∙^ with this WCA.^[^ ^15^ ^]^ Attempts to oxidize copper powder with Ag^+^ salts as oxidants inevitably resulted in mixtures of Cu^+^ and Ag^+^ salts.^[^ ^16^, ^17^ ^]^ Finally, the repeated treatment of Ag^+^[Al(OR^F^)_4_]^−^ with excess CuI in consecutive “cascade” metathesis reactions under N_2_ atmosphere and using *iso*‐perfluorohexane as a solvent yields the silver‐free dinitrogen‐complex [(N_2_)Cu{Al(OR^F^)_4_}]. This synthesis is very reproducible, also in gram‐scale quantities.^[^ ^17^ ^]^ In this compound, the N_2_ ligand is very weakly bound and easy to substitute,^[^ ^17^ ^]^ making [(N_2_)Cu{Al(OR^F^)_4_}] the ideal synthon of a solvent‐free Cu(I)‐WCA salt.

To test its characteristic as a Cu(I)‐synthon, [(N_2_)Cu{Al(OR^F^)_4_}] was used in this work for the synthesis and structural investigation of weakly coordinated Cu(I)‐arene complexes, including parent benzene, as well as complexes with multicyclic arenes. To study the Cu(I)‐arene interactions and to investigate their behavior in solution, cyclovoltammetric (CV) measurements were conducted and rationalized with the help of quantum chemical calculations. Very high to extreme formal potentials^[^ ^18^ ^]^ *E*°’ of the redox pair Cu^+^/Cu versus Fc^+^/Fc between 0.7 and 1.5 V were determined in and assigned to five solvents, augmenting the currently largely underrepresented Cu^+^/Cu CV‐data in the literature.

## 2 Results and Discussion

### 2.1 Syntheses of the Cu(I)‐Arene Complexes and NMR‐Spectroscopy

When [(N_2_)Cu{Al(OR^F^)_4_}] is dissolved in benzene or fluorinated benzene derivatives xFB (x denotes the number of adjacent fluorine atoms: 1FB = fluorobenzene, 2FB = 1,2‐difluorobenzene, 3FB = 1,2,3‐trifluorobenzene etc.), the Cu(I)‐solvent complexes [Cu(C_6_H_6_)_2−3_]^+^[Al(OR^F^)_4_]^−^ and [Cu(xFB)_2−3_]^+^[Al(OR^F^)_4_]^−^ (x = 1−3, Equation 1a) or the ion pair [(xFB)Cu{Al(OR^F^)_4_}]^[^ ^17^ ^]^ (x = 4, Equation 1b) can be obtained. In 5FB and 6FB, [(N_2_)Cu{Al(OR^F^)_4_}] is soluble without decomposition and without formation of the solvent complexes.


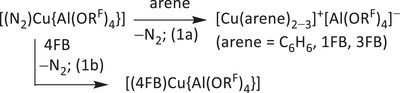


While the use of the dinitrogen complex [(N_2_)Cu{Al(OR^F^)_4_}] is crucial for the synthesis of both the 3FB‐ and the 4FB‐complex, the Cu(I) complexes of the more basic arenes benzene and 1FB can also be obtained either from literature‐known [Cu(2FB)_2_]^+^[Al(OR^F^)_4_]^−^ by ligand metathesis (Equation 2) or by oxidation of copper powder with [NO]^+^[Al(OR^F^)_4_]^−^ with the respective arene as solvent (Equation 3), similar to the published synthesis of the 2FB‐complex.^[^ ^8^ ^]^ These methods are especially useful for upscaled syntheses, as the precursors are more easily available than the dinitrogen complex [(N_2_)Cu{Al(OR^F^)_4_}]. Yet, the preparation of Cu(I)‐xFB complexes from [(N_2_)Cu{Al(OR^F^)_4_}] is particularly neat and reliable with only N_2_ as byproduct, which makes it the method of choice for their use in electrochemical measurements (*vide infra*).


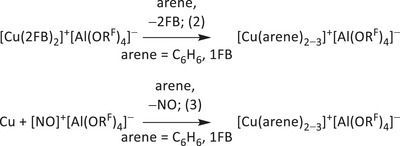


The direct oxidative route in Equation 3 works reliably only for the benzene and fluorobenzene complexes. For 1FB, good yields of roughly 70% are obtained, while for benzene, 2FB has to be added as an additional solvent to increase the solubility of both starting material and product, and thus to increase the yield compared to the reaction in pure benzene. The successful synthesis of the *tris*‐benzene complex [Cu(C_6_H_6_)_3_]^+^[Al(OR^F^)_4_]^−^ **1** and the *tris*‐1FB complex [Cu(1FB)_3_]^+^[Al(OR^F^)_4_]^−^ **3** was confirmed by NMR spectroscopy (Supporting Information, Section 3). For **1**, a shift of the benzene signal in the ^1^H NMR spectrum in CD_2_Cl_2_ to 7.47 ppm is observed compared to free benzene at 7.35 ppm.^[^ ^19^ ^]^ For **3**, a similar shift to a lower field is observed: **3** features two multiplets at 7.35−7.51 ppm and 7.08−7.25 ppm, which correspond to the multiplets at 7.03−7.12 ppm and 6.82−6.94 ppm in free 1FB. A similar shift can be observed in the ^19^F NMR spectrum: from −113.24 ppm in free 1FB to −112.02 ppm for **3**. Quantitative NMR spectroscopy confirmed that both **1** and **3** do not lose an arene ligand to form the *bis*‐benzene complex [Cu(C_6_H_6_)_2_]^+^[Al(OR^F^)_4_]^−^ **2** and the *bis*‐1FB complex [Cu(1FB)_2_]^+^[Al(OR^F^)_4_]^−^ **4**, when dried in vacuo during isolation of the products (Supporting Information, Section 3).

### 2.2 Structures and Energetics of the Cu(I)‐xFB Complexes from [(N_2_)Cu{Al(OR^F^)_4_}]

Depending on the reaction and crystallization conditions, crystals of either the *tris*‐benzene complex [Cu(C_6_H_6_)_3_]^+^[Al(OR^F^)_4_]^−^ **1** or less stable crystals of the *bis*‐benzene complex [Cu(C_6_H_6_)_2_]^+^[Al(OR^F^)_4_]^−^ **2** can be obtained from [(N_2_)Cu{Al(OR^F^)_4_}]: Addition of only two equivalents of C_6_H_6_ and layering with *n*‐heptane favors the formation of **2**. However, when C_6_H_6_ is used in excess as solvent and no antisolvent is added, exclusively **1** crystallizes. Similarly, both the copper *tris*‐fluorobenzene complex [Cu(1FB)_3_]^+^[Al(OR^F^)_4_]^−^ **3** and the less stable *bis*‐fluorobenzene complex [Cu(1FB)_2_]^+^[Al(OR^F^)_4_]^−^ **4** are accessible depending on which crystallization method is chosen. In addition, **2** and **4** can be selectively obtained from **1** and **3** by (re‐)crystallization using the layering method with hydrocarbons. From 3FB solution, the salt structure of [Cu(3FB)_2_]^+^[Al(OR^F^)_4_]^−^ **5** crystallizes. Yet, in very weakly coordinating 4FB, the contact ion pair [(4FB)Cu{Al(OR^F^)_4_}] **6** forms, in which the anion serves as an auxiliary ligand. Note that, while the *tris*‐arene complexes only slowly decompose when brought to open air, the *bis*‐arene complexes quickly decompose, and the contact ion **6** requires low temperatures (−20 °C) for handling (Supporting Information, Section 3).

**Figure 1:**
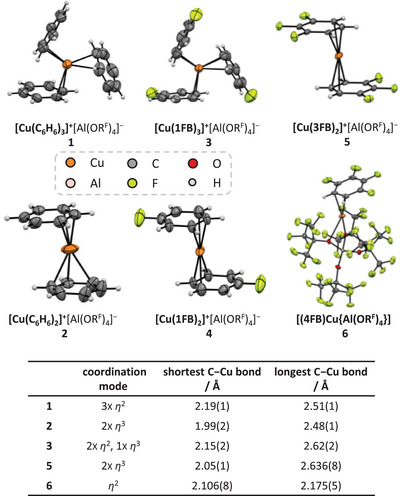
Molecular structures of the cationic moieties of [Cu(C_6_H_6_)_3_]^+^[Al(OR^F^)_4_]^−^ **1** (*Pna*2_1_, *R*_1_ = 7.8%, *wR*_2_ = 24.5%, measured at 200 K), [Cu(C_6_H_6_)_2_]^+^[Al(OR^F^)_4_]^−^ **2** (*P* $\bar{1}$, *R*_1_ = 9.0%, *wR*_2_ = 27.7%), [Cu(1FB)_3_]^+^[Al(OR^F^)_4_]^−^ **3** (*Pna*2_1_, *R*_1_ = 6.5%, *wR*_2_ = 18.2%, measured at 200 K), [Cu(1FB)_2_]^+^[Al(OR^F^)_4_]^−^ **4** (*P* $\bar{1}$, *R*_1_ = 8.0%, *wR*_2_ = 24.2%), [Cu(3FB)_2_]^+^[Al(OR^F^)_4_]^−^ **5** (*P*2_1_/*c*, *R*_1_ = 4.2%, *wR*_2_ = 9.8%) and crystal structure of the ion‐pair [(4FB)Cu{Al(OR^F^)_4_}] **6** (modification **6a**: *P*2_1_/*n*, *R*_1_ = 10.7%, *wR*_2_ = 26.5%, modification **6b**: *Cc*, *R*_1_ = 5.5%, *wR*_2_ = 13.5%). Disorders omitted for clarity (for complete structures see Supporting Information, Section 6). Thermal ellipsoids are shown at the 50% probability level. Selected bond lengths are given where appropriate (the crystallographic data for **4** do not allow for a discussion of coordinative motives or bond lengths, see Supporting Information, Section 6). For **6**, bond lengths for modification **6a** are given due to the better C─C bond precision (despite the overall higher *R*_1_ value for **6a**). Yet, the bond lengths in **6b** are identical inside the error margins to **6a**.

All the crystal structures determined by scXRD (single‐crystal X‐ray diffraction methods, Figure 1) feature multiple disorders of the arene ligands relative to the copper atom. In most of the cases, the disorders consist of a rotation of the arene ligand, which can occupy two distinct sites with occupancies ranging from 50/50 to 65/35 (Supporting Information, Section 4). This hints at a very flat potential energy surface for the rotation of the arene ligands and at low rotational energy barriers, which are often observed for metal arene complexes in the literature and that amount to only a few kJ mol^−1^.^[^ ^20^, ^21^ ^]^ Furthermore, a quantum‐chemical study of the binding of the coinage metals to benzene shows that the copper atom is very mobile in copper‐arene complexes, as different coordination modes (*η* ^1^, *η* ^2^, and *η* ^6^) differ only marginally in energy,^[^ ^21^ ^]^ which clearly should favor disorders in the crystal structure. Having said that, the *tris*‐arene complexes and the ion pair presented in this work show a preference for *η* ^2^‐coordination of the arene ligands, while the *bis*‐arene complexes feature exclusively *η* ^3^‐coordination (Figure 1).

To quantify the binding energy of the third arene‐ligand and to explain the fleeting stability of the *tris*‐arene complexes compared to the *bis*‐arene complexes, we conducted quantum chemical calculations at the RI‐r^2^SCAN‐3c(D4)/def2‐mTZVPP level collected in Table 1 and I. While for benzene the *bis*‐ and *tris*‐arene complexes are almost equal in energy, the *bis*‐arene complex becomes more and more favored with increasing degree of fluorination of the ligand. This agrees with the formation of the *bis*‐ and *tris*‐arene complexes with C_6_H_6_ and 1FB as a function of the quantity of available arene ligand and also solvent polarity. Hence, with these observations for C_6_H_6_ and 1FB the equilibrium constant K for this process must lie around 1 and with the low concentrations in the millimolar range used for crystallization, Δ_r_ *G°* should be a bit below 0 kJ mol^−1^, well in the calculated range of –0.5 (C_6_H_6_)/ –10 kJ mol^−1^ (1FB). Also, the experimentally exclusively observed *bis*‐arene complex structures with 2FB^[^ ^7^, ^8^ ^]^ and 3FB agree with the calculations that are favored in Δ_r_ *G°* by –20 (2FB) / –23 kJ mol^−1^ (3FB).

**Table 1:** Computed Gibbs free energies (Δ_r_ *G*°; RI‐r^2^SCAN‐3c(D4)/def2‐mTZVPP) of the formation of the *bis*‐arene complex starting from the *tris*‐arene complex for the ligands C_6_H_6_ and xFB with x = 1−3 (I), the isodesmic ligand exchange between cationic silver‐ and copper‐arene complexes (II) and of the isodesmic ligand exchange between neutral silver and copper ion‐pairs (III). Solvation Gibbs energies were computed with the CPCM‐model.^[^ ^a^ ^]^

	Reaction	x	*n*	Δ_r_ *G°* / kJ mol^−1^
I)	[Cu(xFB)*_n_*]^+^_(solv,xFB)_ → [Cu(xFB)*_n_*_−1_]^+^_(solv,xFB)_ + xFB_(solv,xFB)_	0 (C_6_H_6_)	3	−0.5
		1	3	−10.1
		2	3	−20.3
		3	3	−23.4
II)	[Ag(xFB)*_n_*]^+^_(solv,xFB)_ + Cu^+^_(solv,xFB)_ →	1	3	−43.6
	Ag^+^_(solv,xFB)_ + [Cu(xFB)*_n_*]^+^_(solv,xFB)_	2	2	−44.0
		3	2^[^ ^b^ ^]^	−40.9
III)	[(4FB)Ag{Al(OR^F^)_4_}] + [Cu{Al(OR^F^)_4_}] → [Ag{Al(OR^F^)_4_}] + [(4FB)Cu{Al(OR^F^)_4_}]	−	−	−5.2^[^ ^c^ ^]^

^[a]^ Solvation Gibbs energies computed for the corresponding solvent xFB using the CPCM‐model and at the RI‐r^2^SCAN‐3c(D4)/def2‐mTZVPP level of theory.

^[b]^ It is supposed that the Ag^+^ *bis*‐3FB complex is the species present in solution, while the published crystal structure of the ion‐pair [(3FB)Ag{Al(OR^F^)_4_}] prevails in the solid‐state.^[^ ^14^ ^]^

^[c]^ Energies computed in the gas phase due to long computational times for the large anion. As only neutral compounds are involved, solvation energies are comparatively small and similar for all compounds and thus negligible.

Note that the isodesmically calculated exchange energies of the silver‐arene‐complexes with the Cu^+^ ion described in Table 1 (entries II and III) are needed to rationalize the CV‐measured formal potentials in one of the next sections and are described and utilized there.

### 2.3 Formal Potentials of the Cu(I)‐xFB Complexes versus Ferrocene

**Figure 2:**
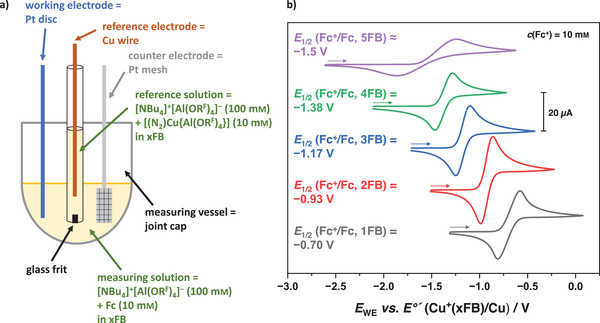
a) Schematic setup of the CV measurements against a Cu compartment electrode (in analogy to reference^[^ ^14^ ^]^). b) Formal potentials of the Cu^+^ cation in different solvents xFB (x = 1−5). First cycles of the CVs (*ν* = 100 mV s^−1^) of solutions of Fc (*c* = 10 mm) in the solvents xFB (x = 1−5) with the conducting salt [NBu_4_]^+^[Al(OR^F^)_4_]^−^ (*c* = 100 mm) corrected to the formal potential *E*°´ by −0.118 V (cf. reference^[^ ^14^ ^]^). For x = 1−4 the half‐wave potential is independent of the scan rate between 20 and 100 mV s^−1^. For 5FB see text and Supporting Information, Section 5.

To determine the Cu^+^ potentials in different solvents xFB (x = 1−5) and compare them to literature‐known potentials of the heavier analogue Ag^+^, which is widely used as a potent inorganic oxidant,^[^ ^14^, ^22^ ^]^ we used the same CV‐methodology recently published for the determination of Ag^+^ potentials (Supporting Information, Section 5 and reference^[^ ^14^ ^]^). For this purpose, we measured the Fc^+^/Fc potentials in xFB against a copper reference, consisting of a copper wire as electrode immersed in a solution of [(N_2_)Cu{Al(OR^F^)_4_}] in the respective solvent. The Cu^+^/Cu formal potentials were obtained by taking the negative Fc^+^/Fc formal potentials measured against the Cu^+^/Cu reference. In Figure 2a, the measurement setup is shown, and in Figure 2b, the first CV cycles of each measurement are shown at a scan rate of 100 mV s^−1^. In Table 2, the derived formal potentials of the Cu^+^ ions are listed and compared to the Ag^+^ potentials.

**Table 2:** Formal potentials of the Cu^+^ cation in different solvents xFB (x = 1−5) *E*°´(Cu^+^(xFB)/Cu) versus *E*°´((Fc^+^/Fc),xFB) in comparison with the published^[^ ^14^ ^]^ formal potentials of the Ag^+^ cation in the same solvents.^[^ ^a^ ^]^

	*E°´*(Cu^+^(xFB)/Cu) / V	*E°´*(Ag^+^(xFB)/Ag)^[^ ^14^ ^]^ / V	*E°´*(Ag^+^(xFB)/Ag) − *E°´*(Cu^+^(xFB)/Cu) / V
1FB	0.70	0.74	0.04
2FB	0.93	0.99	0.06
3FB	1.17	1.26	0.09
4FB	1.38	1.47	0.09
5FB	> 1.5^[^ ^b^ ^]^	1.50	−

^[a]^ Determined from the 1^st^ cycles of the CVs (*ν* = 100 mV s^−1^) of solutions of Fc (*c* = 10 mm) in the solvents xFB (x = 1−5) with the conducting salt [NBu_4_]^+^[Al(OR^F^)_4_]^−^ (*c* = 100 mm), a copper wire in a compartment as a reference electrode and corrected to the formal potential *E*°´ by −0.118 V (cf. reference^[^ ^14^ ^]^). For x = 1−4 the half‐wave potential is independent of the scan rate between 20 and 1000 mV s^−1^. See Supporting Information, Section 5 for details.

^[b]^ The Cu^+^ potential is not stable over the scan rates but increases with increasing scan rate in the weakly polar solvent 5FB. Still, the potential is higher than 1.5 V for all measured scan rates between 20 and 1000 mV s^−1^.

Similar to the silver potentials, the copper formal potentials *E°*’ increase considerably for increasing x from +0.70 V (1FB) to > +1.5 V (5FB). Note that only for x = 1−4 the half‐wave potentials are independent of the investigated scan rates (Supporting Information, Section 5; measured scan rates: 20, 50, 100, 200, 500, 1000 mV s^−1^). While the Cu^+^/Cu redox couple is scarcely discussed in the literature, the Cu^2+^/Cu^+^ couple has been thoroughly investigated. Despite the higher second ionization energy (*IE*) of copper, the Cu^2+^/Cu^+^ potentials of solvated copper‐coordination compounds are throughout considerably lower than our reported Cu^+^/Cu potentials,^[^ ^23^ ^]^ reaching low values down to −1.40 V versus Fc^+^/Fc reported for a homoleptic copper complex with redox‐active urea azine ligands.^[^ ^24^ ^]^ This calls for a rationalization of these unusually high Cu^+^/Cu formal potentials measured here. Hence, we compare them to those of the respective silver salts in the like solvents that were thoroughly investigated and are reliable.^[^ ^14^ ^]^ Interestingly, the Cu^+^ potential in xFB with x = 1−4 is by 0.04 to 0.09 V lower than the corresponding Ag^+^ potential. In 5FB, the Cu^+^ formal potential cannot be reliably determined as the half‐wave potential increases with increasing scan rates and does not reach a stable value (Supporting Information, Section 5). This is likely caused by the coordination of N_2_ to the copper atom, which persists in 5FB solution and in contrast to all the other examined solvents. This complexation formally changes the investigated redox system from Cu^+^/Cu to [Cu(N_2_)]^+^/Cu. Nevertheless, it can be clearly deduced that this mixed Cu^+^ potential reaches (and for the examined scan rates even surpasses) the Ag^+^ potential of 1.50 V versus Fc^+^/Fc. All of this agrees with the observation that in 5FB the interaction energies of both, the Cu^+^ and the Ag^+^ ion with the solvent become very small. Hence, the (mixed) potential in 5FB complies with the by +0.15 eV higher ionization energy of copper (*IE*_Cu_ = 7.7264 eV)^[^ ^25^ ^]^ compared to silver (*IE*_Ag_ = 7.5762 eV).^[^ ^25^ ^]^ By contrast, in the more basic arenes xFB with x = 1−4, the smaller and more Lewis‐acidic Cu^+^ ion apparently interacts more strongly with the solvents than Ag^+^, which may account for the lower potentials in these solvents.

### 2.4 Comparison to Calculated Energetics

To prove this point, quantum chemical calculations were conducted to quantify the strengths of Cu^+^‐arene interactions in comparison to the Ag^+^‐arene interactions (Table 1, entries II and III). Hence, the Gibbs free energies of the isodesmic ligand exchange between Cu^+^‐ and Ag^+^‐arene complexes were computed at the RI‐r^2^SCAN‐3c(D4)/def2‐mTZVPP level of density functional theory (DFT). For 1FB, the *tris*‐arene complexes were regarded, for 2FB and 3FB the *bis*‐arene complexes and for 4FB the ion‐pairs with one coordinated 4FB arene. In the homoleptic salt structures, the Cu^+^‐arene interactions are indeed stronger than the Ag^+^‐arene interactions by approximately 40 kJ mol^−1^. With Δ*G* = –zFΔ*E*, this would lower their potential by about 0.4 V. In the ion pairs, the same effect is observed, however, less pronounced with a better Cu^+^‐arene interaction of 5 kJ mol^−1^ or 0.05 V. Thus, from the observed formal potential changes ‐ with lower Cu^+^‐ than Ag^+^‐potentials in xFB for x = 1−4 ‐ one learns that the by 0.15 V higher gas phase *IE*_Cu_ is overwritten by the 0.4 V higher xFB‐complexation energy of the Cu^+^ ion. If fully accessible, this would lead to about 0.25 V lower Cu^+^‐ versus Ag^+^‐ potentials in xFB solution. The experimental differences of 0.04 to 0.09 V in Table 2 thus suggest leveling effects on the potential, for example by ion‐pairing. Yet, the levelling effects get smaller for the least interacting solvent 5FB.

### 2.5 Using the Weakly Coordinated Copper(I) Salts: Complexation Reactions with Multicyclic Arenes

The easy availability of Ag^+^[WCA]^−^ salts has led to a wide supramolecular chemistry, featuring various silver‐arene complexes including diverse multicyclic arenes, e.g., acenes,^[^ ^26^, ^27^ ^]^ coronene^[^ ^28^ ^]^ or even helicenes.^[^ ^29^ ^]^ The structures of homoleptic Cu(I) complexes with multicyclic arenes, on the other hand, are very scarce. To show the versatility of the herein presented copper(I) precursors, we exemplarily isolated some adducts between copper(I) and multicyclic arenes.*

**Figure 3:**
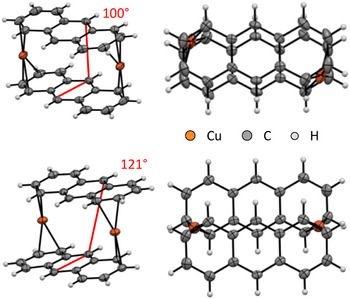
Molecular structures of the two crystallographically independent [Cu_2_(anthracene)_2_]^2+^ moieties in [Cu_2_(anthracene)_2_]^2+^([Al(OR^F^)_4_]^−^)_2_∙(6FB)_1.5_ **7**∙(6FB)_1.5_ (*P* $\bar{1}$, *R*_1_ = 3.6%, *wR*_2_ = 8.6%). Diagonal views on the complex on the left and top views on the complex on the right. Thermal ellipsoids are shown at the 50% probability level.

First, we reacted [Cu(2FB)_2_]^+^[Al(OR^F^)_4_]^−^ with anthracene, which resulted in the 2:2 complex [Cu_2_(anthracene)_2_]^2+^([Al(OR^F^)_4_]^−^)_2_ **7**. The overall structure is analogous to the recently prepared silver complex,^[^ ^26^ ^]^ comprising two parallelly aligned anthracene moieties with a metal ion at each end between the anthracene ligands, forming a characteristic baguette‐like sandwich structure. While the [Ag_2_(anthracene)_2_]^2+^ cation has a nearly rectangular arrangement of the anthracene ligands, the anthracene rings are significantly slipped to the side in the copper analogue. The structure of [Cu_2_(anthracene)_2_]^2+^([Al(OR^F^)_4_]^−^)_2_∙(6FB)_1.5_ features two crystallographically independent [Cu_2_(anthracene)_2_]^2+^ moieties, of which one features less slipped and the other features more‐strongly slipped anthracene ligands (Figure 3). This different sideways translation of the anthracene ligands results in different coordination environments: while the copper atoms in the strongly slipped [Cu_2_(anthracene)_2_]^2+^ complex are exclusively *η* ^2^,*η* ^2^‐coordinated by the anthracene ligands, the copper atoms in the less‐slipped [Cu_2_(anthracene)_2_]^2+^ are *η* ^3^,*η* ^2^‐coordinated by the anthracene ligands.

**Figure 4:**
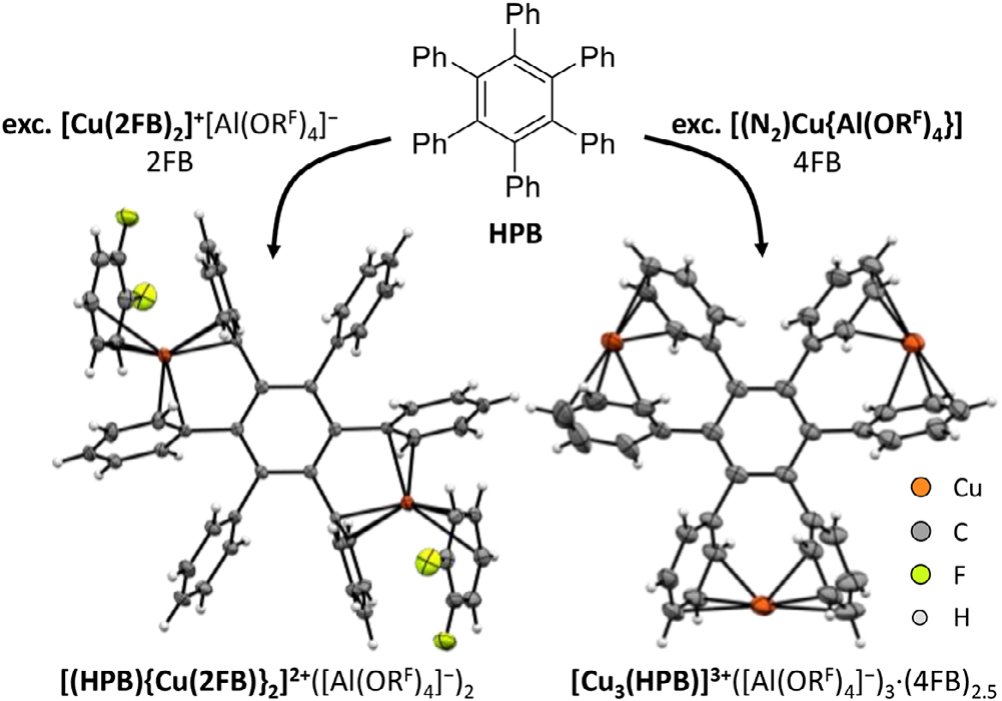
Molecular structures of the copper arene cations in [(HPB){Cu(2FB)}_2_]^2+^([Al(OR^F^)_4_]^−^)_2_ **8** (*P* $\bar{1}$, *R*_1_ = 4.8%, *wR*_2_ = 11.9%) and [Cu_3_(HPB)]^3+^([Al(OR^F^)_4_]^−^)_3_ **9** (*P* $\bar{1}$, *R*_1_ = 6.5%, *wR*_2_ = 17.6%) resulting from the reactions of HPB with [Cu(2FB)_2_]^+^[Al(OR^F^)_4_]^−^ and [(N_2_)Cu{Al(OR^F^}_4_]. Thermal ellipsoids are shown at the 50% probability level.

To compare the properties of the two [Cu(2FB)_2_]^+^[Al(OR^F^)_4_]^−^ and [(N_2_)Cu{Al(OR^F^)}_4_] reagents for the application as Cu^+^ transfer reagents, we reacted an excess of each of these salts with hexaphenylbenzene (HPB). While the reaction with [Cu(2FB)_2_]^+^[Al(OR^F^)_4_]^−^ in 2FB only affords the 2:1 adduct [(HPB){Cu(2FB)}_2_]^2+^([Al(OR^F^)_4_]^−^)_2_ **8**, the reaction of HPB with an excess of [(N_2_)Cu{Al(OR^F^}_4_] in 4FB yields the solvent‐free 3:1 adduct [Cu_3_(HPB)]^3+^([Al(OR^F^)_4_]^−^)_3_ **9** (Figure 4). This indicates that the stronger solvation of the copper(I) cation in 2FB prevents the formation of a trication and thereby demonstrates the superiority of [(N_2_)Cu{Al(OR^F^}_4_] as a source for a “naked” Cu^+^.

## 3 Conclusion

In this work, we presented the dinitrogen complex [(N_2_)Cu{Al(OR^F^)}_4_] as an accessible and very useful synthon to a solvent‐free Cu(I)‐WCA salt. It allows access to a wide range of novel, homoleptic Cu(I)‐arene complexes. Determination of the Cu^+^/Cu formal potentials versus Fc^+^/Fc revealed, particularly for the highly fluorinated systems, high to extreme potentials *E*°’ between 0.7 and > +1.5 V versus Fc^+^/Fc in xFB. These unusual values surpass those of the majority of published Cu^2+^/Cu^+^ systems and highlight the weak solvation of the Cu^+^ ion in these solvents and the weak Cu‐arene interactions. We anticipate widespread use of these versatile Cu(I) starting materials and the use of their formal potentials as a reference.

## Supporting Information

The electronic supporting information contains the general synthetic methods and characterization techniques used for this work together with the experimental procedures. Additional figures and tables such as NMR, IR and Raman spectra are presented, as well as the crystallographic data of the isolated salts and quantum chemical calculations. The authors have cited additional references within the supporting information.^[^ ^30^ ^]^

## Author Contributions

IK supervised and conceptionally devised the project. JW performed the DFT calculations and most of the synthetic work together with ML. The cyclic voltammetry was performed by JW and MSellin. MSellin performed the complexation reactions with multicyclic arenes together with MSeiler. The manuscript was written by JW together with IK and MSellin.

## Conflict of Interests

The authors declare no conflict of interest.

## Supporting information



Supporting Information

## Data Availability

Deposition Numbers 2393904 (for [Cu(C_6_H_6_)_3_][Al(OR^F^)_4_]), 2393903 (for [Cu(C_6_H_6_)_2_][Al(OR^F^)_4_]), 2393905 (for [Cu(1FB)_3_][Al(OR^F^)_4_]), 2407672 (for [Cu(1FB)_2_][Al(OR^F^)_4_], description 1), 2407673 (for [Cu(1FB)_2_][Al(OR^F^)_4_], description 2), 2393906 (for [Cu(3FB)_2_][Al(OR^F^)_4_]), 2407619 (for [(4FB)Cu{Al(OR^F^)_4_}], modification 1), 2407671 (for [(4FB)Cu{Al(OR^F^)_4_}], modification 2), 2407626 (for [Cu_2_(anthracene)_2_]^2+^([Al(OR^F^)_4_]^−^)_2_∙(6FB)_1.5_), 2407624 (for [(HPB){Cu(2FB)}_2_]^2+^([Al(OR^F^)_4_]^−^)_2_), 2407625 (for [Cu_3_(HPB)]^3+^([Al(OR^F^)_4_]^−^)_3_∙(4FB)_2.5_) contain the supplementary crystallographic data for this paper. These data are provided free of charge by the joint Cambridge Crystallographic Data Centre and Fachinformationszentrum Karlsruhe “http://www.ccdc.cam.ac.uk/structures” Access Structures service.

## Footnotes

* Note that these are predominantly structural investigations of the single crystals obtained without further investigations of the bulk materials apart from bulk analysis and mode assignment by IR and Raman spectroscopy (Supporting Information, Section 3.10).
