# Supplementary material for: Synthesis and Electrochemistry of Copper(I) Complexes with Weakly Basic, Fluorinated, and Multicyclic Arenes
Source: Chemistry. 2025 Apr 27;31(29):e202501134. doi: 10.1002/chem.202501134 (PMC12099181; doi:10.1002/chem.202501134)
Supplement: Supplementary file 1 — Supporting Information [file CHEM-31-e202501134-s001.pdf]

Table of contents

1. Abbreviations 1
2. Material and Methods 2
3. Experimental Procedures, NMR and Vibrational Spectra 4
   3.1. $[Cu(C_6H_6)_3]^+[Al(OR^F)_4]^-$ 1 4
   3.2. $[Cu(C_6H_6)_2]^+[Al(OR^F)_4]^-$ 2 7
   3.3. $[Cu(1FB)_3]^+[Al(OR^F)_4]^-$ 3 7
   3.4. $[Cu(1FB)_2]^+[Al(OR^F)_4]^-$ 4 11
   3.5. $[Cu(3FB)_2]^+[Al(OR^F)_4]^-$ 5 11
   3.6. $[(4FB)Cu\{Al(OR^F)_4\}]$ 6 12
   3.7. $[Cu_2(anthracene)_2]^{2+}([Al(OR^F)_4]^-)_2{\cdot}(6FB)_{1.5}$ $7{\cdot}(6FB)_{1.5}$ 12
   3.8. $[(Hexaphenylbenzene)\{Cu(2FB)\}_2]^{2+}([Al(OR^F)_4]^-)_2$ 8 13
   3.9. $[Cu_3(hexaphenylbenzene)]^{3+}([Al(OR^F)_4]^-)_3{\cdot}(4FB)_{2.5}$ $9{\cdot}(4FB)_{2.5}$ 14
   3.10. Assignment of Vibrational Bands 16
   3.11. Oxidative Syntheses of Cu(I)-Arene Complexes 18
   3.12. Determination of the Number of Arene Ligands in 1 and 3 in Solution 19
4. Single-Crystal XRD Data 21
5. Cyclic voltammetry 36
6. Computational details 44
7. References 59

1. Abbreviations

1FB	= fluorobenzene	Et	= ethyl
2FB	= 1,2-difluorobenzene	Fc^+/Fc	= ferrocenium/ferrocene
3FB	= 1,2,3-trifluorobenzene	FTIR	= Fourier-transform infrared spectroscopy
4FB	= 1,2,3,4-tetrafluorobenzene	FWHM	= full width at half maximum
5FB	= pentafluorobenzene	NMR	= nuclear magnetic resonance
$[Al(OR^F)_4]^-$	= $[Al\{OC(CF_3)_3\}_4]^-$	RT	= room temperature
Bu	= butyl	scXRD	= single-crystal X-ray diffractometry
CV	= cyclic voltammetry	TMS	= tetramethylsilane
DFT	= density functional theory		

2. Material and Methods

General procedure

All manipulations were carried out under a dinitrogen or argon atmosphere and exclusion of air and moisture using MBraun gloveboxes filled with dinitrogen or argon (O_2/H_2O < 1 ppm) and standard Schlenk techniques. All reactions were carried out in Schlenk tubes equipped with grease-free PTFE or glass valves. All glassware was dried over-night in an oven at 150 °C and was thoroughly flame-dried prior to usage. The solvents benzene (Sigma Aldrich), fluorobenzene (1FB, Sigma Aldrich), 1,2-difluorobenzene (2FB, Fluorochem), 1,2,3-trifluorobenzene (3FB, Apollo Scientific), pentafluorobenzene (5FB, ABCR), hexafluorobenzene (6FB, P & M − Invest), CD_2Cl_2 (99.6 %, Deutero) and *n*-heptane (Carl Roth) were dried over CaH_2 and distilled onto and stored over activated 3 Å molecular sieves. *N*-pentane (VWR) was dried using a Grubbs apparatus and stored over 3 Å molecular sieves. 1,2,3,4-Tetrafluorobenzene (4FB, Apollo Scientific) was dried over CaH_2, distilled onto $Ag^+[Al(OR^F)_4]^-$ and condensed onto and stored over activated 3 Å molecular sieves. Oxygen was removed from all the solvent by either purging with argon or performing multiple freeze-pump-thaw cycles. Copper powder (chemPur, 99.9 %, >10 micron) was reduced under a stream of hydrogen for 2 h at 300 °C prior to use. Ferrocene was used from laboratory stock and was sublimed at 100 °C and 10^{-3} mbar. Hexaphenylbenzene (Sigma Aldrich) and anthracene (Sigma Aldrich) were used as received. $[NO]^+[Al(OR^F)_4]^-$,[1] $[(N_2)Cu\{Al(OR^F)_4\}]$,[2] $[Cu(2FB)_2]^+[Al(OR^F)_4]^-$[3] and $[NBu_4]^+[Al(OR^F)_4]^-$[4] were prepared using literature-known syntheses.

Vibrational Spectroscopy

ATR FTIR spectra were recorded inside a glovebox with a Bruker ALPHA spectrometer equipped with a QuickSnap Platinum ATR sampling module and a ZnSe crystal or a diamond. Spectra were measured at RT with 32 scans and a resolution of 2 cm^{-1} in the range of 4000-550 cm^{-1}.
Raman measurements were carried out at on a Vertex 70 IR spectrometer with installed RAM II Raman module (1064 nm exciting line of a Nd-YAG laser) and liquid nitrogen cooled Ge detector. Samples were flame-sealed in glass Pasteur pipettes. Spectra were recorded at RT in a range from 4000-80 cm^{-1} and a resolution of 4 cm^{-1} with a laser power of 25 mW and 5,000 scans.
Spectroscopic data were processed with the Bruker OPUS 7.5 software and baseline corrections were carried out with five iterations. The signal intensity was normalized and the relative intensities were reported as followed with regard to the most intensive signal: ≥ 0.7 = very strong (vs), ≥ 0.6 strong (s), ≥ 0.5 = medium strong (ms), ≥ 0.4 = medium (m), ≥ 0.3 = medium weak (mw), ≥ 0.2 = weak (w), < 0.2 = very weak (vw). The graphical representations were created with ORIGINPRO 2021.

NMR Spectroscopy

NMR spectra were recorded at RT on a Bruker Avance III HD 300 MHz spectrometer. The samples were dissolved in the NMR solvent (0.7 mL) in a 5 mm thick-walled NMR tube with J. Young PTFE valve under inert conditions (N_2 atmosphere). The spectra were calibrated by using the ^1H signal of the solvent according to the literature. The field corrections of other nuclei were adjusted accordingly. Spectroscopic data was analysed and plotted using Bruker TopSpin 4.1.3 software. ^1H NMR spectra are referenced against TMS, ^{19}F NMR spectra against $CFCl_3$, ^{27}Al NMR spectra against an aqueous solution of $Al(NO_3)_3$ (1.1 mM) and ^{63}Cu NMR spectra against $[Cu(CH_3CN)_4][ClO_4]$ + 10% C_6D_6. The broad signal at around 60 ppm in the ^{27}Al spectra stems from the probe head and the broad signal at around 1997 ppm in the ^{63}Cu spectra stems from the spectrometer.

Cyclic voltammetry

The cyclic voltammograms were recorded in an argon filled glovebox (O_2/H_2O < 0.1 ppm). A three-electrode arrangement was used with a 1 mm diameter platinum disc working electrode, a platinum mesh as counter electrode and a copper wire in a compartment as a reference. For a more detailed description of the measurement setup, see section 7. A VMP3 potentiostat (BIO-LOGICSCIENCE INSTRUMENTS) was used for the measurements, controlled via PC using the software EC-LAB (V11.21). The graphical representations were created with ORIGINPRO 2021.

Single Crystal X-Ray Diffraction

Single crystal X-ray diffraction data was acquired using a D8 Venture Photon III HPAD (Bruker) detector diffractometer. Crystal selection was performed at RT under PFPE oil JC 1800 (Sunoit Performance Material Science). Crystals were mounted on 0.1 to 0.2 mm diameter CryoLoops and were shock-cooled using an Oxford Cryostream 800 low temperature device.[5] Data were collected at 100(2) K or 200(2) K using monochromatic MoK_α radiation (λ = 0.71073 Å). Crystallographic data were integrated with SAINT (version 8.40B) and a multi-scan absorption correction using SADABS or TWINABS was performed.[6] Structures were solved by direct methods with SHELXT[7] and refined by full-matrix least-squares methods against F^2 by SHELXL-2019/3[8] using the GUI software ShelXle.[9] Refinement of disordered moieties was done using bond lengths restraints and displacement parameter restraints and was performed using the program DSR.[10] Finalization of the gathered data was done with FinalCif.[11] Graphical representations of the crystal structures were created using Mercury (version 2022.3.0). Crystallographic data for the structures reported here have been deposited with the Cambridge Crystallographic Data Centre (CCDC numbers: 2393904 (**1**), 2393903 (**2**), 2393905 (**3**), 2407672 ($\mathbf{4}_{(1)}$), 2407673 ($\mathbf{4}_{(2)}$), 2393906 (**5**), 2407619 (**6a**), 2407671 (**6b**), 2407626 (**7**), 2407624 (**8**), 2407625 (**9**)).[12]

Computational Details

All quantum chemical calculations were performed with the ORCA programme package (version 5.0).[13] Density functional theory (DFT) with the r^{2}SCAN-3c functional[14] and def2-mTZVPP basis set[15] was used with the resolution-of-identity (RI)[16] approximation and dispersion correction (D4).[17] Gibbs free energies of solvation were calculated with the CPCM module[18] at the respective level of theory. All computed structures were checked for the absence of imaginary vibrational frequencies. IR spectra were simulated without scaling factor and a FWHM of 20 cm^{-1} at the RI-r^{2}SCAN-3c(D4)/def2-mTZVPP level of theory.

3. Experimental Procedures, NMR and Vibrational Spectra

3.1. $[Cu(C_6H_6)_3]^+[Al(OR^F)_4]^-$ 1

a) $[(N_2)Cu\{Al(OR^F)_4\}]$ (20.0 mg, 18.9 µmol) was dissolved in benzene (1 mL) which led to a strong gas evolution. The solution was concentrated and then cooled to 4 °C resulting in the formation of yellow crystals of **1**. The crystals are stable under inert atmosphere for months and under perfluoropolyether oil (while selecting crystals for scXRD) for at least one hour.

b) $[NO][Al(OR^F)_4]$ (200 mg, 200 µmol) and copper powder (38 mg, 0.60 mmol, 3.0 equiv.) were dispersed in benzene (2 mL) and stirred overnight at room temperature. The solution was filtered and the solvent was removed under reduced pressure to obtain the product **1** as a yellow solid in poor yields (30 mg, 24 µmol, 12 %). For details on the oxidative preparation see section 3.11.

c) $[NO][Al(OR^F)_4]$ (200 mg, 200 µmol) and copper powder (38 mg, 0.60 mmol, 3.0 equiv.) were dispersed in a mixture of benzene (0.5 mL) and 2FB (2 mL) and stirred overnight at room temperature. The solution was filtered and the solvent was removed under reduced pressure to obtain the product **1** as a yellow solid in good yields (167 mg, 132 µmol, 66 %). For details on the oxidative preparation see section 3.11.

FTIR (ZnSe, ATR): $\tilde{\nu}/cm^{-1}$ = 2959 (vw), 2925 (vw), 2855 (vw), 1618 (vw), 1605 (vw), 1588 (vw), 1544 (vw), 1522 (vw), 1479 (vw), 1467 (vw), 1353 (vw), 1331 (vw), 1301 (w), 1250 (vs), 1221 (s), 1179 (w), 1098 (vw), 1023 (vw), 975 (s), 869 (vw), 792 (vw), 728 (m), 695 (vw), 663 (vw), 610 (vw), 571 (vw).

^{1}H NMR (300.18 MHz, CD_2Cl_2, RT): δ = 7.47 (s., 18 H, $[Cu(C_6\mathbf{H}_6)_3]^+$), *solvent signal at 5.33 ppm*, *minor impurities between 1.33-0.12 ppm stemming from the NMR solvent*.

^{19}F NMR (282.45 MHz, CD_2Cl_2, RT): δ = −75.74 (s., 36 F, $[Al\{OC(C\mathbf{F}_3)_3\}_4]^-$) ppm, *minor impurities at −75.54 and at −75.67 ppm (degradation products of the aluminate anion)*.

^{27}Al NMR (78.22 MHz, CD_2Cl_2, RT): δ = 34.6 (s., 1 Al, $[\mathbf{Al}\{OC(CF_3)_3\}_4]^-$) ppm, *minor impurity at 45.2 ppm (degradation product of the aluminate anion)*.

^{63}Cu NMR (79.59 MHz, CD_2Cl_2, RT): δ = −2404 (br. s., 1 Cu, $[\mathbf{Cu}(C_6H_6)_3]^+$) ppm.

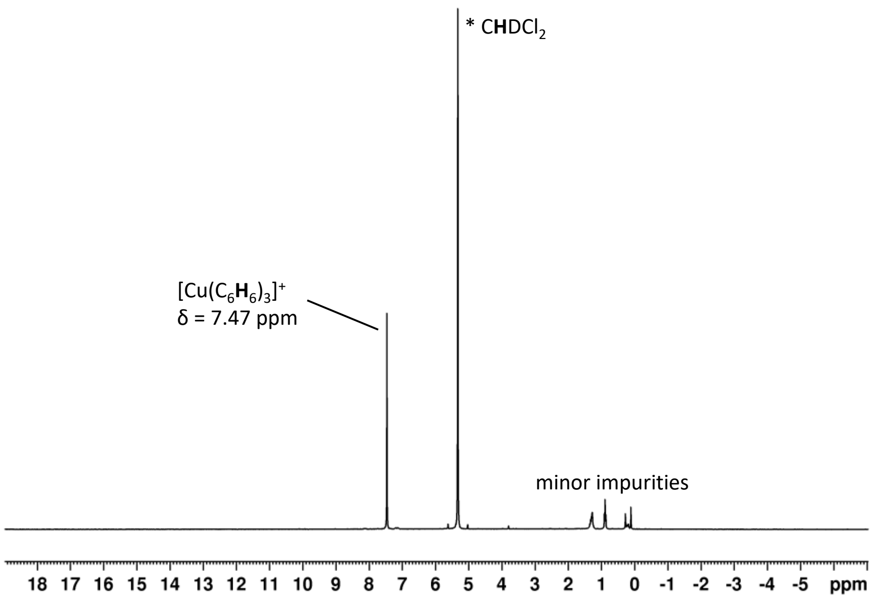

Figure S 1: ^{1}H NMR (300.18 MHz, CD_2Cl_2, RT) spectrum of $[Cu(C_6H_6)_3]^+[Al(OR^F)_4]^-$ **1**. Solvent signal marked with *.

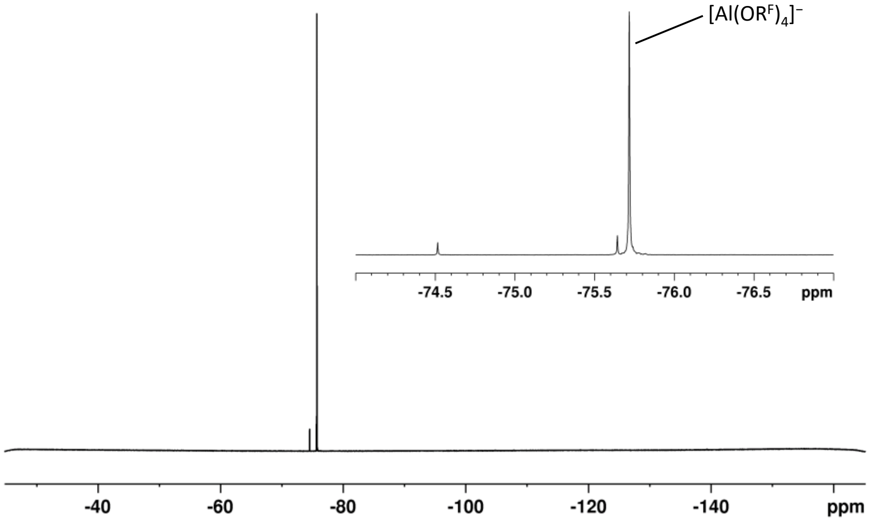

Figure S 2: ^{19}F NMR (282.45 MHz, CD_2Cl_2, RT) spectrum of $[Cu(C_6H_6)_3]^+[Al(OR^F)_4]^-$ **1**.

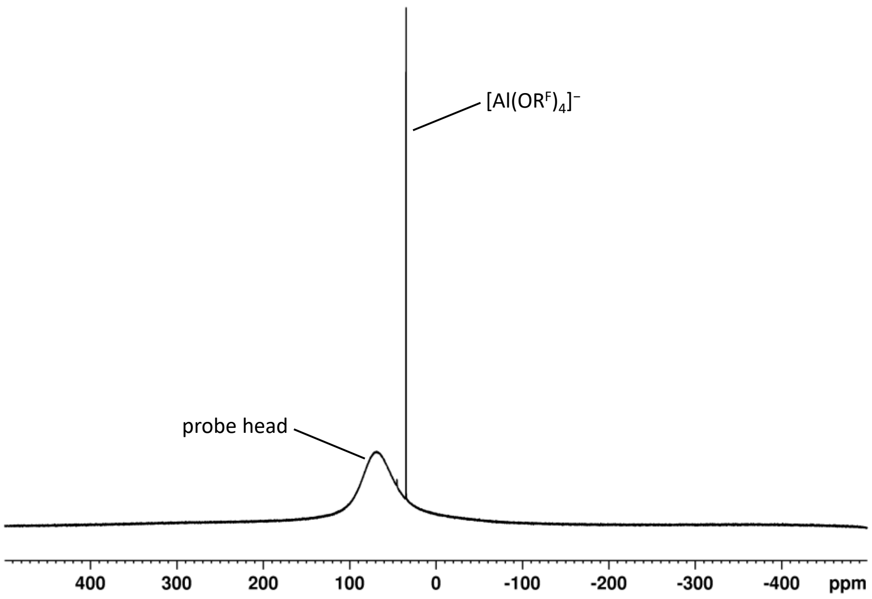

Figure S 3: ^{27}Al NMR (78.22 MHz, CD_2Cl_2, RT) spectrum of $[Cu(C_6H_6)_3]^+[Al(OR^F)_4]^-$ **1**.

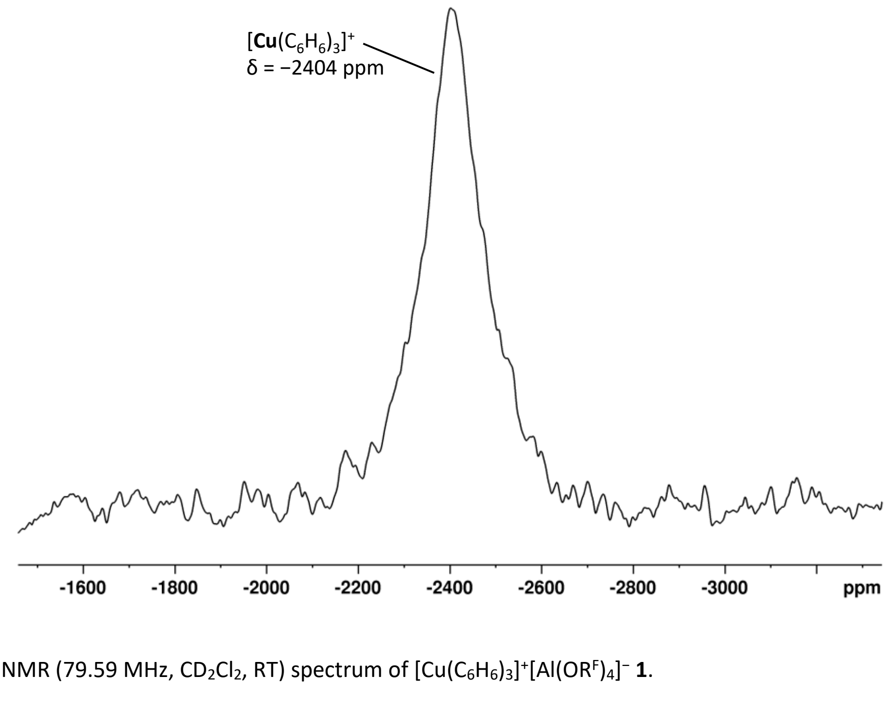

Figure S 4: ^{63}Cu NMR (79.59 MHz, CD_2Cl_2, RT) spectrum of $[Cu(C_6H_6)_3]^+[Al(OR^F)_4]^-$ **1**.

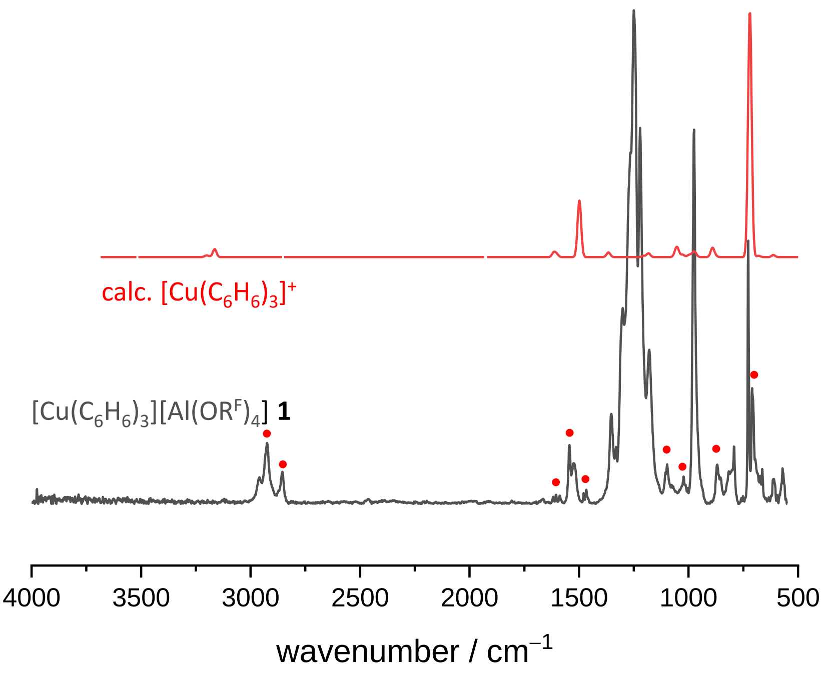

Figure S 5: IR spectrum (32 scans, ZnSe) of $[Cu(C_6H_6)_3][Al(OR^F)_4]$ **1** (black) and calculated spectrum of $[Cu(C_6H_6)_3]^+$ at the r2SCAN-3c/def2-mTZVPP level of theory (red). Cation bands in the experimental spectrum that match the computed spectrum are marked with a red dot. Further bands stem from the anion.

3.2. $[Cu(C_6H_6)_2]^+[Al(OR^F)_4]^-$ 2

a) $[(N_2)Cu\{Al(OR^F)_4\}]$ (25 mg, 24 µmol) was dissolved in 1,2,3,4-tetrafluorobenzene (1 mL) and a solution of benzene in 1,2,3,4-tetrafluorobenzene (0.5 M, 0.1 mL, 48 µmol, 2 equiv.) was added under stirring. The solution was layered with *n*-heptane resulting in the formation of colourless crystals of **2**. While selecting crystals for scXRD under perfluoropolyether oil, the crystals rapidly start decomposing over the course of 5-10 minutes.

b) $[Cu(2FB)_2][Al(OR^F)_4]$ (30 mg, 24 µmol) was dissolved in 1,2,3,4-tetrafluorobenzene (1 mL) and a solution of benzene in 1,2,3,4-tetrafluorobenzene (0.5 M, 0.1 mL, 48 µmol, 2 equiv.) was added under stirring. The solution was layered with *n*-heptane resulting in the formation of colourless crystals of **2**.

3.3. $[Cu(1FB)_3]^+[Al(OR^F)_4]^-$ 3

a) $[(N_2)Cu\{Al(OR^F)_4\}]$ (20.0 mg, 18.9 µmol) was dissolved in fluorobenzene (1 mL) which led to a strong gas evolution. The solution was concentrated and then cooled to −40 °C resulting in the formation of colourless crystals of **3**. The crystals are stable under inert atmosphere for months and under perfluoropolyether oil (while selecting crystals for scXRD) for at least one hour.

b) $[NO][Al(OR^F)_4]$ (200 mg, 200 µmol) and copper powder (38 mg, 0.60 mmol, 3.0 equiv.) were dispersed in fluorobenzene (2 mL) and stirred overnight at room temperature. The solution was filtered and the solvent was removed under reduced pressure to obtain the product **3** as a yellow solid (178 mg, 201 µmol, 67 %). For details on the oxidative preparation see section 3.11.

FTIR (ZnSe, ATR): $\tilde{\nu}/cm^{-1}$ = 3100 (vw), 1595 (vw), 1582 (vw), 1495 (vw), 1483 (vw), 1352 (vw), 1298 (vw), 1238 (m), 1209 (vs), 1155 (w), 1109 (vw), 1060 (vw), 968 (vs), 857 (vw), 830 (vw), 801 (vw), 755 (vw), 725 (vs), 688 (vw), 647 (vw), 560 (vw).

^{1}H NMR (300.18 MHz, CD_2Cl_2, RT): δ = 7.51-7.35 (m, 2 H, $[Cu(C_6\mathbf{H}_5F)]^+$ *meta*-H), 7.25-7.08 (m, 3 H, $[Cu(C_6\mathbf{H}_5F)_3]^+$ *ortho*- and *para*-H), *solvent signal at 5.33 ppm*, *minor impurities between 1.33-0.12 ppm stemming from the solvent*.

^{19}F NMR (282.45 MHz, CD_2Cl_2, RT): δ = −75.75 (s., 36 F, $[Al\{OC(C\mathbf{F}_3)_3\}_4]^-$), −112.02 (m, 3 F, $[Cu(C_6H_5\mathbf{F})_3]^+$) ppm, *minor impurities at −75.55, −75.70, −75.81 and −75.95 ppm (degradation products of the aluminate anion)*.

^{27}Al NMR (78.22 MHz, CD_2Cl_2, RT): δ = 34.6 (s., 1 Al, $[\mathbf{Al}\{OC(CF_3)_3\}_4]^-$) ppm.

^{63}Cu NMR (79.59 MHz, CD_2Cl_2, RT): δ = −2405 (br. s., 1 Cu, $[\mathbf{Cu}(C_6H_6)_3]^+$) ppm.

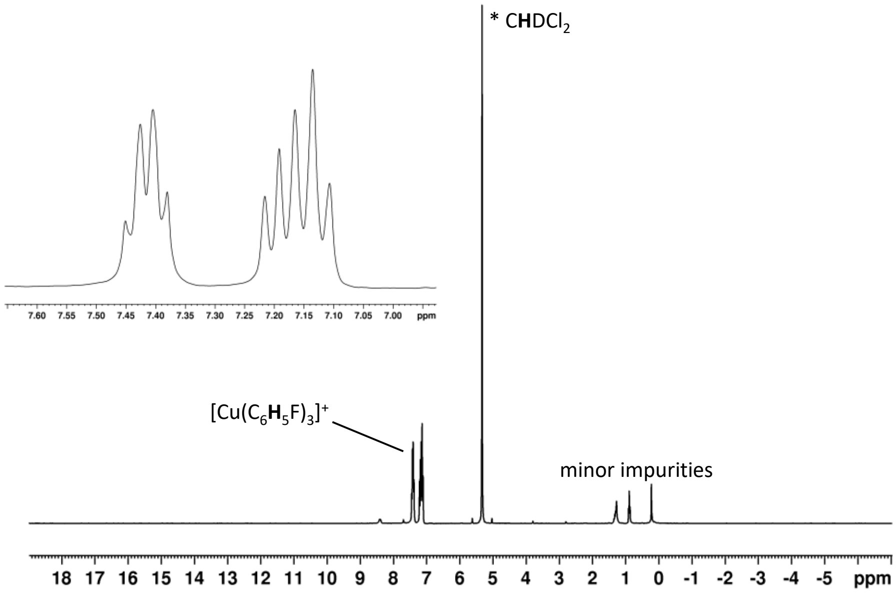

Figure S 6: ^{1}H NMR (300.18 MHz, CD_2Cl_2, RT) spectrum of $[Cu(1FB)_3]^+[Al(OR^F)_4]^-$ **3**. Solvent signal marked with *.

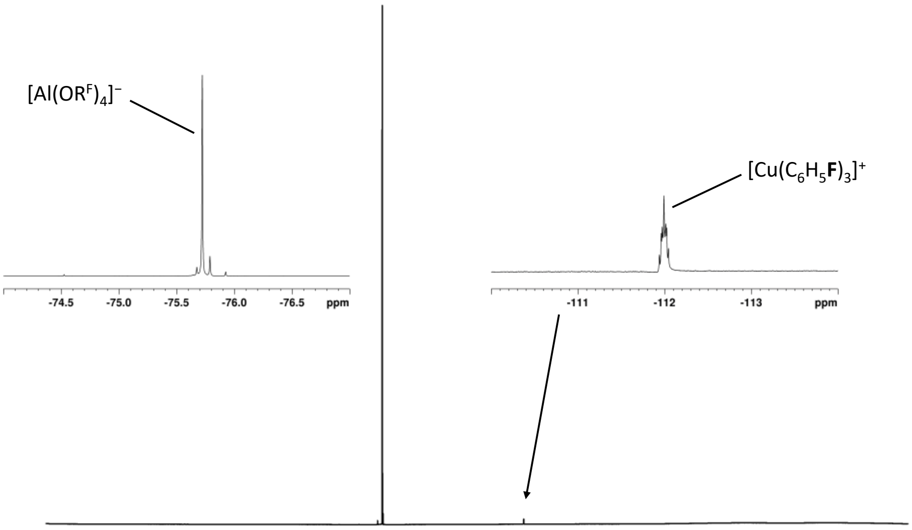

Figure S 7: ^{19}F NMR (282.45 MHz, CD_2Cl_2, RT) spectrum of $[Cu(1FB)_3]^+[Al(OR^F)_4]^-$ **3**. See Figure S 20 for integration of the signals.

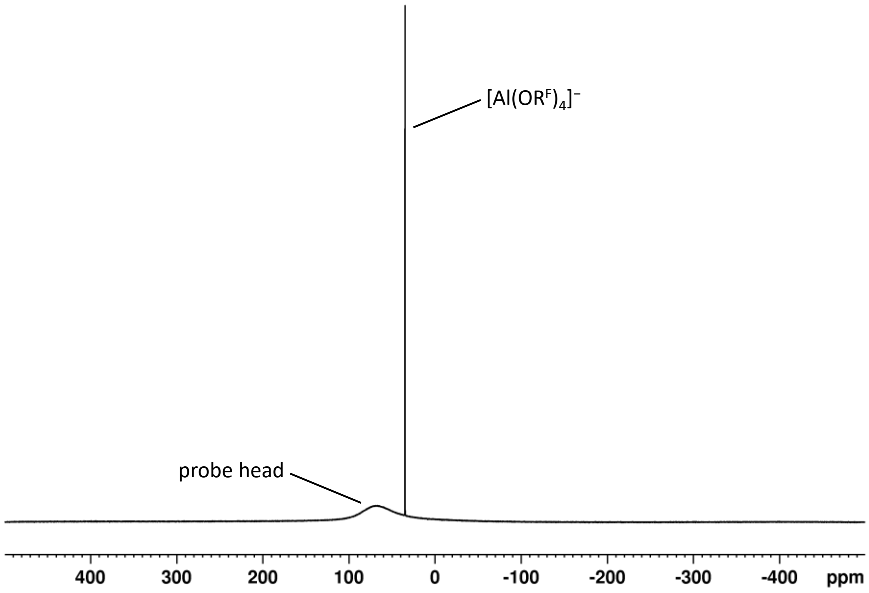

Figure S 8: ^{27}Al NMR (78.22 MHz, CD_2Cl_2, RT) spectrum of $[Cu(1FB)_3]^+[Al(OR^F)_4]^-$ **3**.

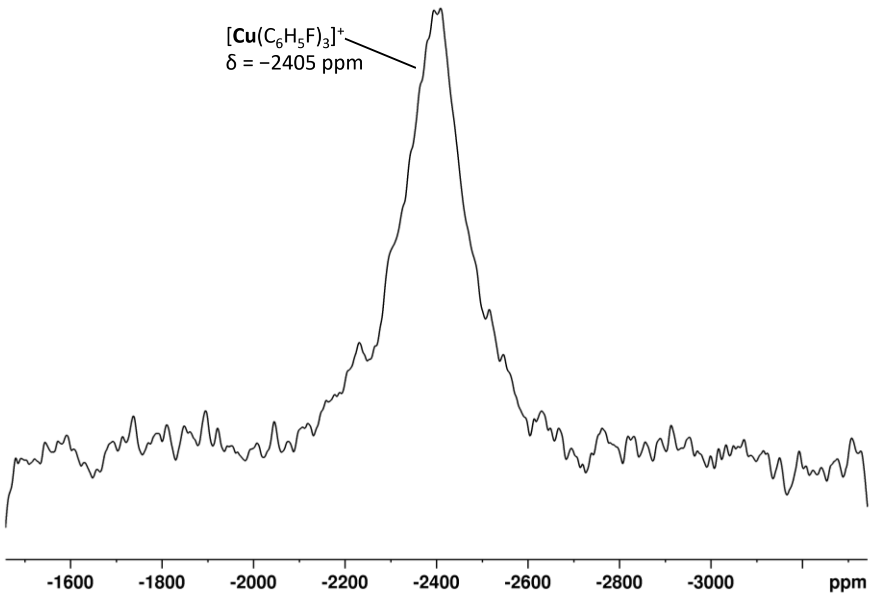

Figure S 9: ^{63}Cu NMR (79.59 MHz, CD_2Cl_2, RT) spectrum of $[Cu(1FB)_3]^+[Al(OR^F)_4]^-$ **3**.

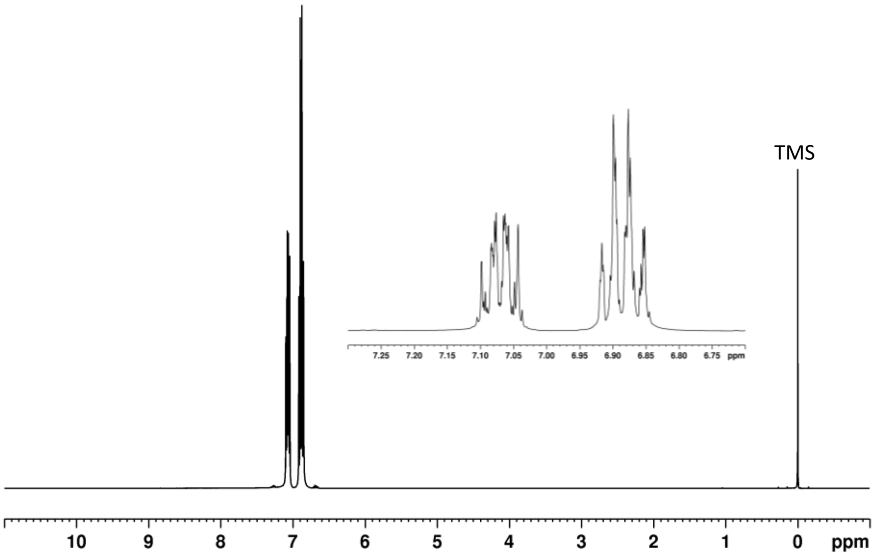

Figure S 10: Reference ^{1}H NMR (400.17 MHz, neat 1FB, RT) spectrum of 1FB with TMS for calibration.

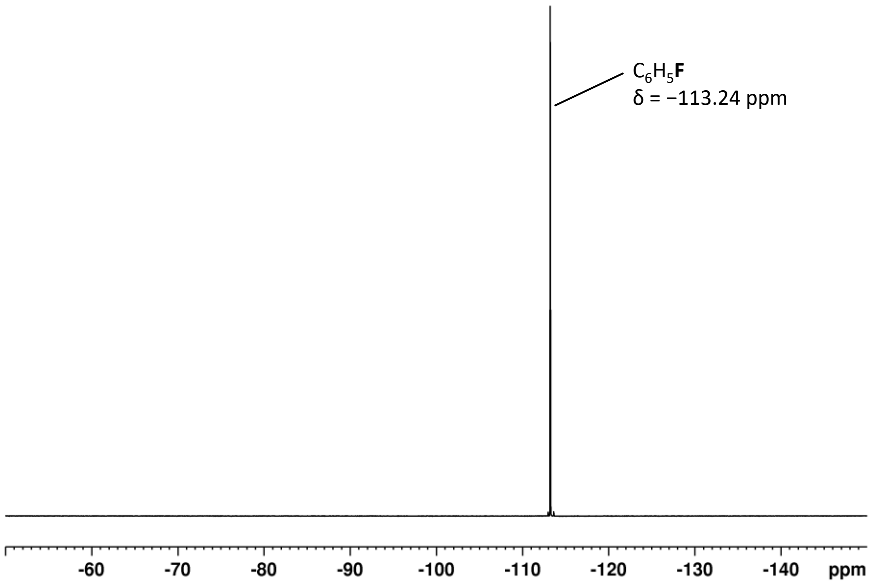

Figure S 11: Reference ^{19}F NMR (376.54 MHz, neat 1FB, RT) spectrum of 1FB.

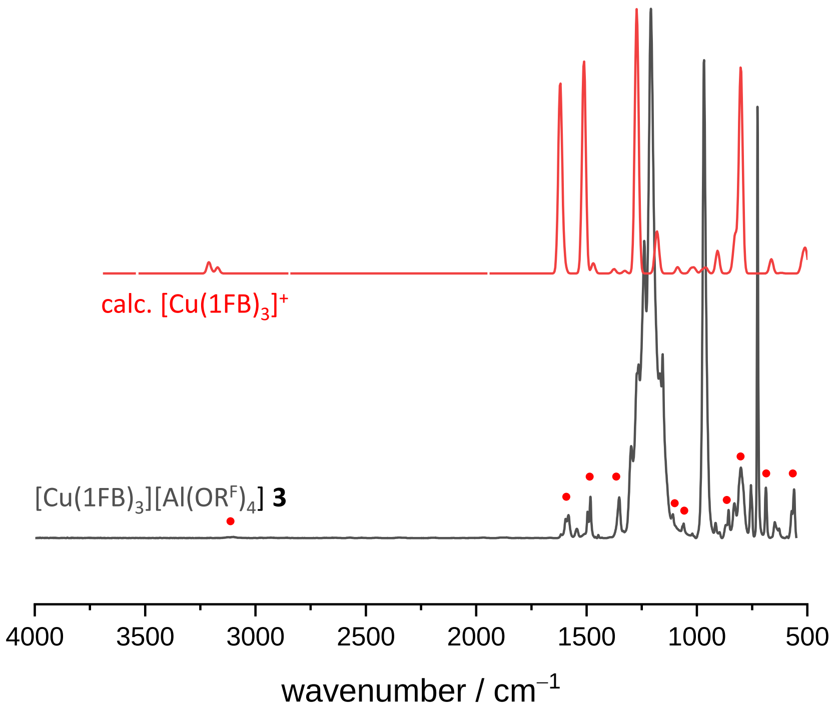

Figure S 12: IR spectrum (32 scans, ZnSe) of $[Cu(1FB)_3][Al(OR^F)_4]$ **3** (black) and calculated spectrum of $[Cu(1FB)_3]^+$ at the r2SCAN-3c/def2-mTZVPP level of theory (red). Cation bands in the experimental spectrum that match the computed spectrum are marked with a red dot. Further bands stem from the anion.

3.4. $[Cu(1FB)_2]^+[Al(OR^F)_4]^-$ 4

$[NO][Al(OR^F)_4]$ (50 mg, 50 µmol) and copper powder (9.6 mg, 0.15 mmol, 3.0 equiv.) were dispersed in 1FB (1 mL) and stirred overnight at room temperature. After ca. 1.5 h of stirring, a colour change from dark red to light yellow was observed. The solution was filtered off from unreacted copper and was layered with *n*-heptane resulting in the formation of colourless crystals of **4**. While selecting crystals for scXRD under perfluoropolyether oil, the crystals rapidly start decomposing over the course of 5-10 minutes.

3.5. $[Cu(3FB)_2]^+[Al(OR^F)_4]^-$ 5

$[(N_2)Cu\{Al(OR^F)_4\}]$ (36.0 mg, 34.0 µmol) was dissolved in fluorobenzene (1 mL) which led to a gas evolution. The solution was layered with *n*-heptane resulting in the formation of colourless crystals of **5**. While selecting crystals for scXRD under perfluoropolyether oil, the crystals rapidly start decomposing over the course of 5-10 minutes.

FTIR (ZnSe, ATR): $\tilde{\nu}/cm^{-1}$ = 3088 (vw), 1582 (vw), 1512 (vw), 1465 (vw), 1352 (vw), 1297 (w), 1240 (s), 1211 (vs), 1174 (w), 1034 (vw), 969 (vs), 829 (vw), 815 (vw), 727 (s), 691 (vw), 560 (vw).

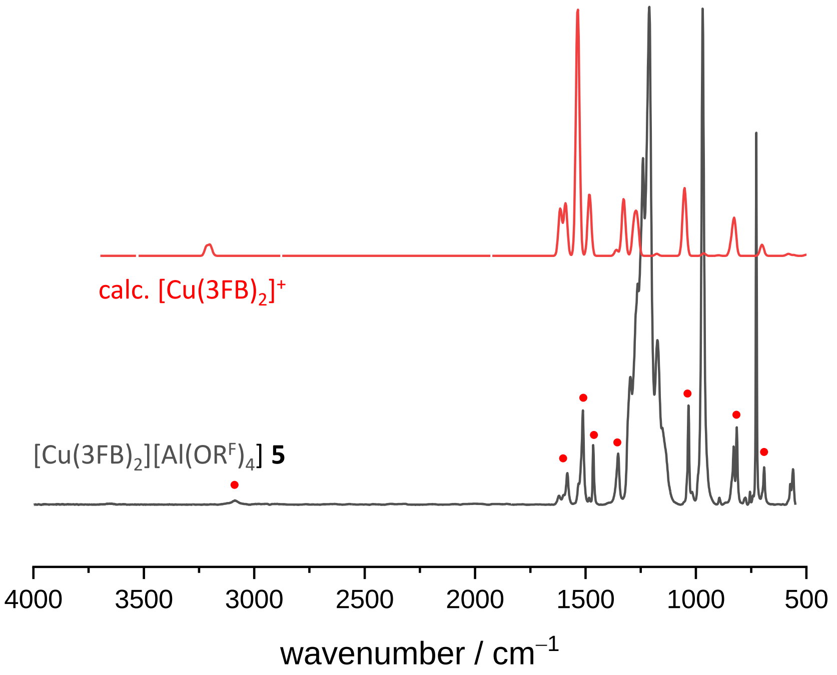

Figure S 13: IR spectrum (32 scans, ZnSe) of $[Cu(3FB)_3][Al(OR^F)_4]$ **5** (black) and calculated spectrum of $[Cu(3FB)_3]^+$ at the r2SCAN-3c/def2-mTZVPP level of theory (red). Cation bands in the experimental spectrum that match the computed spectrum are marked with a red dot. Further bands stem from the anion.

3.6. $[(4FB)Cu\{Al(OR^F)_4\}]$ 6

$[(N_2)Cu\{Al(OR^F)_4\}]$ (50.0 mg, 47.2 µmol) was dissolved in 1,2,3,4-tetrafluorobenzene (1 mL) which led to a gas evolution. To ensure a complete conversion, the solution was degassed with two subsequent freeze-pump-thaw cycles. Then the solution was layered with *n*-heptane and stored at −20 °C resulting in the formation of colourless crystals (modification **6a** featuring a superstructure). Crystals can also be obtained by carefully removing the solvent under reduced pressure, re-dissolving the formed solid in little *iso*-perfluorohexane and cooling the concentrated solution to −20 °C (modification **6b** featuring a 4-component inversion twin). When the target compound is crystallized at room temperature, it quickly decomposes to $[Cu(F\{Al(OR^F)_3\}_2)]$ over the course of one day.

3.7. $[Cu_2(anthracene)_2]^{2+}([Al(OR^F)_4]^-)_2{\cdot}(6FB)_{1.5}$ 7·$(6FB)_{1.5}$

$[Cu(2FB)_2]^+[Al(OR^F)_4]^-$ (100 mg, 80.0 µmol, 1.00 eq.) and anthracene (14.1 mg, 80.0 µmol, 1.00 eq.) were dissolved in 1,2-difluorobenzene (2 mL). The solution was layered with *n*-pentane. Slow diffusion of the solvents led to colourless-yellow crystals of $[Cu_2(anthracene)_2]^{2+}([Al(OR^F)_4]^-)_2$ (63 %, 50.4 µmol, 60.9 mg). Cooling of these crystals to 100 K led to a phase-transition and thus various domains. Still, a data-set of poor quality could be obtained showing the connectivity of the product.

Cell parameters of $[Cu_2(anthracene)_2]^{2+}([Al(OR^F)_4]^-)_2$: a = 11.44 Å, b = 13.03 Å, c = 14.78 Å, α = 74.3°, β = 71.0°, γ = 71.0°.

The crystallization was repeated analogously using a mixture of hexafluorobenzene and 1,2-difluorobenzene yielding single crystals of the composition $[Cu_2(anthracene)_2]^{2+}([Al(OR^F)_4]^-)_2{\cdot}(6FB)_{1.5}$ **7**·$(6FB)_{1.5}$ with better crystallographic data.

FTIR (ZnSe, ATR): $\tilde{\nu}$/cm^{-1} = 1598 (vw), 1442 (vw), 1352 (vw), 1296 (w), 1265 (m), 1239 (s), 1210 (vs), 1172 (w), 968 (vs), 894 (w), 832 (vw), 776 (vw), 756 (vw), 726 (vs), 560 (vw), 537 (w), 473 (vw), 442 (w).

FT Raman (5,000 scans, 25 mW): $\tilde{\nu}$/cm^{-1} = 3086 (w), 2938 (w), 2911 (vw), 2877 (vw), 2752 (vw), 1395 (vs), 1025 (m), 799 (s), 763 (s), 753 (s), 392 (vs).

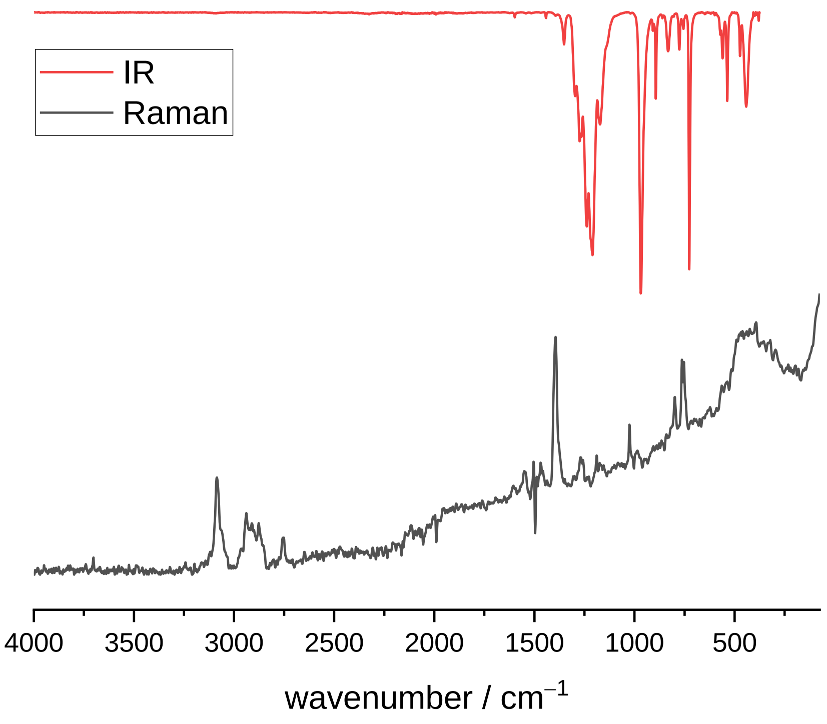

Figure S 14: IR spectrum (32 scans, ZnSe, red) and Raman spectrum (5,000 scans, 25 mW, black) of $[Cu_2(anthracene)_2]^{2+}([Al(OR^F)_4]^-)_2 \cdot (6FB)_{1.5}$ **7**$\cdot(6FB)_{1.5}$.

3.8. $[(Hexaphenylbenzene)\{Cu(2FB)\}_2]^{2+}([Al(OR^F)_4]^-)_2$ 8

Hexaphenylbenzene (13.3 mg, 24.8 µmol, 1.00 eq.) and $[Cu(2FB)_2]^+[Al(OR^F)_4]^-$ (100 mg, 80.0 µmol, 3.20 eq.) were dissolved in 1,2-difluorobenzene (2 mL). The solution was layered with *n*-pentane. Slow diffusion of the solvents led to pale yellow crystals of **8**.

FTIR (ZnSe, ATR): $\tilde{\nu}$/cm^{-1} = 3066 (vw), 2099 (vw), 1589 (vw), 1508 (w), 1352 (w), 1297 (m), 1273 (s), 1239 (s), 1210 (vs), 1163 (m), 1097 (vw), 1025 (vw), 968 (vs), 832 (vw), 782 (w), 791 (w), 764 (w), 733 (w), 725 (vs), 706 (m), 560 (w).

FT Raman (5,000 scans, 25 mW): $\tilde{\nu}$/cm^{-1} = 3069 (vw), 1602 (m), 1588 (m), 1566 (w), 1496 (w), 1345 (vs), 1276 (m), 1216 (m), 1185 (m), 1161 (m), 989 (vs), 797 (s), 764 (s), 745 (s), 711 (s), 661 (s), 620 (s).

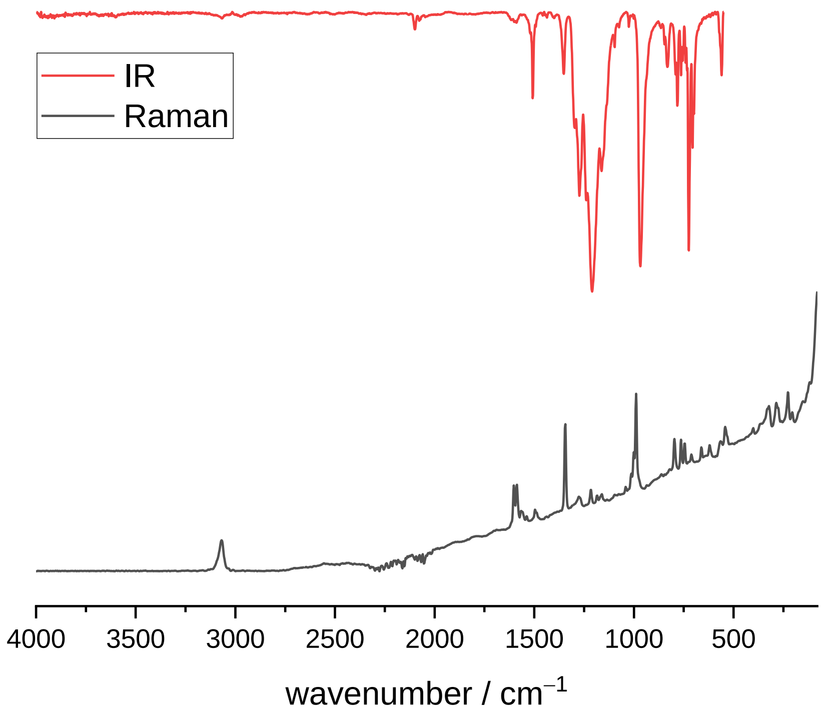

Figure S 15: IR spectrum (32 scans, ZnSe, red) and Raman spectrum (5,000 scans, 25 mW, black) of [(Hexaphenylbenzene){Cu(2FB)}$_2$]$^{2+}$ ([Al(OR^F)$_4$]$^-$)$_2$ **8**.

3.9. [Cu$_3$(hexaphenylbenzene)]$^{3+}$([Al(OR^F)$_4$]$^-$)$_3$·(4FB)$_{2.5}$ 9·(4FB)$_{2.5}$

Hexaphenylbenzene (13.3 mg, 24.8 µmol, 1.00 eq.) and [(N$_2$)Cu{Al(OR^F)$_4$}] (84.1 mg, 79.5 µmol, 3.20 eq.) were filled in a Schlenk-tube in a glovebox. The addition of 1,2,3,4-tetrafluorobenzene (1.5 mL) led to the evolution of a gas. The reaction mixture was stirred until all the solids had dissolved and was layered with *n*-pentane afterwards. Colourless to pale yellow crystals of **9**·(4FB)$_{2.5}$ suitable for scXRD analysis grew over days.

Attempts to isolate products with a higher ratio of copper(I) cations to hexaphenylbenzene by using 6.2 eq. of [(N$_2$)Cu{Al(OR^F)$_4$}] led to the same product.

FTIR (ZnSe, ATR): $\tilde{\nu}$/cm^{-1} = 1513 (vw), 1486 (vw), 1352 (w), 1297 (m), 1274 (m), 1241 (s), 1209 (vs), 1046 (vw), 969 (vs), 833 (vw), 801 (w), 747 (vw), 725 (vs), 703 (w), 682 (vw), 560 (w).

FT Raman (5,000 scans, 25 mW): $\tilde{\nu}$/cm^{-1} = 3067 (w), 1584 (m), 1556 (w), 1491 (w), 1346 (m), 1089 (m), 985 (vs), 797 (s), 745 (s).

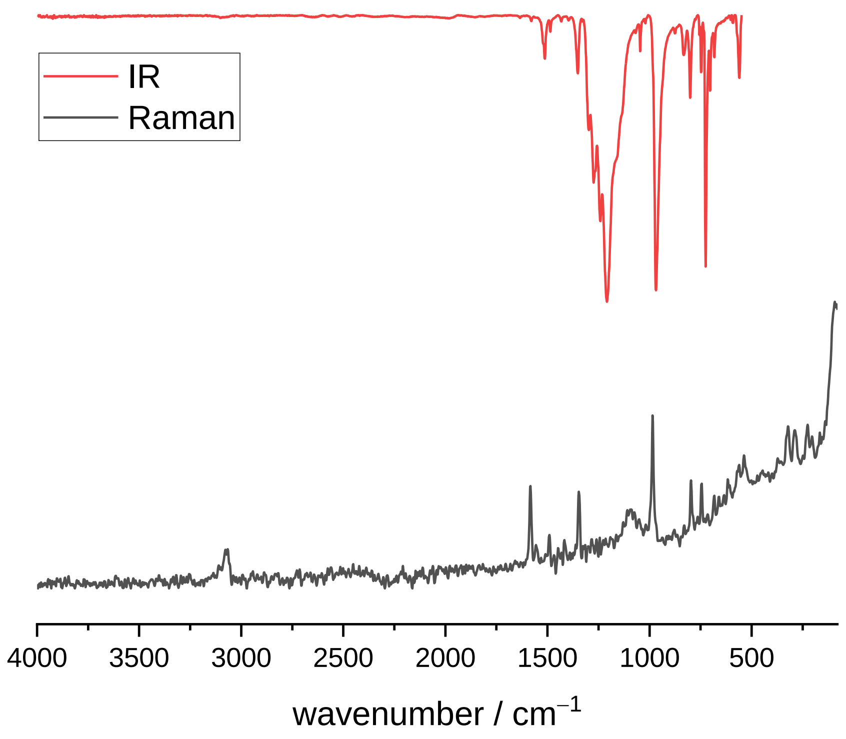

Figure S 16: IR spectrum (32 scans, ZnSe, red) and Raman spectrum (5,000 scans, 25 mW, black) of $[Cu_3(hexaphenylbenzene)]^{3+}([Al(OR^F)_4]^-)_3\cdot(4FB)_{2.5}$ **9**·$(4FB)_{2.5}$.

3.10. Assignment of Vibrational Bands

Table S 1: Assignment of IR and Raman vibrations of compounds **1**, **3**, **5**, **7**, **8** and **9**. The vibrations stemming from the anion are highlighted in darker grey and the assignment of anion bands was done using $[NEt_4]^+[Al(OR^F)_4]^-$ as a reference.[4] Assignment of cation bands was done by visualization of the calculated spectra. Due to the uneven baseline in the experimental Raman spectra of **7**, **8** and **9**, no Raman intensities are given.

1	3	5	7		8		9		$[Al(OR^F)_4]^-$[4]		Assignment[4]
IR	IR	IR	IR	Raman	IR	Raman	IR	Raman	IR	Raman	
			442 (w)						446 (ms)	-	Anion, C–C, C–O
			537 (w)						537 (m)	538 (w)	Anion, C–C, C–O
	560 (vw)	560 (vw)	560 (vw)		560 (w)		560 (w)		562 (mw)	563 (w)	Anion, Al–O, C–C
571 (vw)									571 (w)	-	Anion, Al–O, C–C
610 (vw)						620			-	-	arene out of plane C–C–C deformation, C–Cu
663 (vw)	647 (vw)					661	682 (vw)		-	-	arene out of plane C–C–C deformation
695 (vw)	688 (vw)	691 (vw)			706 (m)	711	703 (w)		-	-	arene out of plane C–C–C deformation
728 (m)	725 (vs)	727 (s)	726 (vs)		725 (vs)		725 (vs)		727 (s)	-	Anion, C–C, C–O
					733 (w)		747 (vw)		-	-	arene out of plane C–C–C, C–C–H deformation
				753		745		745	-	747 (ms)	Anion, Al–O
				763					-	-	anthracene breathing vibration
	755 (vw)		756 (vw)		764 (w)	764			756 (mw)	-	Anion, Al–O
			776 (vw)						-	-	anthracene out of plane C–H deformation
					782 (w)				-	-	arene out of plane C–C–H deformation
792 (vw)					791 (w)	797	801 (w)		-	-	arene out of plane C–C–H deformation
				799				797	-	798 (s)	Anion, Al–O, C–C
869 (vw)	830 (vw)	829 (vw)	832 (vw)		832 (vw)		833 (vw)		833 (m)	834 (w)	Anion, Al–O, C–C
			894 (w)						-	-	anthracene out of plane C–H deformation

975 (s)	968 (vs)	969 (vs)	968 (vs)		968 (vs)	989	969 (vs)	985	973 (s)	978 (mw)	Anion, C–C, C–F
1023 (vw)		1034 (vw)			1025 (vw)		1046 (vw)		-	-	arene in plane C–C, C–F, C–H
1098 (vw)	1060 (vw)			1025	1097 (vw)				-	-	arene in plane C–H
	1109 (vw)								-	-	arene in plane C–H
1179 (w)	1155 (w)	1174 (w)	1172 (w)		1163 (m)				-	-	arene in plane C–H, C–F
						1161		1089	-	1139 (mw)	Anion, C–C, C–F
						1185			-	1173 (mw)	Anion, C–C, C–F
1221 (s)	1209 (vs)	1211 (vs)	1210 (vs)		1210 (vs)	1216	1209 (vs)		1217 (vs)	-	Anion, C–C, C–F
	1238 (m)	1240 (s)	1239 (s)		1239 (s)		1241 (s)		1240 (s)	1235 (mw)	Anion, C–C, C–F
1250 (vs)			1265 (m)						1254 (s)	-	Anion, C–C, C–F
1301 (vw)					1273 (s)	1276	1274 (m)		1274 (vs)	1274 (mw)	Anion, C–C, C–F
1331 (vw)	1298 (vw)	1297 (w)	1296 (w)	1395	1297 (m)	1345	1297 (m)	1346	1298 (s)	1300 (m)	Anion, C–C, C–F
1353 (vw)	1352 (vw)	1352 (vw)	1352 (vw)		1352 (w)		1352 (w)		1353 (ms)	-	Anion, C–C, C–F
			1442 (vw)						-	-	anthracene in plane C–C, C–H
						1496	1486 (vw)	1491	-	-	arene in plane C–C, C–H
1467 (vw)	1483 (vw)	1465 (vw)							-	-	arene in plane C–C, C–H, C–F
1479 (vw)	1495 (vw)								-	-	arene in plane C–C, C–H
1522 (vw)		1512 (vw)			1508 (w)		1513 (vw)		-	-	arene in plane C–C, C–H
1544 (vw)						1566		1556	-	-	arene in plane C–C, C–H
1588 (vw)	1582 (vw)	1582 (vw)			1589 (vw)	1588		1584	-	-	arene in plane C–C, C–H, C–F
			1598 (vw)						-	-	anthracene in plane C–C, C–H
1605 (vw)	1595 (vw)					1602			-	-	arene in plane C–C, C–H, C–F
1618 (vw)									-	-	arene in plane C–C, C–H
					2099 (vw)				-	-	arene in plane C–C, C–H
				2752					-	-	*v* arene C–H
2855 (vw)				2877					-	-	*v* arene C–H
2925 (vw)				2911					-	-	*v* arene C–H
2959 (vw)				2938	3066 (vw)	3069		3067	-	-	*v* arene C–H
	3100 (vw)			3086					-	-	*v* arene C–H
		3088 (vw)							-	-	*v* arene C–F

3.11. Oxidative Syntheses of Cu(I)-Arene Complexes

In Figure S 17, the reaction progress of the oxidation of copper powder using [NO][Al(OR^F)$_4$] is depicted for different solvents (C_6H_6, 1FB, 3FB, 4FB). [NO][Al(OR^F)$_4$] (50 mg, 50 µmol) and copper powder (9.6 mg, 0.15 mmol, 3.0 equiv.) were weighed in glass vials inside a glovebox, then the solvents were added at the same time for the four samples. Every 30 minutes, a picture was taken and the vials were opened to allow formed NO-gas to escape from the system. The fastest reaction took place with 1FB as solvent and ligand: After 30 minutes, the solution started to lose its red colour (caused by the NO-arene Wheland complex) and after 1 h, the reaction was complete. For benzene, it took 2 h to complete the reaction, which is likely caused by the low solubility of the starting material [NO][Al(OR^F)$_4$] in benzene. The reaction in 3FB proceeded even slower. Furthermore, it can be seen that the product is not stable in solution for one day, as the grey colour after 22 h hints at major decomposition. We do not recommend the oxidative synthesis of [Cu(3FB)$_2$]$^+$[Al(OR^F)$_4$]$^-$ **5** with [NO][Al(OR^F)$_4$] as we were not able to reliably reproduce the reaction. In 4FB, no reaction takes place.

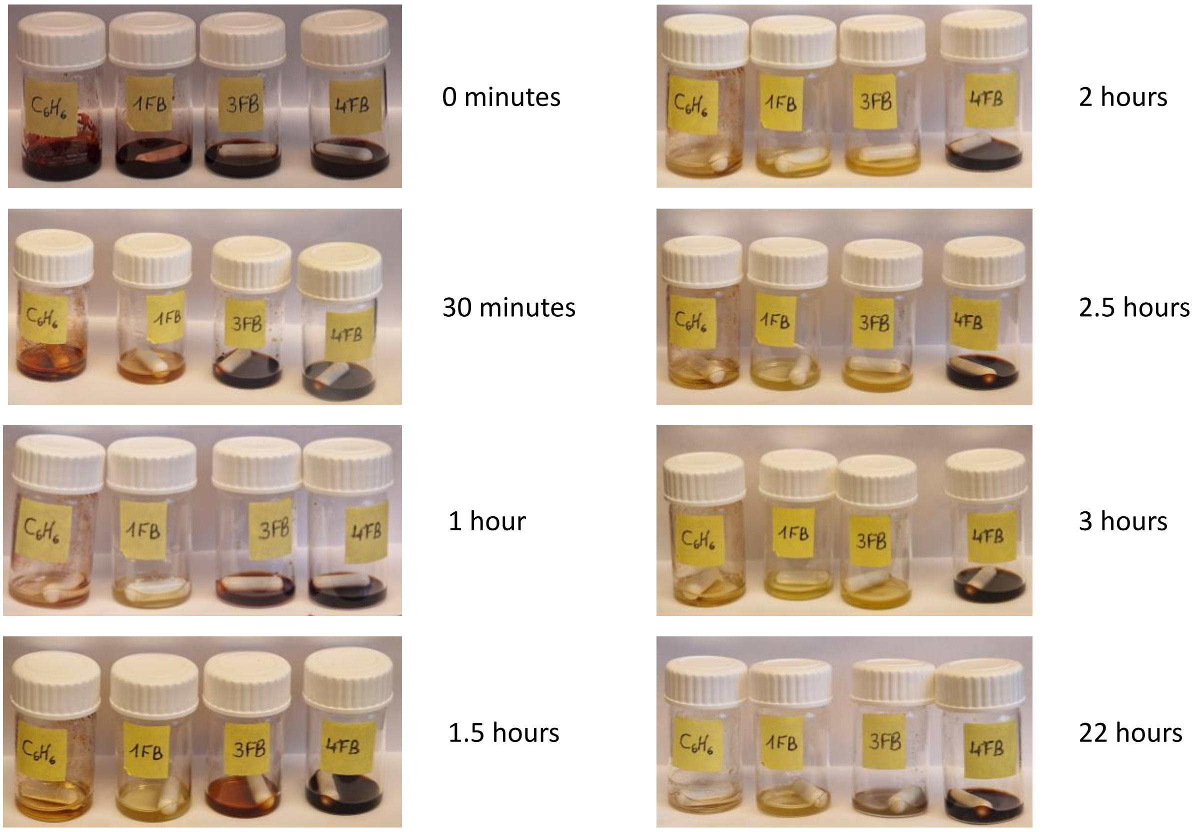

Figure S 17: Reaction of [NO][Al(OR^F)$_4$] (50 mg, 50 µmol) and copper powder (9.6 mg, 0.15 mmol, 3.0 equiv.) in C_6H_6, 1FB, 3FB and 4FB (1 mL each). Pictures taken inside a glovebox every 30 minutes and after one day.

3.12. Determination of the Number of Arene Ligands in 1 and 3 in Solution

The number of arene ligands in **1** could be unambiguously determined by scXRD. Yet, when the oxidative synthesis of **1** using [NO][Al(OR^F)$_4$] was established, we needed a method for bulk analysis of the product to prove that we have indeed synthesized the tris-benzene complex. Furthermore, we were interested if the tris-benzene complex was stable when vacuum is applied to isolate the product and to remove the solvent or if the bis-benzene complex **2** forms or if further decomposition occurs. Yet to prove this by NMR spectroscopy, an internal standard is needed. For this, we chose pentafluorobenzene as it yields well separated signals from **1** in both the ^{1}H and the ^{19}F spectrum and does not react with the product. Furthermore, to ensure that the signals do not overlap, we measured the spectra in acetonitrile-d$_3$, where we suppose that the benzene ligands of **1** are replaced by acetonitrile ligands, for we know that free benzene yields a signal in the ^{1}H NMR spectrum that is well separated from the 5FB signal.

Integration of the benzene signal and the 5FB signal in the ^{1}H NMR spectrum yielded an integral ratio of 0.2980 : 1.000 (Figure S 18). Normalized by the number of protons per molecular entity (6 : 1), the ratio of benzene molecules to 5FB molecules becomes 0.0497 : 1.000. In the ^{19}F NMR spectrum, integration of the anion signal and the signal of the ortho-F atoms of 5FB yielded a ratio of 0.5589 : 2.0000. The ortho-F signal was chosen for integration and calibration of integrals as it is, of all the signals for 5FB, the closest to the offset chosen for the measurement and thus the most reliably integrated. Normalization leads of a ratio of anion to 5FB molecule of 0.0155 : 1.0000. The ratio of anion molecules to benzene molecules consequently is 0.0155 : 0.0497 or 1.0 : 3.2. Thus, it could be shown, that indeed the tris-benzene complex is obtained from the reaction.

For the tris-fluorobenzene complex **3**, the number of ligands can be easily obtained by comparing integrals in the ^{19}F NMR spectrum of the anion and of the 1FB signal.

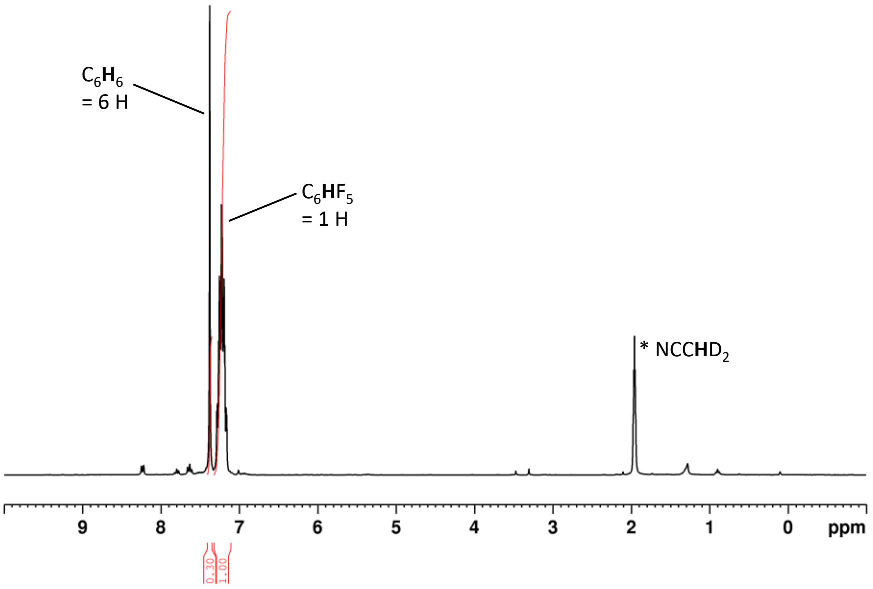

Figure S 18: ^{1}H NMR (300.18 MHz, acetonitrile-d$_3$ + 16 µL pentafluorobenzene, RT) spectrum of $[Cu(C_6H_6)_3]^+[Al(OR^F)_4]^-$ **1** with integrals shown. The 5FB signal was used for calibration. Solvent signal marked with *, impurities such as water and dimethoxy ethane stem from the NMR solvent.

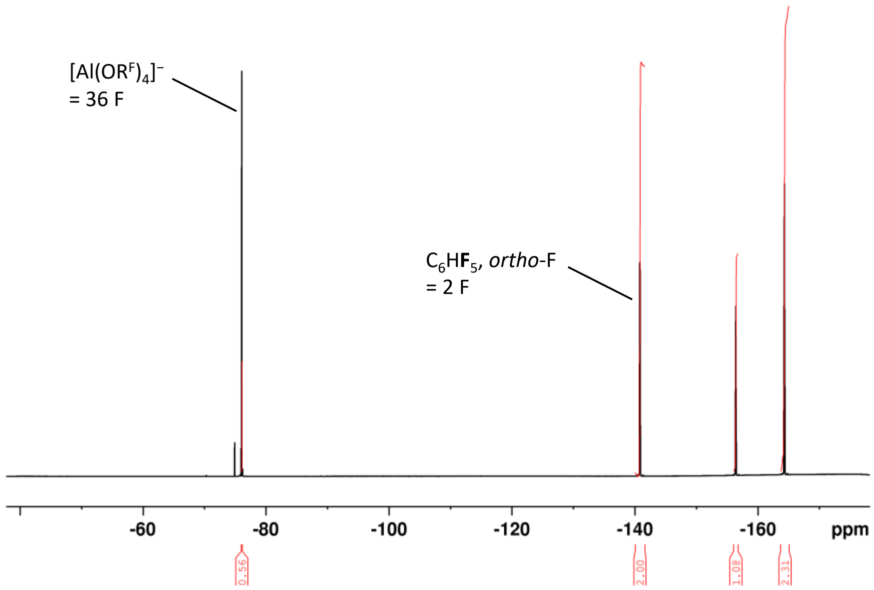

Figure S 19: ^{19}F NMR (282.45 MHz, acetonitrile-d$_3$ + 16 µL pentafluorobenzene, RT) spectrum of $[Cu(C_6H_6)_3]^+[Al(OR^F)_4]^-$ **1** with integrals shown. The 5FB, *ortho*-F, signal was used for calibration.

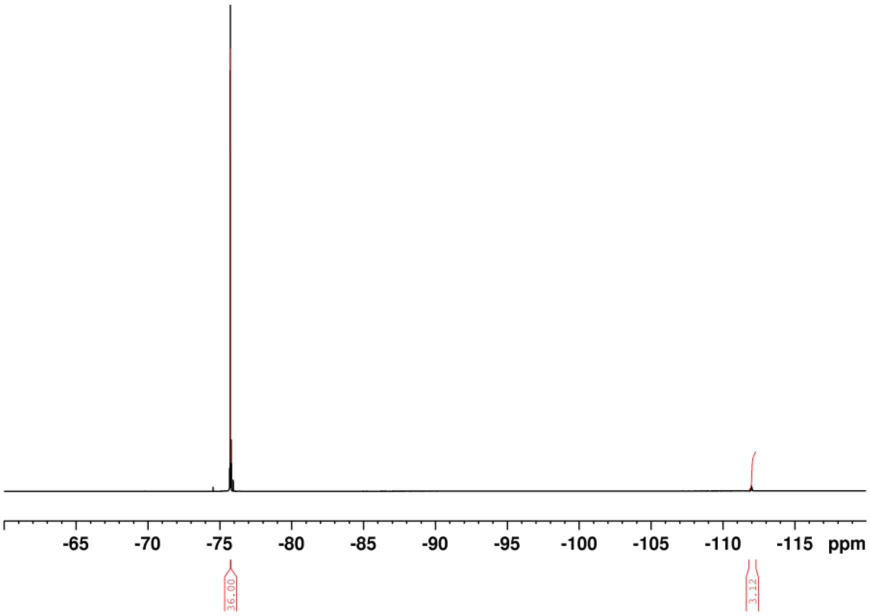

Figure S 20: ^{19}F NMR (282.45 MHz, CD_2Cl_2, RT) spectrum of $[Cu(1FB)_3]^+[Al(OR^F)_4]^-$ **3** with integrals shown. The anion signal was used for calibration.

4. Single-Crystal XRD Data

Table S 2: Crystallographic data of $[Cu(C_6H_6)_3]^+[Al(OR^F)_4]^-$ **1**, $[Cu(C_6H_6)_2]^+[Al(OR^F)_4]^-$ **2** and $[Cu(1FB)_3]^+[Al(OR^F)_4]^-$ **3**.

	**1**	**2**	**3**
**CCDC number**	2393904	2393903	2393905
**Empirical formula**	$C_{34}H_{18}AlCuF_{36}O_4$	$C_{28}H_{12}AlCuF_{36}O_4$	$C_{34}H_{15}AlCuF_{39}O_4$
**Formula weight**	1265.00	1186.90	1318.98
**Temperature [K]**	200(2)	100(2)	200(2)
**Crystal system**	orthorhombic	triclinic	orthorhombic
**Space group (number)**	$Pna2_1$ (33)	$P\overline{1}$ (2)	$Pna2_1$ (33)
a **[Å]**	24.044(4)	9.684(4)	23.822(7)
b **[Å]**	10.6332(17)	13.927(6)	10.708(3)
c **[Å]**	17.117(2)	14.664(6)	17.283(7)
**α [°]**	90	89.179(16)	90
**β [°]**	90	81.224(16)	90
**γ [°]**	90	83.122(12)	90
**Volume [Å³]**	4376.2(11)	1940.6(13)	4409(3)
Z	4	2	4
ρ_{calc} **[g cm⁻³]**	1.920	2.031	1.987
μ **[mm⁻¹]**	0.713	0.796	0.721
***F*(000)**	2480	1156	2576
**Crystal size [mm³]**	0.410×0.246×0.234	0.162×0.129×0.058	0.344×0.204×0.121
**Crystal colour**	yellow	colourless	colourless
**Crystal shape**	block	plate	block
**Radiation**	MoK_α (λ=0.71073 Å)	MoK_α (λ=0.71073 Å)	MoK_α (λ=0.71073 Å)
**2θ range [°]**	4.14 to 54.45 (0.78 Å)	2.81 to 53.91 (0.78 Å)	4.15 to 50.34 (0.84 Å)
**Index ranges**	−30 ≤ h ≤ 30<br>−13 ≤ k ≤ 13<br>−21 ≤ l ≤ 21	−12 ≤ h ≤ 12<br>−17 ≤ k ≤ 17<br>−18 ≤ l ≤ 18	−28 ≤ h ≤ 28<br>−12 ≤ k ≤ 12<br>−20 ≤ l ≤ 20
**Reflections collected**	136588	64593	117523
**Independent reflections**	9702<br>R_{int} = 0.0936<br>R_{sigma} = 0.0365	8310<br>R_{int} = 0.0586<br>R_{sigma} = 0.0341	7869<br>R_{int} = 0.0789<br>R_{sigma} = 0.0385
**Completeness to θ = 25.242° / 25.242°**	99.9 %	99.8 %	99.6 %
**Data / Restraints / Parameters**	9702/6014/922	8310/4792/768	7869/6173/968
**Goodness-of-fit on F^2**	1.029	1.048	1.030
**Final *R* indexes [*I* ≥ 2σ(*I*)]**	R_1 = 0.0778<br>wR_2 = 0.2151	R_1 = 0.0899<br>wR_2 = 0.2637	R_1 = 0.0646<br>wR_2 = 0.1564
**Final *R* indexes [all data]**	R_1 = 0.1020<br>wR_2 = 0.2453	R_1 = 0.1064<br>wR_2 = 0.2774	R_1 = 0.0952<br>wR_2 = 0.1823
**Flack X parameter**	0.011(7)	–	0.00(4)
**Largest peak/hole [eÅ⁻³]**	0.70/−0.40	1.30/−1.12	0.52/−0.40

Table S 3: Crystallographic data of $[Cu(1FB)_2]^+[Al(OR^F)_4]^-$ **4** treated as **4$_{(1)}$** and **4$_{(2)}$**, $[Cu(3FB)_2]^+[Al(OR^F)_4]^-$ **5**.

	**4$_{(1)}$**	**4$_{(2)}$**	**5**
**CCDC number**	2407672	2407673	2393906
**Empirical formula**	$C_{28}H_{10}AlCuF_{38}O_4$	$C_{28}H_{10}AlCuF_{38}O_4$	$C_{28}H_6AlCuF_{42}O_4$
**Formula weight**	1222.88	1222.88	1294.85
**Temperature [K]**	100(2)	100(2)	100(2)
**Crystal system**	triclinic	triclinic	monoclinic
**Space group (number)**	$P\overline{1}$ (2)	$P\overline{1}$ (2)	$P2_1/c$ (14)
a **[Å]**	18.403(6)	9.759(4)	10.212(4)
b **[Å]**	21.379(6)	10.695(3)	18.408(5)
c **[Å]**	22.307(8)	20.538(8)	20.739(9)
**α [°]**	66.538(11)	84.965(11)	90
**β [°]**	77.285(10)	79.446(16)	97.539(17)
**γ [°]**	79.461(12)	67.938(13)	90
**Volume [Å^3]**	7809(5)	1952.5(12)	3865(2)
Z	8	2	4
**ρ_{calc} [g cm^{-3}]**	2.080	2.080	2.225
**μ [mm^{-1}]**	0.802	0.801	0.831
***F*(000)**	4752	1188	2504
**Crystal size [mm^3]**	0.070×0.147×0.270	0.070×0.147×0.270	0.335×0.219×0.091
**Crystal colour**	colourless	colourless	colourless
**Crystal shape**	plate	plate	plate
**Radiation**	MoK_α (λ=0.71073 Å)	MoK_α (λ=0.71073 Å)	MoK_α (λ=0.71073 Å)
**2θ range [°]**	2.28 to 51.71 (0.81 Å)	4.04 to 51.68 (0.82 Å)	2.97 to 54.39 (0.78 Å)
**Index ranges**	−22 ≤ h ≤ 22	−11 ≤ h ≤ 11	−13 ≤ h ≤ 13
	−26 ≤ k ≤ 26	−13 ≤ k ≤ 13	−23 ≤ k ≤ 23
	−27 ≤ l ≤ 27	−25 ≤ l ≤ 25	−26 ≤ l ≤ 26
**Reflections collected**	356951	87880	159782
**Independent reflections**	29914	7444	8578
	R_{int} = 0.1237	R_{int} = 0.0606	R_{int} = 0.0704
	R_{sigma} = 0.0651	R_{sigma} = 0.0292	R_{sigma} = 0.0213
**Completeness to θ = 25.242°**	100.0 %	100.0 %	100.0 %
**Data / Restraints / Parameters**	29914/69327/3653	7444 / 21101 / 1761	8578/4532/895
**Goodness-of-fit on *F*^{2}**	1.021	1.029	1.041
**Final *R* indexes [*I* ≥ 2σ(*I*)]**	R_1 = 0.0897	R_1 = 0.0803	R_1 = 0.0417
	wR_2 = 0.2394	wR_2 = 0.2197	wR_2 = 0.0875
**Final *R* indexes [all data]**	R_1 = 0.1316	R_1 = 0.0998	R_1 = 0.0575
	wR_2 = 0.2876	wR_2 = 0.2422	wR_2 = 0.0977
**Largest peak/hole [eÅ^{-3}]**	1.64/−0.66	0.91/−0.94	1.08/−1.14

Table S 4: Crystallographic data of both modifications of [(4FB)Cu{Al(OR^F)$_4$}] **6a** and **6b**, [Cu_2(anthracene)$_2$]$^{2+}$([Al(OR^F)$_4$]$^-$)$_2$·(6FB)$_{1.5}$ **7**·(6FB)$_{1.5}$.

	**6a**[a]	**6b**	**7**·(6FB)$_{1.5}$
**CCDC number**	2407619	2407671	2407626
**Empirical formula**	$C_{22}H_2AlCuF_{40}O_4$	$C_{22}H_2AlCuF_{40}O_4$	$C_{69}H_{20}Al_2Cu_2F_{81}O_8$
**Formula weight**	1180.76	1180.76	2696.89
**Temperature [K]**	100(2)	100(2)	100(2)
**Crystal system**	monoclinic	monoclinic	triclinic
**Space group (number)**	$P2_1/n$ (14)	Cc (9)	$P\overline{1}$ (2)
***a* [Å]**	20.805(4)	37.121(9)	14.790(3)
***b* [Å]**	16.976(4)	20.971(5)	15.519(3)
***c* [Å]**	38.867(10)	20.955(5)	19.409(4)
**α [°]**	90	90	99.659(12)
**β [°]**	95.870(9)	124.424(10)	99.874(15)
**γ [°]**	90	90	98.083(16)
**Volume [Å^3]**	13656(6)	13456(6)	4260.5(14)
Z	16	16	2
**ρ_{calc} [g cm^{-3}]**	2.297	2.331	2.102
**μ [mm^{-1}]**	0.920	0.934	0.754
***F*(000)**	9088	9088	2622
**Crystal size [mm^3]**	0.460×0.276×0.206	0.106×0.159×0.209	0.145×0.194×0.238
**Crystal colour**	yellow	colourless	colourless
**Crystal shape**	block	block	plate
**Radiation**	MoK_α (λ=0.71073 Å)	MoK_α (λ=0.71073 Å)	MoK_α (λ=0.71073 Å)
**2θ range [°]**	2.62 to 61.18 (0.70 Å)	2.75 to 52.82 (0.80 Å)	2.70 to 55.93 (0.76 Å)
**Index ranges**	−29 ≤ h ≤ 29	−46 ≤ h ≤ 46	−19 ≤ h ≤ 19
	−24 ≤ k ≤ 24	−26 ≤ k ≤ 26	−20 ≤ k ≤ 20
	−55 ≤ l ≤ 55	−26 ≤ l ≤ 26	−25 ≤ l ≤ 25
**Reflections collected**	784773	152353	241289
**Independent reflections**	41896	27217	20441
	R_{int} = 0.1110	R_{int} = 0.0695	R_{int} = 0.0506
	R_{sigma} = 0.0457	R_{sigma} = 0.0592	R_{sigma} = 0.0209
**Completeness to θ = 25.242°**	100.0 %	100.0 %	99.9 %
**Data / Restraints / Parameters**	41896/86438/3566	27217 / 79799 / 3341	20441 / 19598 / 1970
**Goodness-of-fit on F^2**	1.120	1.024	1.061
**Final *R* indexes [$I \geq 2\sigma(I)$]**	R_1 = 0.1069	R_1 = 0.0548	R_1 = 0.0357
	wR_2 = 0.2484	wR_2 = 0.1204	wR_2 = 0.0862
**Final *R* indexes [all data]**	R_1 = 0.1366	R_1 = 0.0794	R_1 = 0.0513
	wR_2 = 0.2645	wR_2 = 0.1345	wR_2 = 0.0971
**Largest peak/hole [eÅ^{-3}]**	2.11/−1.10	0.70/−0.83	0.42/−0.70

[a] Structure already preliminarily published as a picture in the supporting info in reference[2]. No crystallographic data provided to date.

Table S 5: Crystallographic data of $[(HPB)\{Cu(2FB)\}_2]^{2+}([Al(OR^F)_4]^-)_2$ **8** and $[Cu_3(HPB)]^{3+}([Al(OR^F)_4]^-)_3\cdot(4FB)_{2.5}$ **9**$\cdot(4FB)_{2.5}$.

	**8**	**9**$\cdot(4FB)_{2.5}$
**CCDC number**	2407624	2407625
**Empirical formula**	$C_{86}H_{38}Al_2Cu_2F_{76}O_8$	$C_{105}H_{35}Al_3Cu_3F_{118}O_{12}$
**Formula weight**	2824.20	4001.89
**Temperature [K]**	100(2)	100(2)
**Crystal system**	triclinic	triclinic
**Space group (number)**	$P\overline{1}$ (2)	$P\overline{1}$ (2)
a **[Å]**	12.383(3)	15.687(13)
b **[Å]**	14.458(5)	18.16(2)
c **[Å]**	15.845(6)	23.551(12)
**α [°]**	69.287(18)	86.26(3)
**β [°]**	69.249(9)	77.60(2)
**γ [°]**	77.076(18)	79.71(5)
**Volume [Å^3]**	2465.4(15)	6443(10)
Z	1	2
ρ_{calc} **[g cm^{-3}]**	1.902	2.063
μ **[mm^{-1}]**	0.649	0.743
***F*(000)**	1386	3898
**Crystal size [mm^3]**	0.136×0.179×0.193	0.113×0.136×0.195
**Crystal colour**		colourless
**Crystal shape**	block	block
**Radiation**	MoK_α (λ=0.71073 Å)	MoK_α (λ=0.71073 Å)
**2θ range [°]**	2.88 to 56.79 (0.75 Å)	2.70 to 57.02 (0.74 Å)
**Index ranges**	−15 ≤ h ≤ 16	−21 ≤ h ≤ 21
	−19 ≤ k ≤ 19	−24 ≤ k ≤ 24
	−21 ≤ l ≤ 21	−31 ≤ l ≤ 31
**Reflections collected**	138763	324151
**Independent reflections**	12334	32462
	R_{int} = 0.1254	R_{int} = 0.0603
	R_{sigma} = 0.0426	R_{sigma} = 0.0332
**Completeness to θ = 25.242°**	99.9 %	99.9 %
**Data / Restraints / Parameters**	12334 / 8805 / 1165	32462 / 109048 / 4218
**Goodness-of-fit on F^2**	1.107	1.030
**Final *R* indexes [$I \geq 2\sigma(I)$]**	R_1 = 0.0477	R_1 = 0.0649
	wR_2 = 0.1193	wR_2 = 0.1761
**Final *R* indexes [all data]**	R_1 = 0.0545	R_1 = 0.0909
	wR_2 = 0.1259	wR_2 = 0.1994
**Largest peak/hole [eÅ^{-3}]**	0.89/−1.39	0.84/−0.79

Details on the structures of **1** and **3**

1 and **3** crystallize isostructurally and both feature a superstructure which is visible by rows of reflexes with alternating intensity in the reciprocal space. At 100 K, the superstructure is more pronounced than at higher temperatures where the formerly ordered structure becomes statistically disordered. Therefore, the crystals were measured at 200 K. Remnants of the superstructure still remain at this temperature but they could be described as disorders and this temperature was chosen as a compromise, as at higher temperatures, the thermal motion of the atoms increases and the maximally attainable resolution diminishes. The structures were solved and refined using both intense and less intense reflexes resulting in a larger cell (supercell).

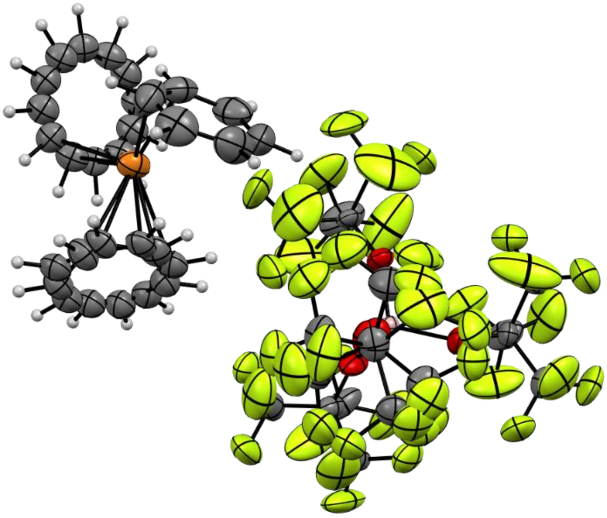

Figure S 21: Molecular structure of $[Cu(C_6H_6)_3]^+[Al(OR^F)_4]^-$ **1** with disordered moieties shown (occupancies of disordered benzene 60/40). Thermal ellipsoids are set at the 50 % probability level. As the crystals suffer from a superstructure, scXRD measurements were carried out at higher temperatures (200 K) resulting in larger thermal ellipsoids. Remnants of the superstructures still remain at 200 K and the structure was solved in an orthorhombic supercell (*Pna*2_1, a = 24.04 Å, b = 10.63 Å, c = 17.12 Å).

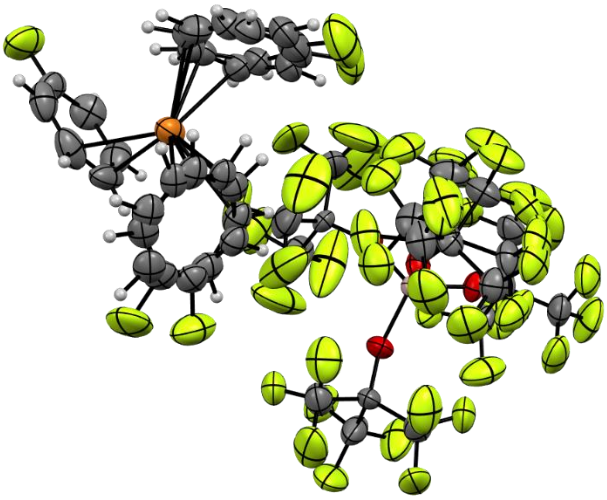

Figure S 22: Molecular structure of $[Cu(1FB)_3]^+[Al(OR^F)_4]^-$ **3** with disordered moieties shown (occupancies of disordered 1FB 65/35). Thermal ellipsoids are set at the 50 % probability level. As the crystals suffer from a superstructure, scXRD measurements were carried out at higher temperatures (200 K) resulting in larger thermal ellipsoids. Remnants of the superstructures still remain at 200 K and the structure was solved in an orthorhombic supercell (*Pna*2_1, a = 23.82 Å, b = 10.71 Å, c = 17.28 Å).

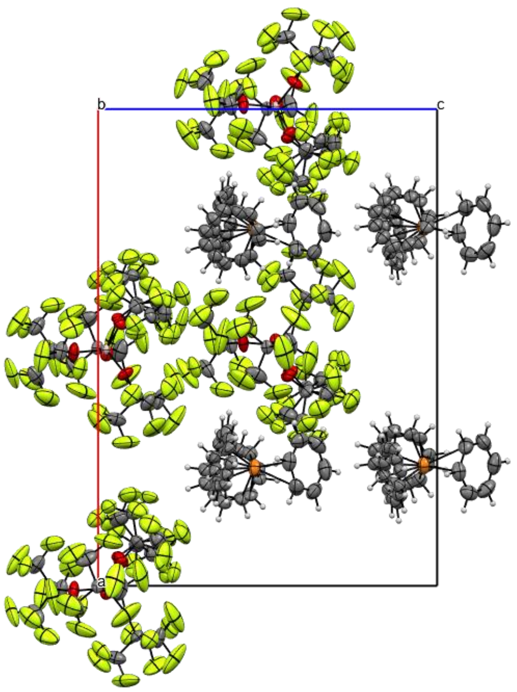

Figure S 23: Packed unit cell for the structure of $[Cu(C_6H_6)_3]^+[Al(OR^F)_4]^-$ **1**.

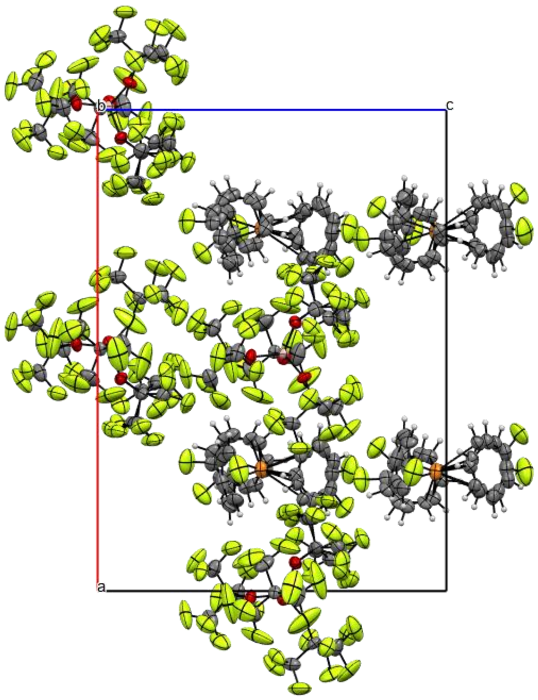

Figure S 24: Packed unit cell for the structure of $[Cu(1FB)_3]^+[Al(OR^F)_4]^-$ **3**.

Details on the structure of **2**

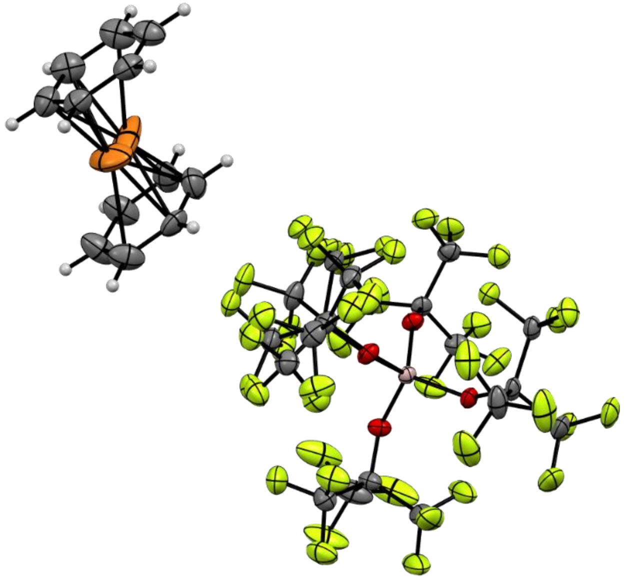

Figure S 25: Molecular structure of $[Cu(C_6H_6)_2]^+[Al(OR^F)_4]^-$ **2** with disordered moieties shown (occupancy of disordered copper 50/50). Thermal ellipsoids are set at the 50 % probability level.

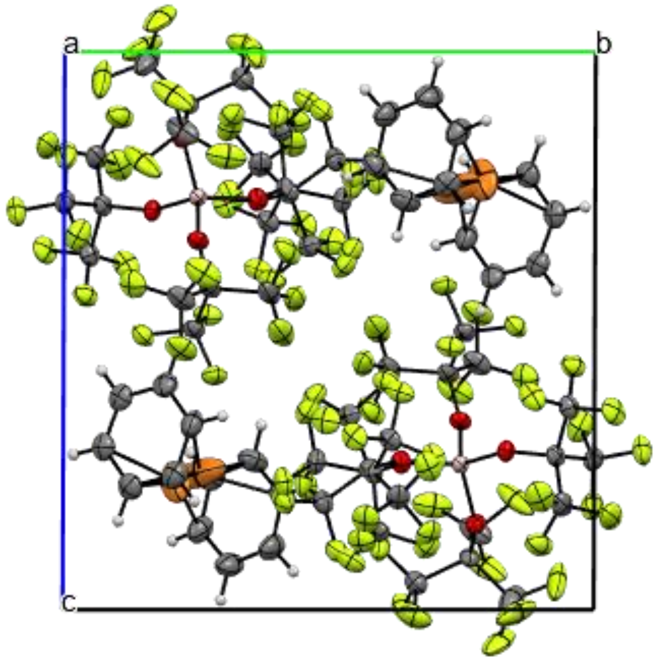

Figure S 26: Packed unit cell for the structure of $[Cu(C_6H_6)_2]^+[Al(OR^F)_4]^-$ **2**.

Details on the structure of **4**

The crystallographic data for **4** was treated in two different ways (**4**$_{(1)}$ and **4**$_{(2)}$ - note that these are not different modifications! The data stems from the very same crystal and only differs in the different unit cell determination).

First, all reflexes were considered, resulting in a large unit cell ($P\overline{1}$, a = 18.40 Å, b = 21.38 Å, c = 22.31 Å, α = 66.54 °, β = 77.29 °, γ = 79.46 °, V = 7809 Å^3, Z = 8) and the structure **4**$_{(1)}$. The structure was refined treating the cationic moieties not as molecular entities but individually as disordered fluorobenzene ligands and disordered copper atoms with independent free variables. A molecular treatment led to a significantly worse model. While the structure seemed to be valid at first look, a closer look at a large amount of unexplainable undescribed electron density on the aluminate anions and especially the aluminium atoms hinted at a so far undiscovered problem with the structure.

A closer look at the reciprocal lattice (Figure S 27) revealed the presence of a superstructure. For the second solution, **4**$_{(2)}$, only the strong reflexes (red) were considered and the weaker reflexes of the superstructure were ignored, even though they make up roughly 40 % of the total data set. This results in a unit cell that is four times smaller ($P\overline{1}$, a = 9.76 Å, b = 10.70 Å, c = 20.54 Å, α = 84.97 °, β = 79.45 °, γ = 67.94 °, V = 1952 Å^3, Z = 2). Thus, the superstructure was treated as a heavily disordered structure which is reflected by the very low data-to-parameter ratio for this structure. Yet, an overall considerably better model could be obtained with this treatment.

For a thorough description of the crystallographic data, both models should be considered and were therefore uploaded in the CCDC database.

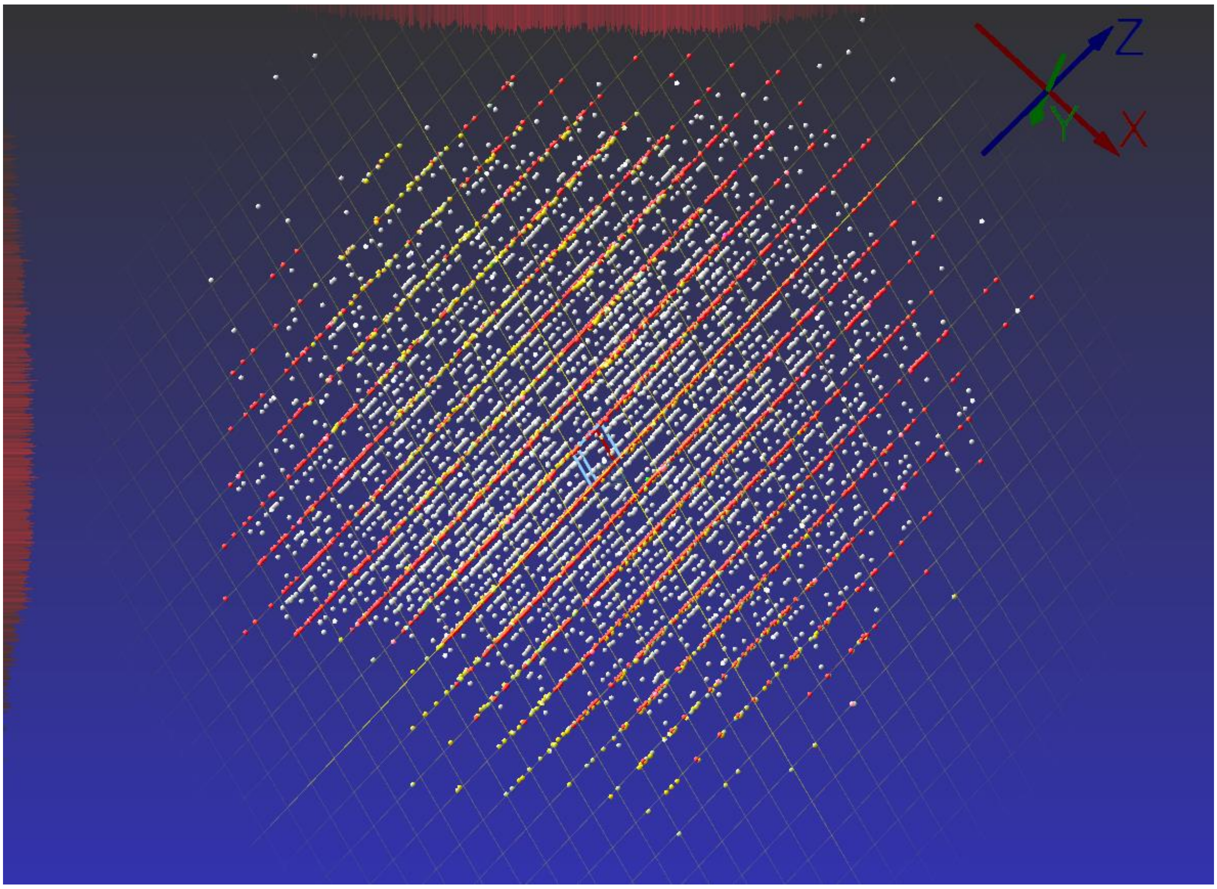

Figure S 27: Picture of the reciprocal lattice of **4** taken from Apex (total harvested reflexes: 7644). Grey reflexes (3083) stem from the superstructure. For the structure **4**$_{(1)}$, grey and red reflexes (7644) were considered, for **4**$_{(2)}$, only red reflexes (4561) were considered resulting in a division of the cell volume by factor four.

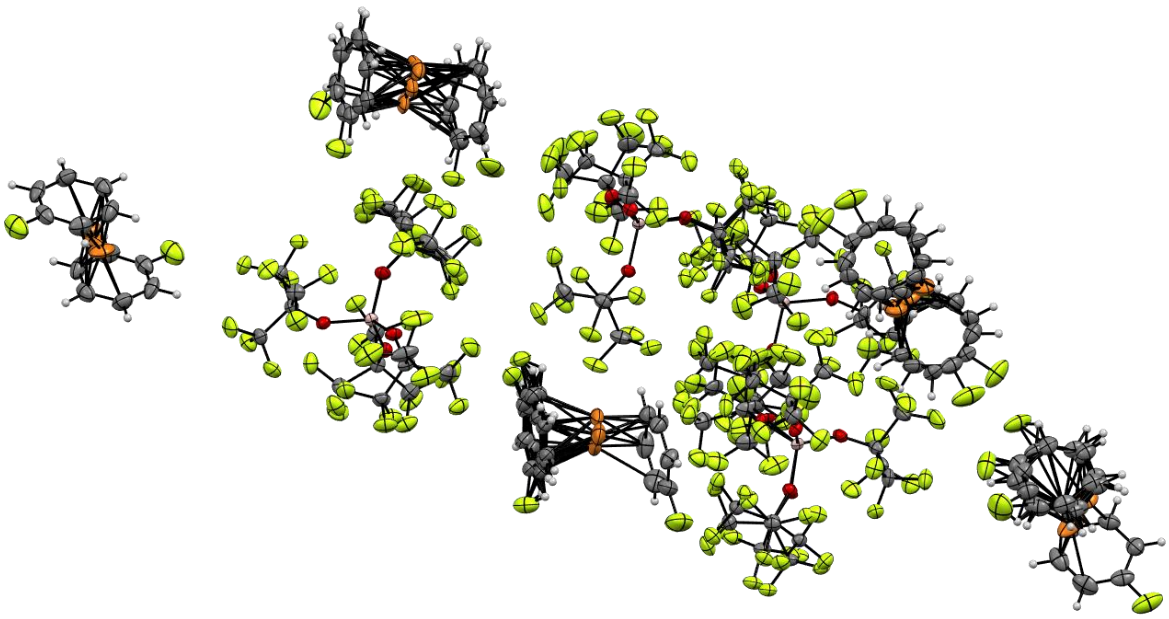

Figure S 28: Asymmetric unit of the molecular structure of [Cu(1FB)$_2$][Al(OR^F)$_4$] solved as $\mathbf{4}_{(1)}$ with disordered moieties shown. Thermal ellipsoids are set at the 50 % probability level.

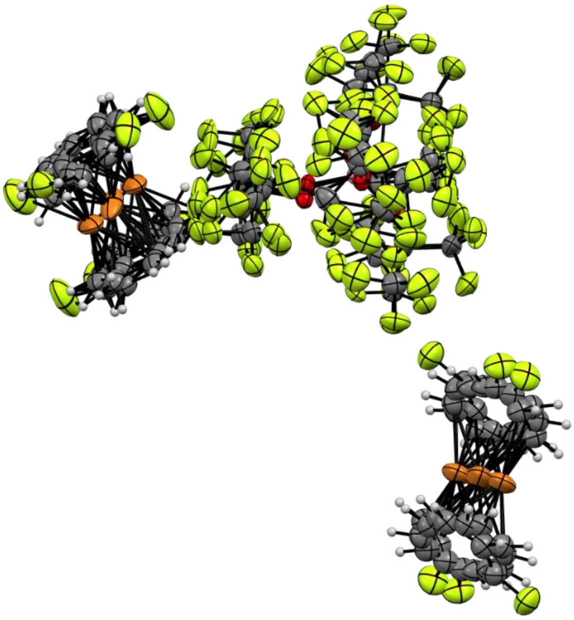

Figure S 29: Asymmetric unit of the molecular structure of [Cu(1FB)$_2$][Al(OR^F)$_4$] solved as $\mathbf{4}_{(2)}$ with disordered moieties shown. Thermal ellipsoids are set at the 50 % probability level. Only 50 % of both cationic moieties are present in the asymmetric unit, the other half is symmetry-generated by an inversion centre.

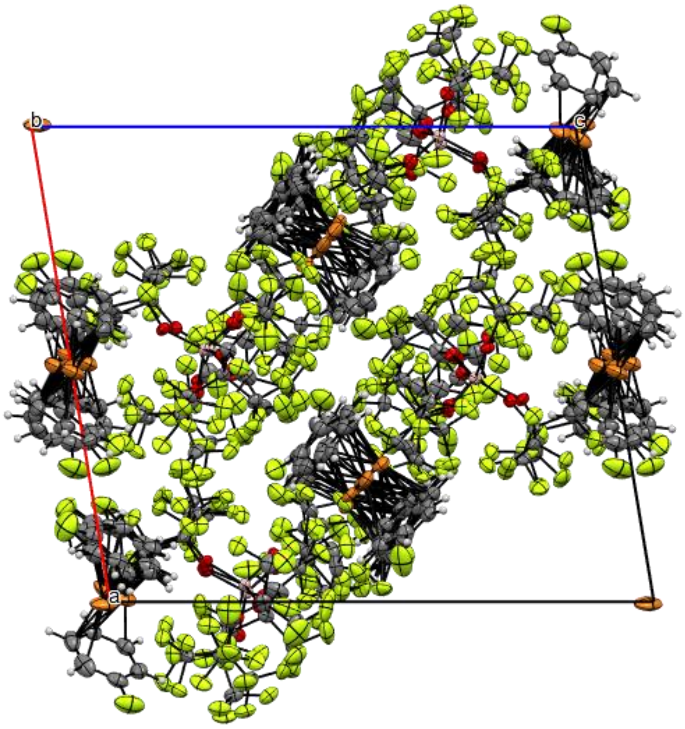

Figure S 30: Packed unit cell for the structure of $[Cu(1FB)_2][Al(OR^F)_4]$ solved as $\mathbf{4}_{(1)}$.

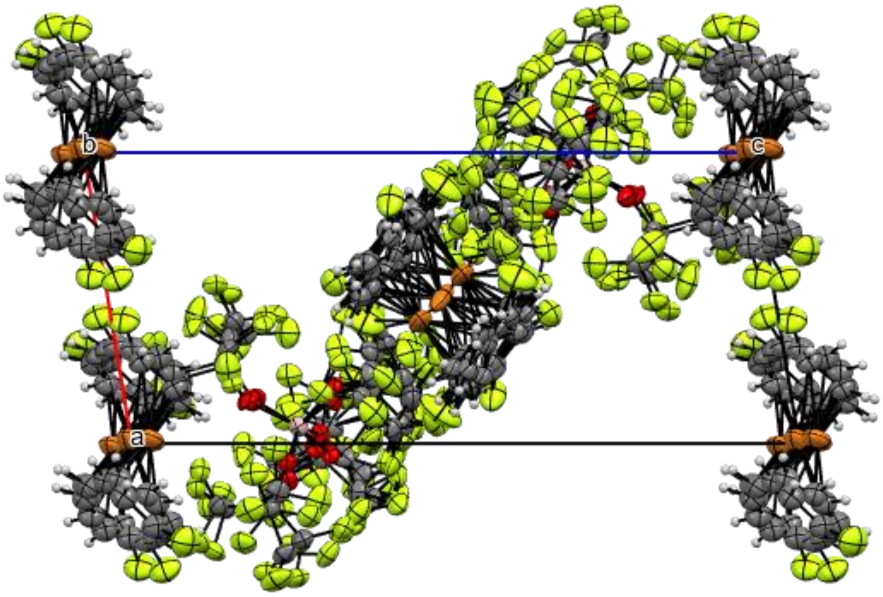

Figure S 31: Packed unit cell for the structure of $[Cu(1FB)_2][Al(OR^F)_4]$ solved as $\mathbf{4}_{(2)}$.

Details on the structure of **5**

The structure of **5** features a superstructure which is visible by rows of reflexes with alternating intensity in the reciprocal space. For the unit cell determination, only the strong reflexes were considered resulting in a smaller cell and the superstructure was treated as a disorder. Out of 4117 harvested reflexes, 3371 strong reflexes (82 %) were considered.

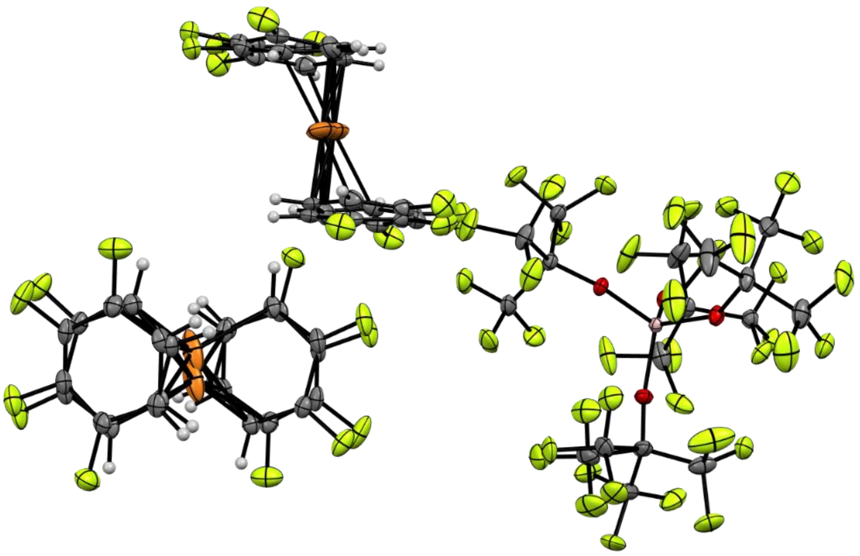

Figure S 32: Molecular structure of $[Cu(3FB)_2]^+[Al(OR^F)_4]^-$ **5** with disordered moieties shown (occupancies of disordered 3FB 50/50). Thermal ellipsoids are set at the 50 % probability level. Only 50 % of both cationic moieties are present in the asymmetric unit, the other half is symmetry-generated by an inversion centre. The crystals suffer from a superstructure which can be solved in the smaller cell, describing the superstructure as a disorder.

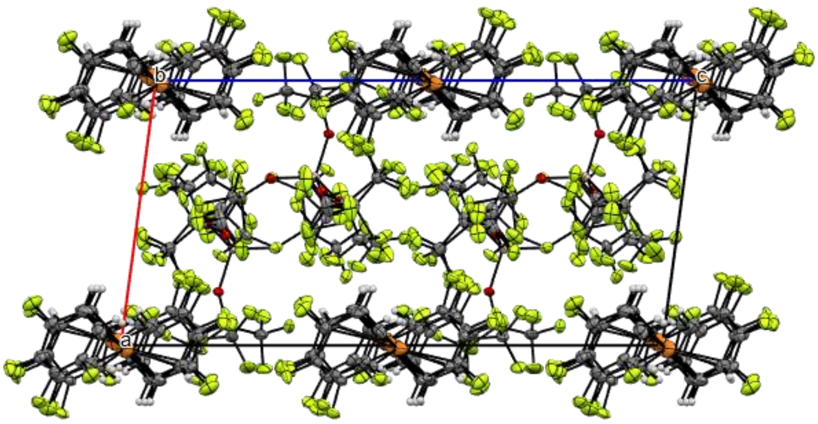

Figure S 33: Packed unit cell for the structure of $[Cu(3FB)_2]^+[Al(OR^F)_4]^-$ **5**.

Details on the structure of **6**

The structure has already been published as a picture of preliminary results in the Supporting Information of reference[2]. No publishable cif-file has been uploaded in the CCDC data base to date due the poor quality of the data in our last publication.

The first modification **6a** is obtained by layering a solution of **6** in 4FB with *n*-heptane. The structure features a superstructure and was solved and refined considering both intense and less intense reflexes resulting in a supercell.

The second modification **6b** is obtained by removing the solvent form a solution of **6** in 4FB and recrystallizing **6** in *iso*-perfluorohexane at −40 °C. This allows for obtaining a superstructure-free structure which leads to inherently considerably better *R*-values. The structure of **6b** was refined as a four-component inversion twin.

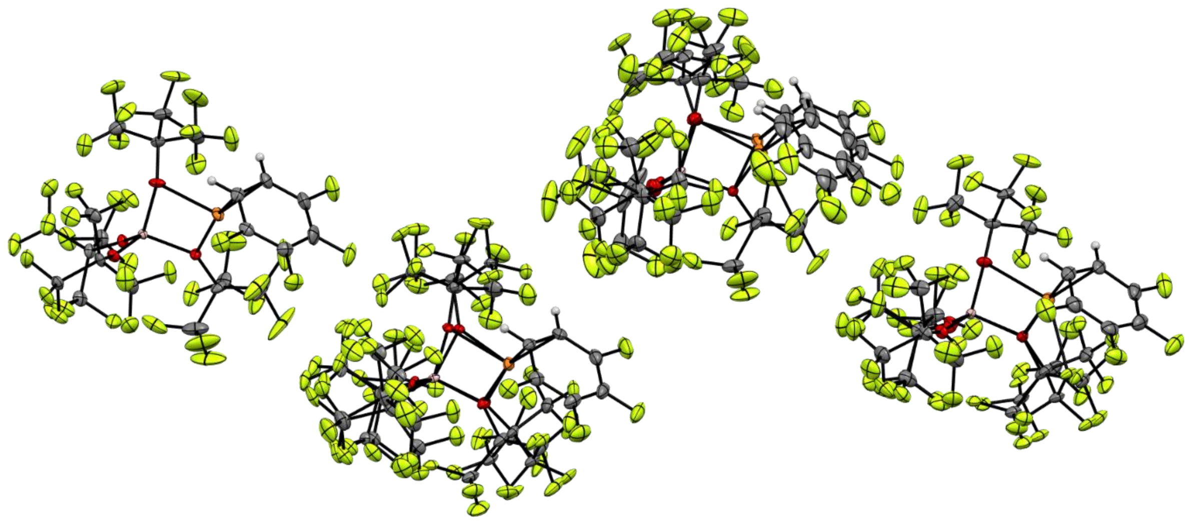

Figure S 34: Asymmetric unit of the molecular structure of [(4FB)Cu{Al(OR^F)$_4$}] **6a** with disordered moieties shown. Thermal ellipsoids are set at the 50 % probability level. The crystals suffer from a superstructure and the crystal structure was solved and refined using a monoclinic supercell (P2$_1$/*n*, a = 20.81 Å, b = 16.98 Å, c = 38.87 Å, β = 95.87 °).

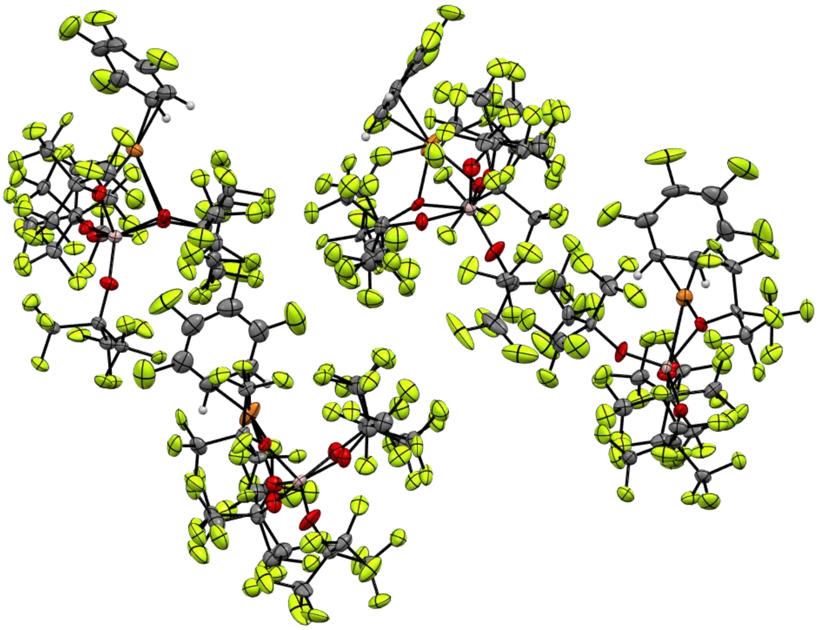

Figure S 35: Asymmetric unit of the molecular structure of [(4FB)Cu{Al(OR^F)$_4$}] **6b** with disordered moieties shown. Thermal ellipsoids are set at the 50 % probability level.

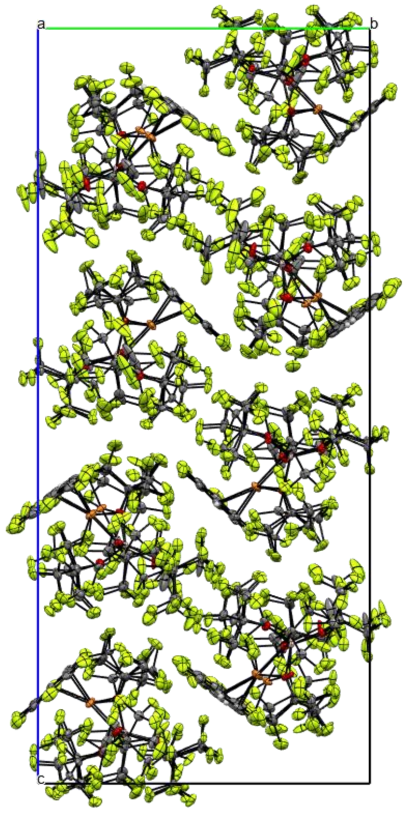

Figure S 36: Packed unit cell for the structure of [(4FB)Cu{Al(OR^F)$_4$}] **6a**.

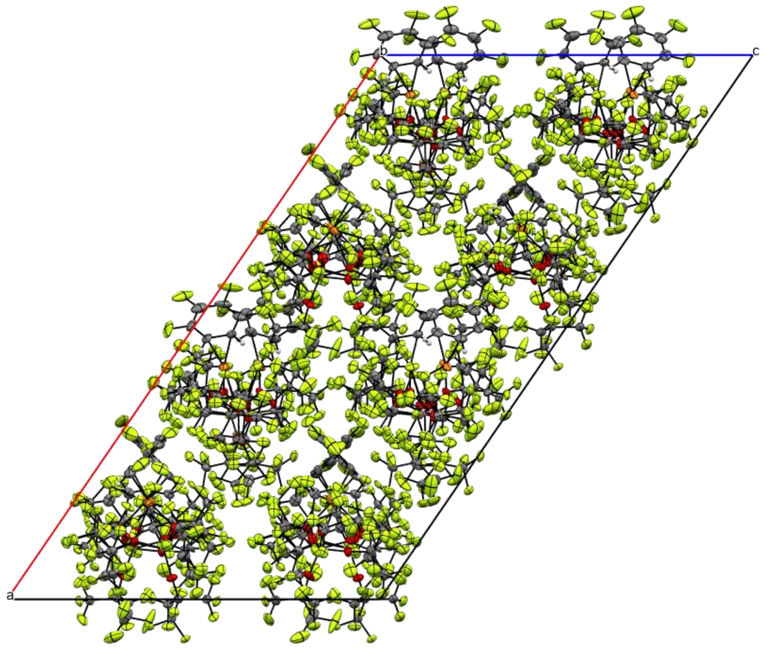

Figure S 37: Packed unit cell for the structure of [(4FB)Cu{Al(OR^F)$_4$}] **6b**.

Details on the structure of **7**

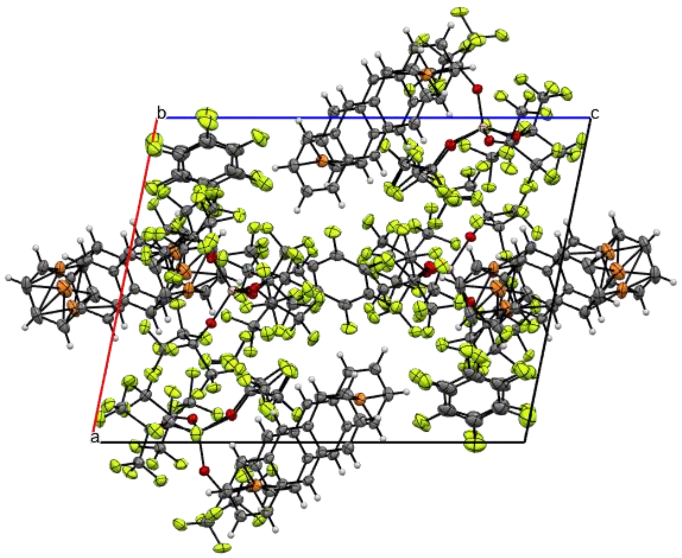

Figure S 38: Packed unit cell for the structure of $[Cu_2(anthracene)_2]^{2+}([Al(OR^F)_4]^-)_2\cdot(6FB)_{1.5}$ **7**$\cdot(6FB)_{1.5}$.

Details on the structure of **8**

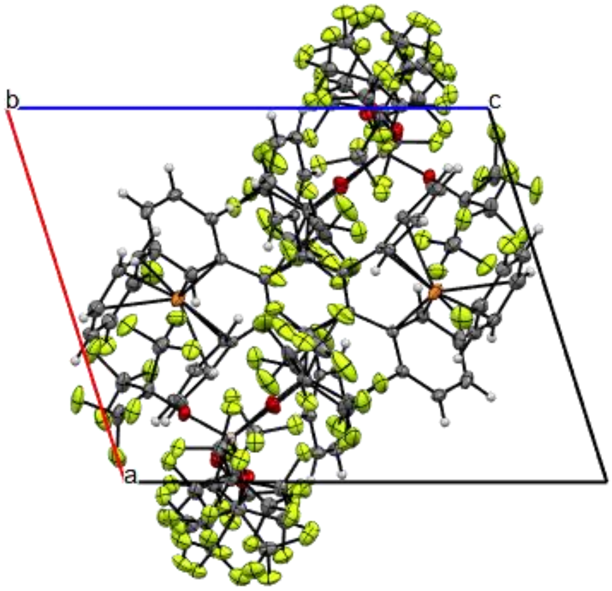

Figure S 39: Packed unit cell for the structure of $[(HPB)\{Cu(2FB)\}_2]^{2+}([Al(OR^F)_4]^-)_2$ **8**.

Details on the structure of **9**

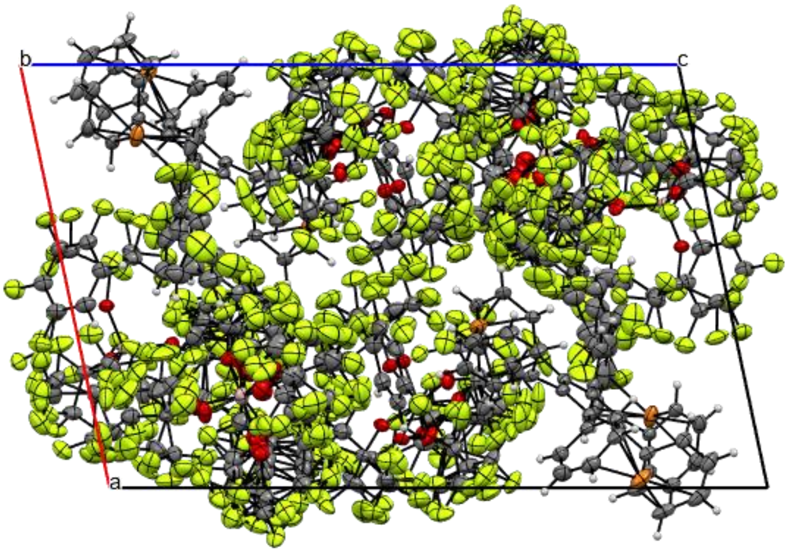

Figure S 40: Packed unit cell for the structure of $[Cu_3(HPB)]^{3+}([Al(OR^F)_4]^-)_3$ **9**.

5. Cyclic voltammetry

A similar measurement setup was used as recently published for the determination of Ag^+ potentials in different solvents.[19]

A three-electrode arrangement inside an Argon filled glovebox at RT was used for all CV measurements of Cu potentials $E°'(Cu^+/Cu, xFB)$ with a 1 mm diameter platinum disc working electrode, a platinum mesh as counter electrode and a copper wire in a compartment as a reference. The copper wire was immersed in a solution of $[(N_2)Cu\{Al(OR^F)_4\}]$ (10 mM) and $[NBu_4]^+[Al(OR^F)_4]^-$ (100 mM) as supporting electrolyte in the solvent xFB (x = 1-5), generating the Cu(I)-arene complexes *in situ*. This Cu-reference compartment was separated by a glass frit from the sample solution consisting of a solution of Fc (Fc = ferrocene, 10 mM) and $[NBu_4]^+[Al(OR^F)_4]^-$ (100 mM) in xFB (x = 1-5). Thus, the effectively measured potential is the potential of Fc^+/Fc against a copper reference. The respective Cu^+/Cu formal potentials can be obtained by taking the negative ferrocenium half-wave potentials and adding the adjusting term 0.118 V (see reference[19]).

Table S 6: Measured half-wave potentials $E_{1/2}(Fc^+/Fc,xFB)$ vs. $E(Cu^+/Cu,xFB)$ and calculated potentials $E°'(Cu^+/Cu,xFB)$ vs. $E°'(Fc^+/Fc,xFB)$ at different scan rates *v*.

Solvent	v /mV s^{-1}	$E_{1/2}((Fc^+/Fc),xFB)$ vs. $E(Cu^+(10\ mM, xFB)/Cu)$ / V	$E°'(Cu^+(xFB)/Cu)$ vs. $E°'((Fc^+/Fc),xFB)$ / V
1FB	20	−0.58	0.70
	50	−0.58	0.69
	100	−0.58	0.70
	200	−0.58	0.70
	500	−0.58	0.70
	1000	−0.57	0.69
2FB	20	−0.81	0.93
	50	−0.81	0.93
	100	−0.81	0.93
	200	−0.81	0.93
	500	−0.81	0.93
	1000	−0.81	0.93
3FB	20	−1.06	1.18
	50	−1.06	1.17
	100	−1.06	1.17
	200	−1.06	1.18
	500	−1.05	1.17
	1000	−1.05	1.17
4FB	20	−1.26	1.38
	50	−1.26	1.38
	100	−1.26	1.38
	200	−1.26	1.38
	500	−1.25	1.37
	1000	−1.25	1.37
5FB	20	−1.41	1.52
	50	−1.41	1.53
	100	−1.43	1.55
	200	−1.50	1.61
	500	−1.52	1.64
	1000	−1.53	1.65

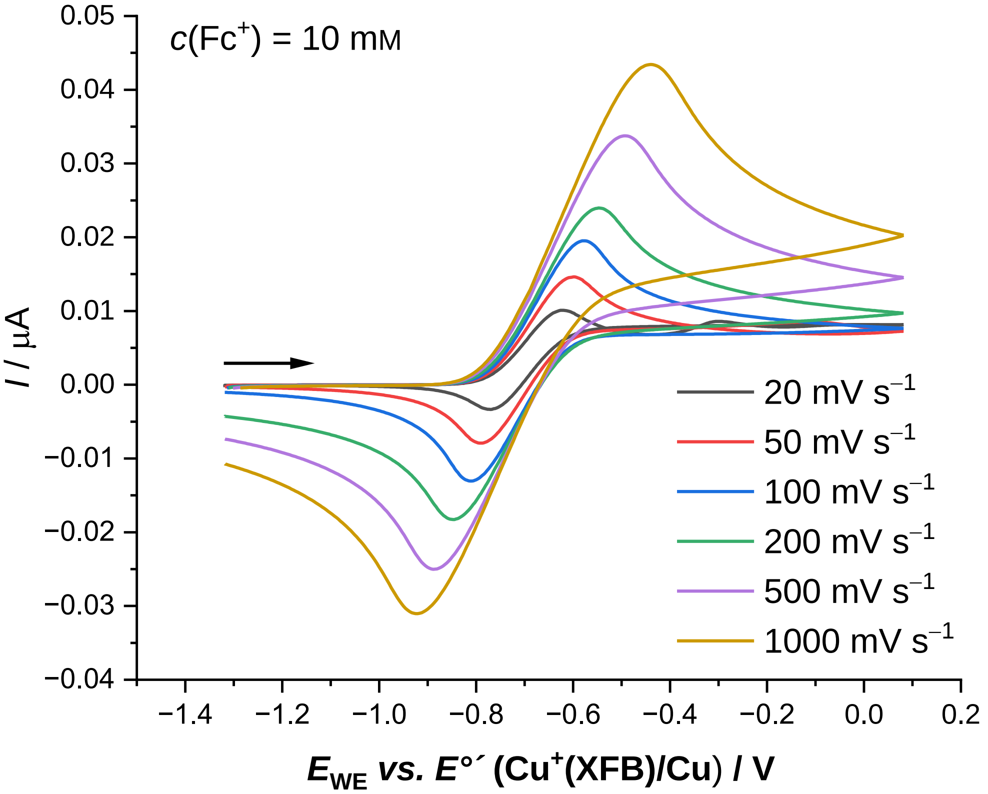

Figure S 41: 1st cycles of Fc^+/Fc (10 mM) in 1FB vs. *E*(Cu^+(10 mM, 1FB)/Cu) measured at six different scan rates (20, 50, 100, 200, 500 & 1000 mV s^{-1}). The black arrow indicates for all scan rates the start and the direction of the 1st cycle.

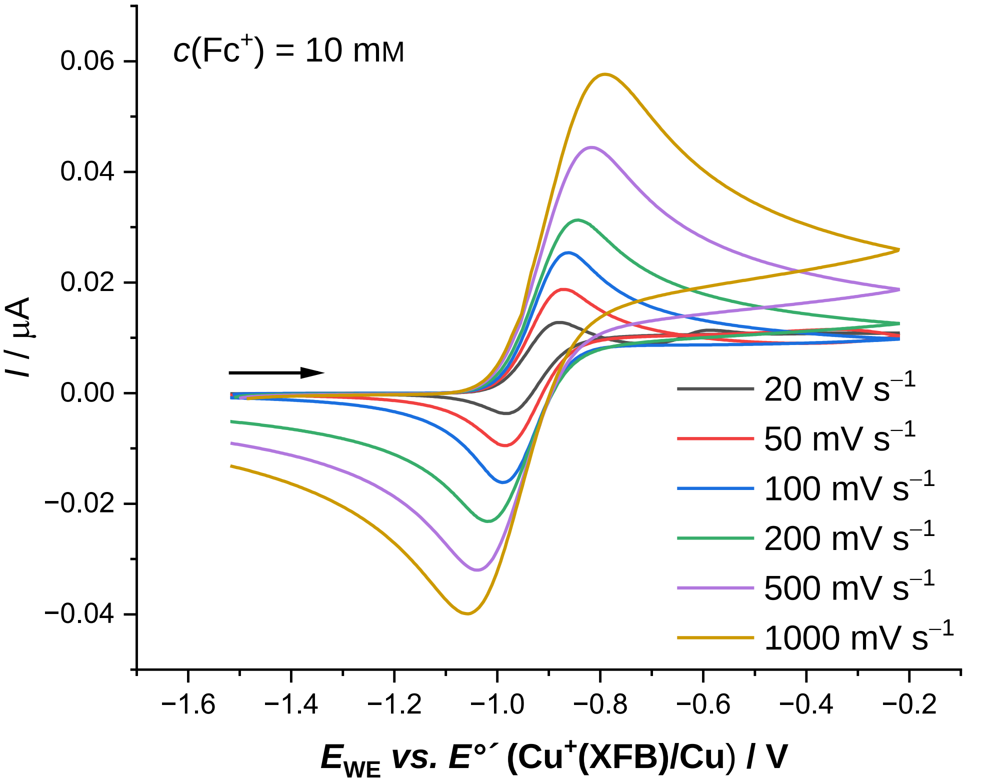

Figure S 42: 1st cycles of Fc^+/Fc (10 mM) in 2FB vs. *E*(Cu^+(10 mM, 1FB)/Cu) measured at six different scan rates (20, 50, 100, 200, 500 & 1000 mV s^{-1}). The black arrow indicates for all scan rates the start and the direction of the 1st cycle.

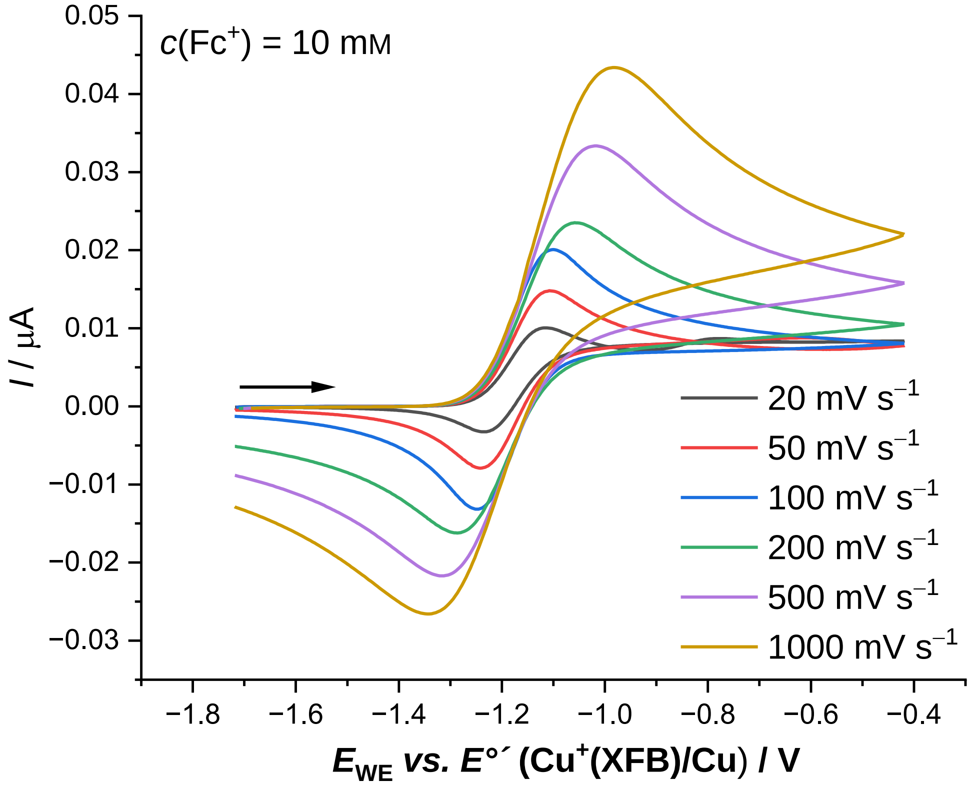

Figure S 43: 1st cycles of Fc^+/Fc (10 mM) in 3FB vs. *E*(Cu^+(10 mM, 1FB)/Cu) measured at six different scan rates (20, 50, 100, 200, 500 & 1000 mV s^{-1}). The black arrow indicates for all scan rates the start and the direction of the 1st cycle.

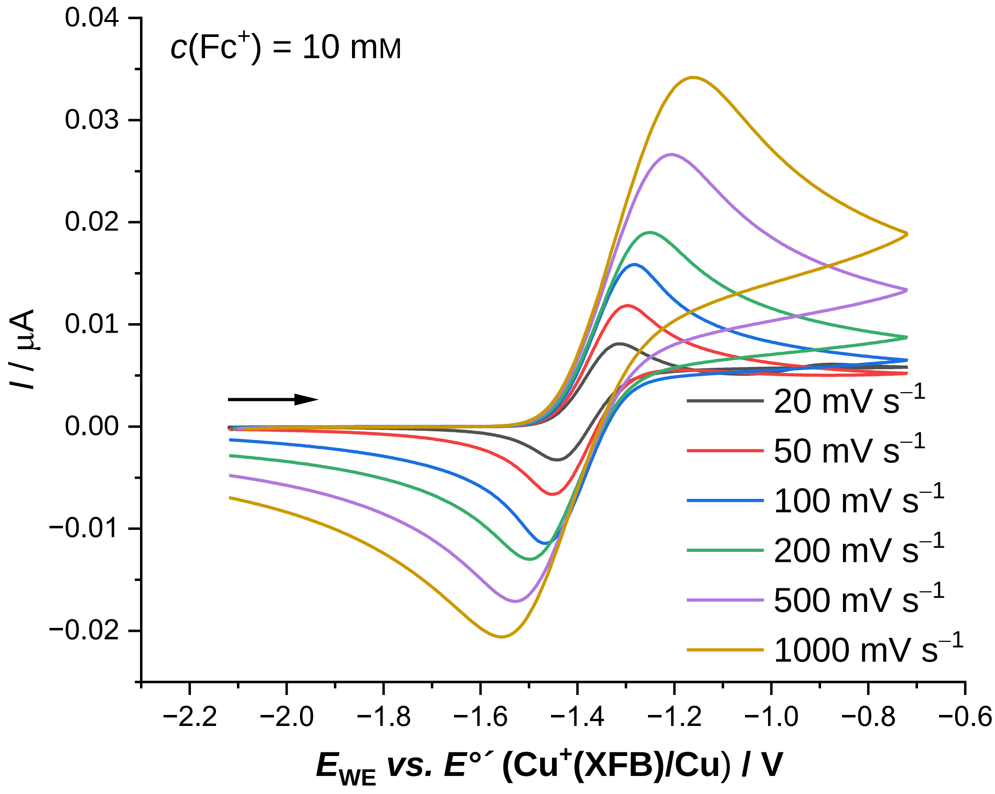

Figure S 44: 1st cycles of Fc^+/Fc (10 mM) in 4FB vs. *E*(Cu^+(10 mM, 1FB)/Cu) measured at six different scan rates (20, 50, 100, 200, 500 & 1000 mV s^{-1}). The black arrow indicates for all scan rates the start and the direction of the 1st cycle.

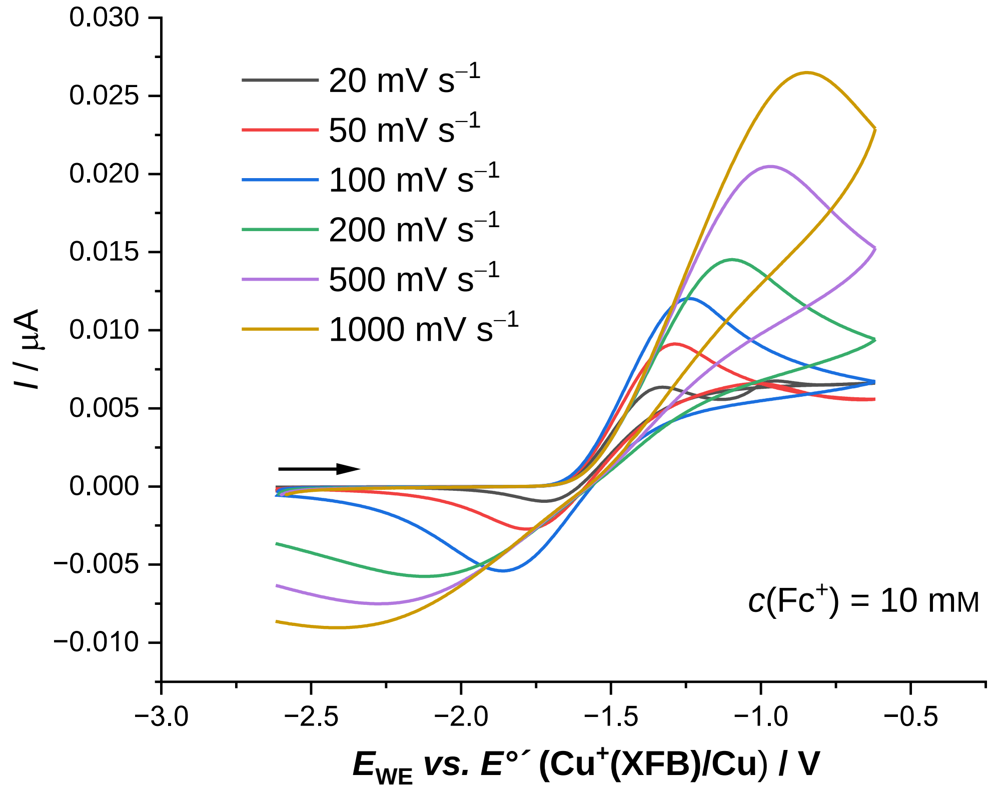

Figure S 45: 1st cycles of Fc^+/Fc (10 mM) in 5FB vs. *E*(Cu^+(10 mM, 1FB)/Cu) measured at six different scan rates (20, 50, 100, 200, 500 & 1000 mV s^{-1}). The black arrow indicates for all scan rates the start and the direction of the 1st cycle. The half-wave potential is not stable over the measured scan rates but increases with increasing scan rate.

Table S 7: Anodic peak current I_{pa} obtained by cyclic voltammetry of $[(N_2)Cu\{Al(OR^F)_4\}]$ in fluorinated arenes xFB at different scan rates *v*, together with $v^{1/2}$ and R^2 of the linear regression.

Solvent	$v^{1/2}$ / (mV s^{-1})$^{1/2}$	I_{pa} / µA	R^2
1FB	4.47	10.04	0.98721
	7.07	14.58	
	10.0	19.48	
	14.1	22.89	
	22.4	31.47	
	31.6	39.96	
2FB	4.47	12.71	0.98840
	7.07	18.71	
	10.0	25.14	
	14.1	29.90	
	22.4	41.19	
	31.6	52.82	
3FB	4.47	10.03	0.97952
	7.07	14.80	
	10.0	19.93	
	14.1	21.92	
	22.4	29.91	
	31.6	38.10	
4FB	4.47	8.12	0.98601
	7.07	11.87	
	10.0	15.70	
	14.1	18.21	
	22.4	24.95	
	31.6	31.64	
5FB	4.47	6.32	0.98432
	7.07	9.13	
	10.0	11.98	
	14.1	13.59	
	22.4	18.32	
	31.6	23.36	

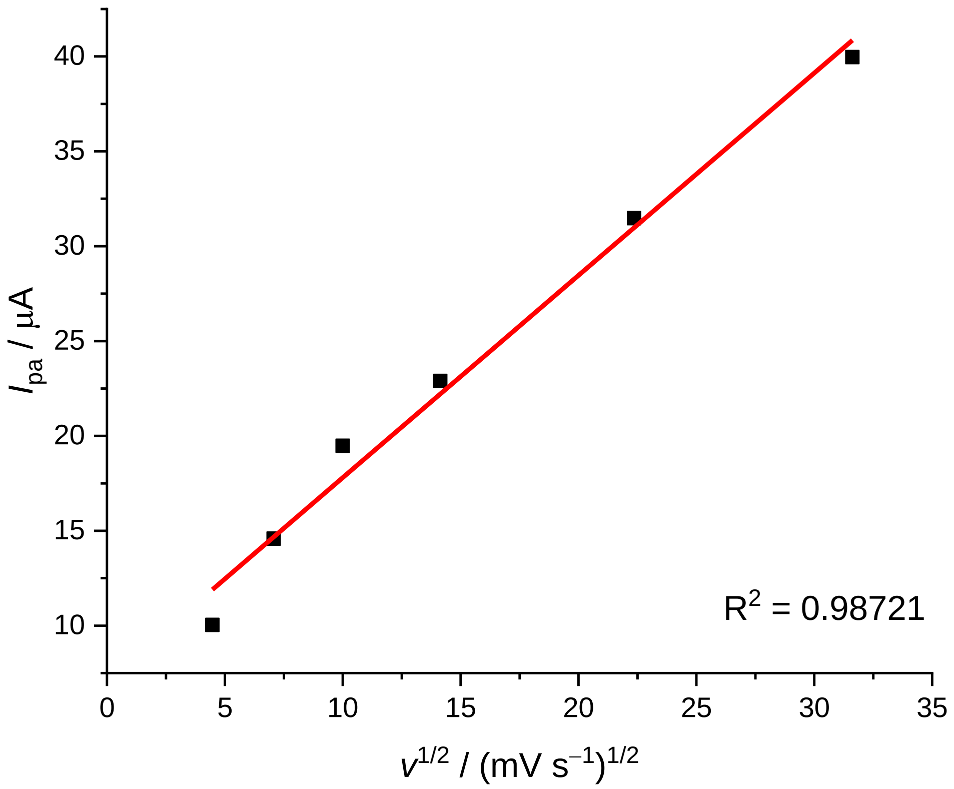

Figure S 46: Plot of the anodic peak current I_{pa} vs. the square root of the scan rate $v^{1/2}$ from the cyclic voltammograms of Fc^+/Fc vs. $E(Cu^+(1FB)/Cu)$ in 1FB, together with the linear regression and corresponding R^2 value for the linear regression.

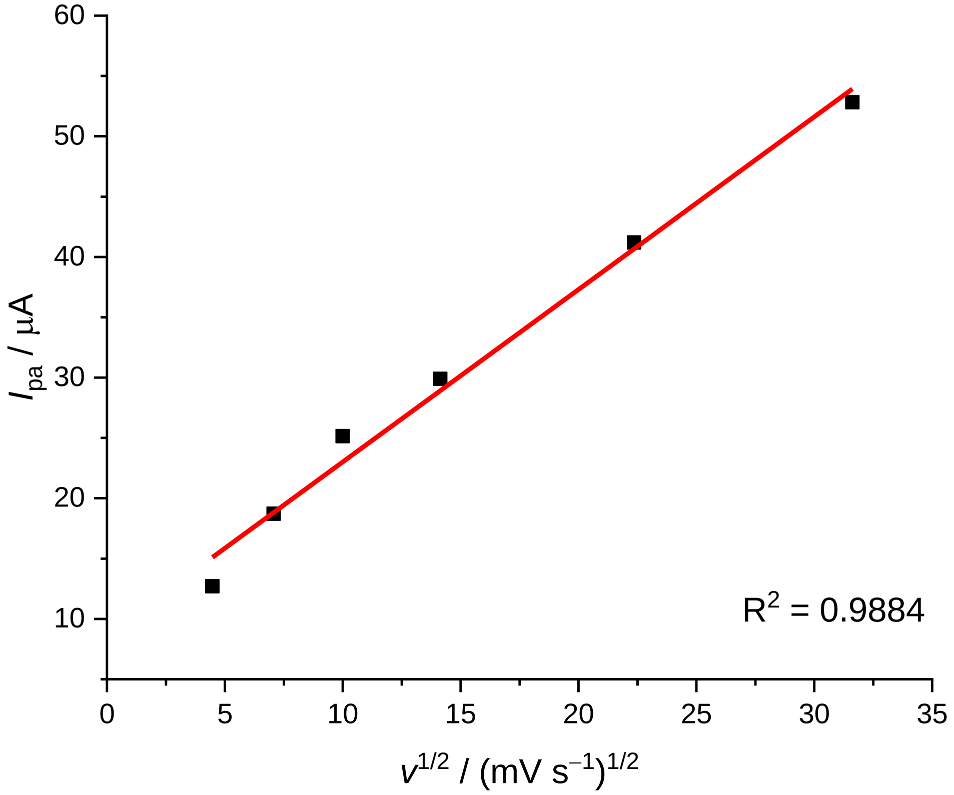

Figure S 47: Plot of the anodic peak current I_{pa} vs. the square root of the scan rate $v^{1/2}$ from the cyclic voltammograms of Fc^+/Fc vs. $E(Cu^+(2FB)/Cu)$ in 2FB, together with the linear regression and corresponding R^2 value for the linear regression.

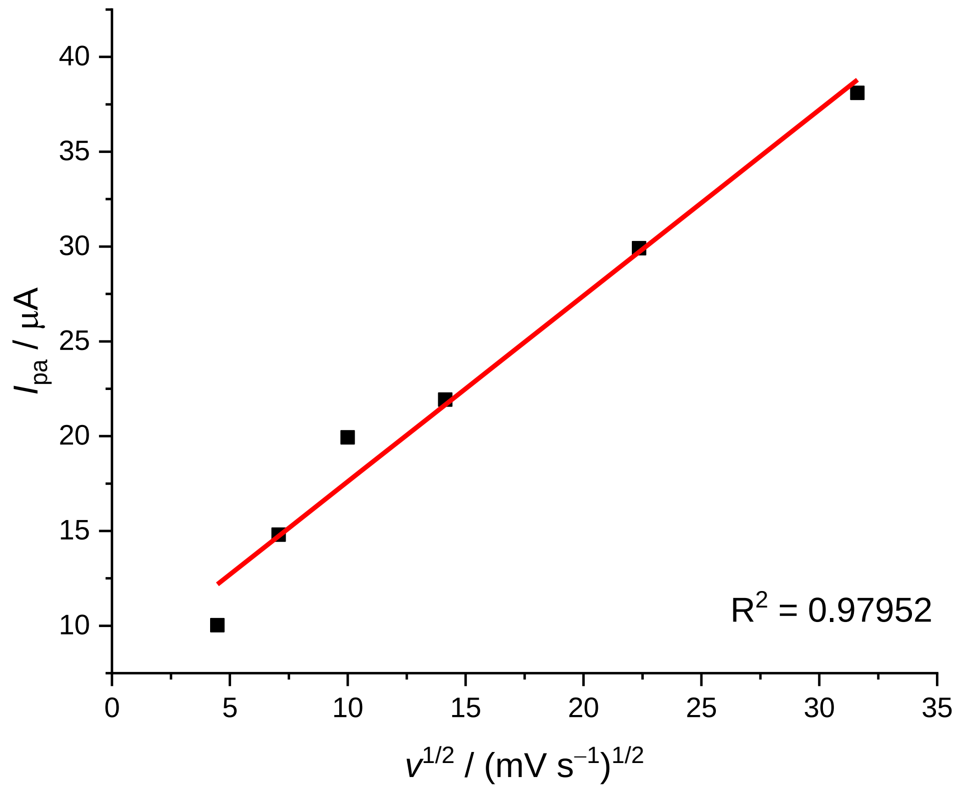

Figure S 48: Plot of the anodic peak current I_{pa} vs. the square root of the scan rate $v^{1/2}$ from the cyclic voltammograms of Fc^+/Fc vs. E(Cu^+(3FB)/Cu) in 3FB, together with the linear regression and corresponding R^2 value for the linear regression.

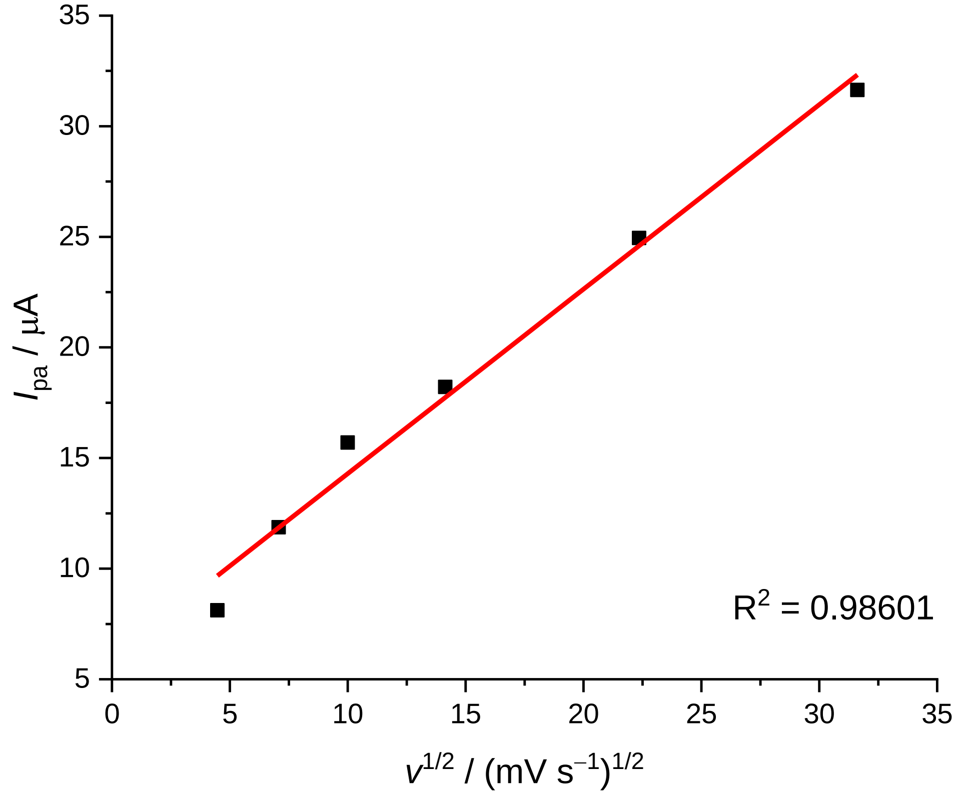

Figure S 49: Plot of the anodic peak current I_{pa} vs. the square root of the scan rate $v^{1/2}$ from the cyclic voltammograms of Fc^+/Fc vs. E(Cu^+(4FB)/Cu) in 4FB, together with the linear regression and corresponding R^2 value for the linear regression.

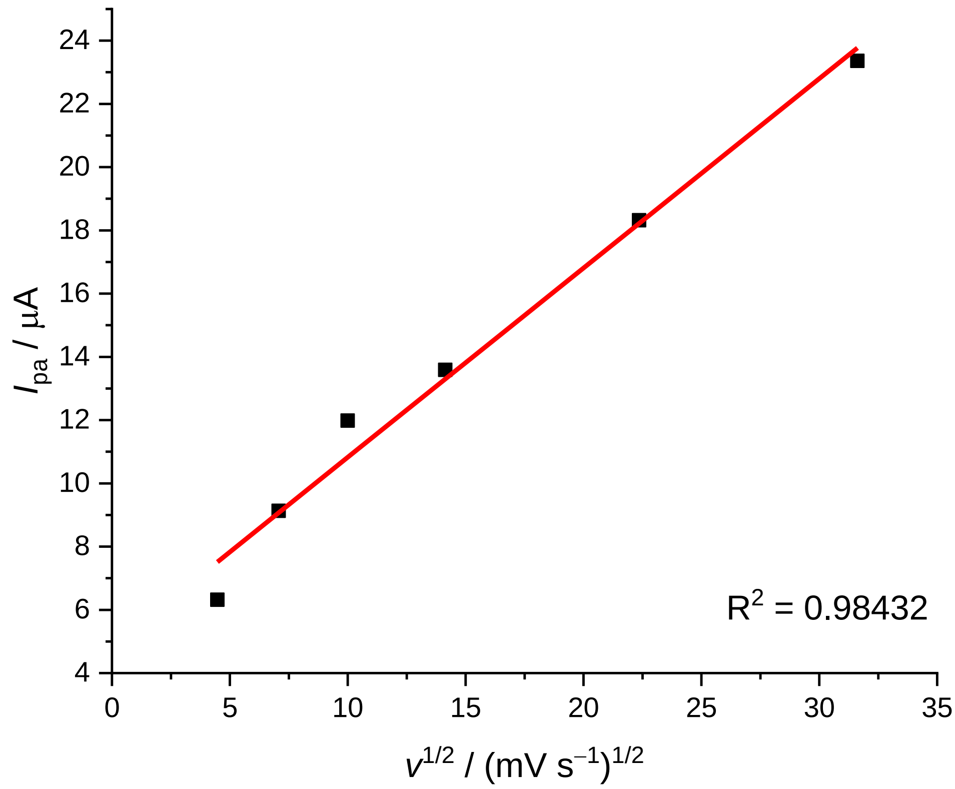

Figure S 50: Plot of the anodic peak current I_{pa} vs. the square root of the scan rate $v^{1/2}$ from the cyclic voltammograms of Fc^+/Fc vs. E(Cu^+(5FB)/Cu) in 5FB, together with the linear regression and corresponding R^2 value for the linear regression.

6. Computational details

General Information

Gibbs free energies $G°$ for molecular structures were computed at T = 298.15 K and p = 1.00 atm using DFT at the RI-r^{2}SCAN-3c(D4)/def2-mTZVPP level of theory using the ORCA program (version 5.0).[13] The Gibbs free energy can be calculated using Eq. S1.

$$G° = H° - T \cdot S° \tag{S1}$$

While $G°$ can directly be extracted from the ORCA output-file for polyatomic structures, it has to be calculated for monoatomic structures using Eq. S1 and Eq. S2, as well as the Sackur-Tetrode equation for the determination of the standard entropy $S°$.[20]

$$H° = E_{el} + E_{vrt} + R \cdot T \tag{S2}$$

In Eq. S2, E_{el} is the electronic energy (found as 'final single point energy' in the ORCA output-file), E_{vrt} is the sum of translational, rotational and vibrational energies including zero-point energy (for monoatomic structures $E_{vrt} = \frac{3}{2}RT$ resulting from the translational contribution) and R is the ideal gas constant.

Gibbs free energies of solvation $G°_{(solv)}$ were computed using the CPCM module [18] at the respective level of theory.

Table S 8: Summary of computed thermodynamic data for monoatomic structures.

Atom$_{(solv)}$	E_{el} / kJ mol^{-1}	$H°$ / kJ mol^{-1}	$S°$ / kJ mol^{-1} K^{-1}	$G°$ / kJ mol^{-1}
$Cu^+_{(gas)}$	−4306265.17	−4306258.97	0.1606	−4306306.86
$Cu^+_{(3FB)}$	−4306665.61	−4306659.41	0.1606	−4306707.31
$Cu^+_{(2FB)}$	−4306655.05	−4306648.85	0.1606	−4306696.75
$Cu^+_{(1FB)}$	−4306612.27	−4306606.08	0.1606	−4306653.97
$Ag^+_{(gas)}$	−385230.739	−385224.542	0.1672	−385274.403
$Ag^+_{(3FB)}$	−385555.253	−385549.055	0.1672	−385598.916
$Ag^+_{(2FB)}$	−385546.696	−385540.498	0.1672	−385590.359
$Ag^+_{(1FB)}$	−385512.027	−385505.830	0.1672	−385555.691

Table S 9: Summary of computed thermodynamic data for polyatomic structures.

Molecule$_{(solv)}$	$G°$ / kJ mol^{-1}	Molecule$_{(solv)}$	$G°$ / kJ mol^{-1}
$[Cu(C_6H_6)_3]^+_{(C6H6)}$	−6135029.42	$[Cu(3FB)_2]^+_{(3FB)}$	−7089138.76
$[Cu(C_6H_6)_2]^+_{(C6H6)}$	−5525616.69	$3FB_{(3FB)}$	−1391161.48
$C_6H_{6(C6H6)}$	−609413.26	$[Ag(1FB)_3]^+_{(1FB)}$	−2995705.55
$[Cu(1FB)_3]^+_{(1FB)}$	−6916847.39	$[Ag(2FB)_2]^+_{(2FB)}$	−2646857.48
$[Cu(1FB)_2]^+_{(1FB)}$	−6046844.79	$[Ag(3FB)_2]^+_{(3FB)}$	−3167989.44
$1FB_{(1FB)}$	−870012.72	$[(4FB)Ag\{Al(OR^F)_4\}]_{(gas)}$	−14502427.72
$[Cu(2FB)_3]^+_{(2FB)}$	−7698577.02	$[Ag\{Al(OR^F)_4\}]_{(gas)}$	−12850560.93
$[Cu(2FB)_2]^+_{(2FB)}$	−6568007.84	$[(4FB)Cu\{Al(OR^F)_4\}]_{(gas)}$	−18423551.87
$2FB_{(2FB)}$	−1130589.47	$[Cu\{Al(OR^F)_4\}]_{(gas)}$	−16771679.88
$[Cu(3FB)_3]^+_{(3FB)}$	−8480276.81		

Coordinates of computed structures at the RI-r²SCAN-3c(D4)/def2-mTZVPP level of theory in Angstrom

$[Cu(C_6H_6)_3]^+_{(C6H6)}$

H	3.531719	-1.232655	0.952810
C	2.885249	-2.026294	1.316084
C	3.416460	-3.262836	1.660712
H	4.485899	-3.433907	1.579694
C	2.577131	-4.293279	2.094806
H	2.997725	-5.262788	2.345423
C	1.207862	-4.082588	2.208996
H	0.557851	-4.883806	2.547773
C	0.657359	-2.843286	1.857658
H	-0.421917	-2.708746	1.851914
C	1.497313	-1.809699	1.390761
H	1.067512	-0.906437	0.961547
H	4.318806	-1.087265	4.909717
C	3.335962	-1.179768	5.362700
H	3.623071	0.464791	6.713847
H	2.844798	-2.986696	4.277511
C	2.946626	-0.316007	6.378715
C	2.475317	-2.211518	4.946366
C	1.694324	-0.459655	6.983511
C	1.200912	-2.334757	5.539851
C	0.818512	-1.451604	6.557684
H	1.404256	0.208415	7.789252
H	0.558195	-3.171288	5.275104
H	-0.154333	-1.560506	7.027739
Cu	1.212545	-1.229258	3.468046
H	2.408821	1.951538	3.148988
H	1.490951	2.265409	0.866729
C	1.442137	1.532443	2.885055
C	0.922275	1.713493	1.609383
C	0.695690	0.844943	3.859306
C	-0.338001	1.203314	1.283935
H	1.023804	0.853175	4.897082
H	-0.745418	1.364807	0.290154
C	-0.562418	0.304592	3.517067
C	-1.070737	0.488747	2.224731
H	-1.175372	-0.167504	4.281475
H	-2.048408	0.091290	1.968906

$[Cu(C_6H_6)_2]^+_{(C6H6)}$

H	1.522460	-3.285508	0.515267
C	0.672415	-2.777372	0.959990
C	-0.469528	-3.504578	1.307483
H	-0.503371	-4.576075	1.136477
C	-1.568776	-2.860426	1.870847
H	-2.460632	-3.422530	2.130596
C	-1.548082	-1.463016	2.066660
H	-2.442884	-0.943024	2.400877
C	-0.380342	-0.732371	1.732644
H	-0.371410	0.350307	1.826352
C	0.727807	-1.404873	1.184413
H	1.616660	-0.840989	0.918769
H	1.141628	0.775965	5.671959
C	0.557209	-0.133998	5.767146
H	-1.125478	0.847751	6.672384
H	2.135702	-1.402177	4.991405
C	-0.715886	-0.096696	6.327669
C	1.108724	-1.358272	5.344457
C	-1.470010	-1.267692	6.446839
C	0.353735	-2.550385	5.480271
C	-0.950616	-2.485492	6.014868
H	-2.464633	-1.230492	6.880224
H	0.803059	-3.512900	5.246906
H	-1.531237	-3.397229	6.116914
Cu	-0.244855	-1.650510	3.689024

$C_6H_{6(C6H6)}$

H	1.216519	-0.164862	2.154647
C	0.683500	-0.092480	1.210583
C	-0.708560	-0.035142	1.199140
H	-1.261236	-0.062811	2.134238
C	-1.392039	0.057249	-0.011431
H	-2.477647	0.101939	-0.020444
C	-0.683481	0.092483	-1.210555
H	-1.216499	0.164865	-2.154619
C	0.708587	0.035144	-1.199108
H	1.261150	0.062821	-2.134272
C	1.392058	-0.057247	0.011468
H	2.477668	-0.101929	0.020352

$[Cu(1FB)_3]^+_{(1FB)}$

H	3.189946	-1.019039	0.841642
C	2.692394	-1.900398	1.235235
C	3.430285	-3.041202	1.518903
H	4.505365	-3.074960	1.373996
C	2.756238	-4.166988	1.981276
F	3.471732	-5.279566	2.234864
C	1.390880	-4.192149	2.206746
H	0.915186	-5.093117	2.580364
C	0.654484	-3.038199	1.926439
H	-0.427221	-3.053729	2.031240
C	1.295501	-1.887671	1.421490
H	0.703902	-1.064754	1.026072
H	4.224513	-1.086680	5.179640

C	3.212941	-1.238500	5.543575
H	3.313203	0.359142	7.001073
H	2.879140	-2.998470	4.332113
C	2.718468	-0.435105	6.561059
C	2.424442	-2.270474	5.000987
C	1.427266	-0.671010	7.023027
C	1.113628	-2.470360	5.485253
C	0.606063	-1.656090	6.503175
F	0.952269	0.105853	8.015153
H	0.521642	-3.309641	5.129346
H	-0.395448	-1.797515	6.896207
Cu	1.250925	-1.261220	3.475749
H	2.696188	1.560912	3.336791
H	2.059367	2.091014	0.978649
C	1.692083	1.349166	2.980984
C	1.344699	1.658570	1.671592
C	0.740095	0.798915	3.862044
C	0.038438	1.419546	1.263178
H	0.959606	0.744813	4.926593
F	-0.305427	1.738591	0.000881
C	-0.567025	0.546474	3.399023
C	-0.923154	0.855498	2.087822
H	-1.316027	0.154720	4.081457
H	-1.926587	0.680034	1.713635

$[Cu(1FB)_2]^+_{(1FB)}$

H	2.997172	-2.971983	0.970245
C	1.967462	-2.664215	1.120484
C	0.942133	-3.599965	1.025511
F	1.263557	-4.873894	0.742726
C	-0.392043	-3.282064	1.220191
H	-1.155784	-4.048503	1.138478
C	-0.726870	-1.944186	1.491322
H	-1.772871	-1.654724	1.550966
C	0.291275	-0.962801	1.585447
H	0.031616	0.086741	1.699928
C	1.638466	-1.346891	1.415860
H	2.421830	-0.599200	1.490774
H	1.679164	0.905739	5.953207
C	1.065778	0.020485	5.820285
F	-0.859724	1.307950	6.215662
H	2.705744	-1.310459	5.436534
C	-0.317944	0.110489	5.935040
C	1.628174	-1.215995	5.527294
C	-1.163120	-0.973508	5.762701
C	0.814441	-2.359578	5.379826
C	-0.592091	-2.229712	5.494318
H	-2.237006	-0.851872	5.859355
H	1.266392	-3.341805	5.266182
H	-1.228031	-3.110100	5.451566
Cu	0.087832	-1.823087	3.487088

$1FB_{(1FB)}$

F	1.322118	-0.179250	2.341865
C	0.655848	-0.088721	1.161587
C	-0.727926	-0.033667	1.185990
H	-1.256909	-0.062890	2.133432
C	-1.402203	0.058626	-0.029859
H	-2.487468	0.103097	-0.034401
C	-0.694379	0.093940	-1.229877
H	-1.226969	0.166236	-2.173182
C	0.698064	0.036594	-1.217317
H	1.254226	0.063944	-2.149902
C	1.390815	-0.055877	-0.011885
H	2.474784	-0.102004	0.023559

$[Cu(2FB)_3]^+_{(2FB)}$

H	4.030440	-1.156265	1.230217
C	3.277178	-1.921935	1.387688
C	3.665772	-3.220385	1.641485
F	4.964683	-3.538838	1.719630
C	2.717236	-4.231228	1.822777
F	3.147478	-5.477060	2.061779
C	1.365997	-3.955134	1.773189
H	0.649821	-4.756860	1.923255
C	0.950941	-2.643183	1.509624
H	-0.106744	-2.439957	1.367407
C	1.905147	-1.625460	1.309826
H	1.600458	-0.649250	0.940371
H	4.331908	-1.432546	4.793243
C	3.352088	-1.471437	5.259498
F	3.939604	0.311097	6.691290
H	2.686546	-3.226845	4.196455
C	3.047605	-0.602963	6.286479
C	2.389632	-2.419495	4.861933
C	1.809447	-0.662195	6.932959
C	1.128303	-2.448077	5.490027
C	0.841854	-1.563375	6.536452
F	1.571402	0.192459	7.936662
H	0.404981	-3.213484	5.223035
H	-0.116705	-1.576801	7.045587
Cu	1.256961	-1.299298	3.394657
H	2.263430	2.133885	3.371427
F	1.558754	2.648257	0.915431
C	1.364510	1.627150	3.034415
C	0.898641	1.839500	1.754613
C	0.642813	0.774364	3.887331
C	-0.269506	1.217015	1.301959

H	0.912601	0.735078	4.939538
F	-0.679917	1.460702	0.050465
C	-0.525631	0.134427	3.425469
C	-0.979277	0.360395	2.117237
H	-1.137433	-0.450192	4.108183
H	-1.881360	-0.111925	1.740978

$[Cu(2FB)_2]^+_{(2FB)}$

Cu	7.111971	0.028933	7.690940
C	9.035839	0.705574	7.118109
H	9.706798	-0.021816	7.568744
C	8.311193	0.380244	5.946111
H	8.411943	-0.603035	5.493716
C	7.549940	1.368514	5.293502
H	6.987627	1.142646	4.392861
C	8.986769	2.016876	7.632358
H	9.535157	2.291734	8.527821
C	7.541560	2.652074	5.799865
C	8.251486	2.972908	6.961515
F	8.196591	4.229367	7.417856
F	6.837655	3.615246	5.194166
C	5.081948	-0.170480	8.322482
H	4.759843	0.841190	8.555270
C	5.936852	-0.870069	9.208247
H	6.274030	-0.403116	10.130283
C	6.260016	-2.217635	8.954062
H	6.912957	-2.775485	9.617799
C	4.552633	-0.825416	7.193706
H	3.893994	-0.309827	6.501896
C	5.707826	-2.844366	7.854761
C	4.859246	-2.154780	6.983196
F	4.359217	-2.806508	5.927339
F	5.985438	-4.127094	7.595806

$2FB_{(2FB)}$

F	1.322046	-0.179977	2.337418
C	0.682200	-0.089165	1.152483
C	-0.705808	-0.031919	1.141035
F	-1.371039	-0.067628	2.314884
C	-1.399197	0.059847	-0.052360
H	-2.484018	0.102895	-0.032618
C	-0.682888	0.094349	-1.247547
H	-1.219614	0.166101	-2.188147
C	0.708764	0.037351	-1.236085
H	1.264855	0.064487	-2.167751
C	1.400247	-0.055010	-0.029337
H	2.484461	-0.101310	0.008035

$[Cu(3FB)_3]^+_{(3FB)}$

H	3.408438	-1.123325	0.841464
C	2.819002	-1.948704	1.226935
C	3.434993	-3.141326	1.549891
F	4.757960	-3.282030	1.424212
C	2.705103	-4.238505	2.013265
F	3.324137	-5.380142	2.308282
C	1.334104	-4.114749	2.184236
F	0.652233	-5.165266	2.647752
C	0.671960	-2.928435	1.873707
H	-0.414188	-2.898274	1.911921
C	1.426026	-1.846699	1.372435
H	0.913468	-0.990815	0.940778
H	4.311449	-0.948348	4.987127
C	3.328635	-1.128441	5.409871
F	3.614141	0.669445	6.913558
H	2.940888	-2.911039	4.271744
C	2.870982	-0.337241	6.444420
C	2.515763	-2.175812	4.950316
C	1.624242	-0.555327	7.035400
C	1.241791	-2.400585	5.512797
C	0.818379	-1.576817	6.554749
F	1.210306	0.221566	8.034669
H	0.639111	-3.264894	5.244236
F	-0.375862	-1.766814	7.120725
Cu	1.131562	-1.279030	3.473121
H	2.428696	1.901433	3.262061
F	1.724191	2.407986	0.798551
C	1.458933	1.513654	2.967849
C	0.985578	1.741811	1.691020
C	0.647352	0.811267	3.873878
C	-0.273925	1.292237	1.288573
H	0.920717	0.801370	4.925927
F	-0.709068	1.533625	0.053090
C	-0.611880	0.313916	3.478687
C	-1.052237	0.570481	2.182279
H	-1.285990	-0.160835	4.186961
F	-2.244942	0.128432	1.775020

$[Cu(3FB)_2]^+_{(3FB)}$

Cu	8.729052	0.060180	10.111277
F	7.679307	0.544715	13.610971
F	6.379741	2.783693	12.828050
F	7.348049	4.285036	10.820285
C	9.871250	1.679308	10.925829
H	10.853304	1.463403	10.511871
C	9.361219	0.870350	11.973130
H	9.939817	0.053638	12.400200
C	8.172354	1.265768	12.603384
C	7.504798	2.416355	12.218093

C	8.022950	3.187908	11.173455
C	9.185330	2.835852	10.518040
H	9.564070	3.464118	9.718860
F	10.629903	-0.368215	6.885644
F	11.650512	-2.546164	8.113702
F	10.175649	-3.918500	9.899299
C	7.780719	-1.365025	8.925880
H	6.725052	-1.132413	9.045697
C	8.564729	-0.615302	8.015681
H	8.142886	0.202724	7.438531
C	9.871674	-1.036112	7.755731
C	10.405838	-2.156900	8.377216
C	9.628065	-2.859438	9.298701
C	8.331353	-2.475626	9.594407
H	7.746840	-3.055093	10.301206

3FB$_{(3FB)}$

F	1.317780	-0.178778	2.333471
C	0.659685	-0.089356	1.168163
C	-0.727739	-0.031331	1.141643
F	-1.386270	-0.067389	2.315187
C	-1.421766	0.060731	-0.052268
H	-2.505856	0.104098	-0.034563
C	-0.702693	0.095087	-1.244390
H	-1.234996	0.166996	-2.186935
C	0.688864	0.038564	-1.245855
H	1.263126	0.064338	-2.166169
C	1.352741	-0.053077	-0.034948
F	2.697125	-0.109852	0.006693

$[Ag(1FB)_3]^+_{(1FB)}$

H	3.709484	-1.355462	0.826255
C	3.033435	-2.132971	1.168523
C	3.544872	-3.347368	1.609730
H	4.612712	-3.540454	1.633084
C	2.653962	-4.328198	2.026870
F	3.151217	-5.504575	2.457954
C	1.280648	-4.150810	2.029331
H	0.625685	-4.949077	2.363012
C	0.771952	-2.930946	1.573063
H	-0.303503	-2.791176	1.499777
C	1.646429	-1.914439	1.137739
H	1.248158	-1.008459	0.686852
H	4.195974	-0.514797	5.157664
C	3.280995	-0.918928	5.580220
H	2.993793	0.721362	6.966229
H	3.371005	-2.747970	4.425336
C	2.623459	-0.227377	6.590954
C	2.785529	-2.152482	5.122740
C	1.470307	-0.783767	7.128373
C	1.605219	-2.678375	5.684042
C	0.936119	-1.986292	6.694428
F	0.835172	-0.116261	8.112339
H	1.238796	-3.649869	5.363580
H	0.029523	-2.375883	7.145953
Ag	1.271184	-1.284606	3.456816
H	1.853304	1.838777	4.114901
H	1.777173	2.512738	1.708100
C	1.045749	1.493644	3.475853
C	1.005147	1.884661	2.140739
C	0.026129	0.684470	4.012131
C	-0.065300	1.463380	1.364570
H	-0.009276	0.489580	5.082088
F	-0.112595	1.850915	0.074371
C	-1.030498	0.260579	3.184321
C	-1.081031	0.653450	1.848781
H	-1.830732	-0.345960	3.598253
H	-1.891966	0.347355	1.195797

$[Ag(2FB)_2]^+_{(2FB)}$

F	2.208725	-4.538436	0.524112
C	1.288717	-3.607282	0.802308
C	-0.057250	-3.985292	0.825886
F	-0.366909	-5.261591	0.569088
C	-1.048170	-3.070334	1.114251
H	-2.086812	-3.385589	1.119656
C	-0.688072	-1.735092	1.368179
H	-1.465201	-0.983246	1.481446
C	0.673093	-1.352748	1.344292
H	0.948721	-0.305070	1.436650
C	1.667267	-2.307709	1.066716
H	2.717789	-2.036377	1.035583
F	0.584204	1.669298	6.621146
C	0.275995	0.392652	6.364345
F	-1.990574	0.943144	6.666596
H	2.306186	-0.204581	6.069946
C	-1.069524	0.013075	6.388191
C	1.267934	-0.521108	6.075666
C	-1.446588	-1.286948	6.123878
C	0.909382	-1.856722	5.821760
C	-0.451304	-2.240747	5.846049
H	-2.496769	-1.559566	6.155328
H	1.687366	-2.607579	5.707922
H	-0.725680	-3.288798	5.754071
Ag	0.108895	-1.796736	3.595114

$[Ag(3FB)_2]^+_{(3FB)}$

Ag	9.021958	0.355299	9.856163

F	7.943594	0.469560	13.643002
F	6.273669	2.501133	13.018401
F	6.951996	4.290376	11.130665
C	9.889913	2.163314	11.071088
H	10.892867	2.150626	10.650778
C	9.534207	1.207511	12.051729
H	10.237810	0.452815	12.393934
C	8.303525	1.340993	12.698747
C	7.442742	2.383837	12.392890
C	7.809688	3.305204	11.410268
C	9.013767	3.209821	10.740607
H	9.275330	3.951359	9.993200
F	10.827483	-0.953912	6.680803
F	11.517275	-3.170115	8.067125
F	9.841402	-4.208239	9.894403
C	7.865373	-1.388220	8.757456
H	6.857786	-0.991561	8.853933
C	8.747215	-0.823249	7.805705
H	8.439279	-0.009156	7.153872
C	9.981033	-1.444227	7.587984
C	10.341082	-2.586449	8.285831
C	9.458754	-3.114391	9.230378
C	8.232105	-2.532112	9.482619
H	7.564597	-2.975949	10.213560

[(4FB)Ag{Al(OR^F)$_4$}]$_{(gas)}$

Ag	-2.115986	11.065820	23.634948
Al	-3.704134	13.054347	21.832408
O	-4.134660	12.555334	23.505452
C	-4.584558	12.990450	24.723888
C	-3.437335	13.759321	25.466147
F	-2.476275	12.875681	25.864906
F	-3.857142	14.417612	26.550182
F	-2.851156	14.623520	24.635816
C	-5.829781	13.938590	24.582811
F	-6.656049	13.460832	23.647864
F	-5.438206	15.167084	24.220911
F	-6.510304	14.041593	25.738127
C	-5.027606	11.745029	25.580089
F	-6.196343	11.259770	25.155278
F	-5.126913	12.033683	26.883972
F	-4.119735	10.736717	25.469606
O	-4.121495	14.651824	21.340256
C	-4.910665	15.463943	20.578364
C	-4.553090	15.327136	19.053716
F	-4.985010	14.137931	18.592777
F	-5.111386	16.298343	18.311280
F	-3.230740	15.379456	18.861923
C	-4.667199	16.951190	21.029411
F	-4.621087	17.038655	22.364000
F	-3.496284	17.409683	20.553845
F	-5.642033	17.766324	20.585569
C	-6.435834	15.110493	20.762046
F	-6.923206	15.643692	21.895102
F	-7.196998	15.547330	19.747496
F	-6.579245	13.775196	20.842370
O	-4.241142	11.794453	20.776268
C	-4.673666	10.541792	20.520409
C	-5.524241	9.980407	21.713872
F	-4.700961	9.635106	22.746008
F	-6.235121	8.892749	21.390340
F	-6.361255	10.913614	22.171112
C	-5.566043	10.571942	19.222111
F	-4.989588	11.320211	18.278682
F	-6.769105	11.102532	19.497285
F	-5.757900	9.335776	18.721126
C	-3.448465	9.589482	20.264058
F	-2.898282	9.821226	19.068480
F	-3.769755	8.290498	20.341951
F	-2.479050	9.820242	21.193637
O	-1.917082	12.842009	22.055833
C	-0.743053	13.220298	21.441902
C	-0.515780	14.772154	21.609063
F	-1.259811	15.450018	20.728134
F	0.771464	15.115341	21.426506
F	-0.876188	15.154542	22.840279
C	0.442109	12.467567	22.134837
F	0.174856	11.135891	22.222648
F	0.606809	12.904114	23.397421
F	1.593675	12.595770	21.477347
C	-0.752761	12.848219	19.913803
F	-0.489262	11.541785	19.733126
F	0.156094	13.551409	19.222176
F	-1.965550	13.089589	19.401218
F	-1.717911	7.272102	22.762603
F	0.962790	7.589885	22.527883
F	2.285885	9.279582	24.171281
F	0.949180	10.673739	26.067752
C	-1.106152	9.721042	25.391105
H	-1.607454	10.196214	26.229199
C	-1.800144	8.840955	24.528052
H	-2.849884	8.609573	24.684161
C	-1.086789	8.122902	23.569024
C	0.291027	8.263404	23.451833
C	0.974334	9.130937	24.303311
C	0.274880	9.852931	25.262426

[Ag{Al(OR^F)$_4$}]$_{(gas)}$

F	-4.003229	1.775315	12.809002
F	-3.642581	-0.230871	11.133283
F	-1.524363	3.156810	12.840211
F	-5.986967	-1.612155	10.052403
F	-4.105406	3.790674	13.616439
F	-3.247908	-2.197679	10.301046
F	-5.963383	0.559477	9.981688
F	-1.748690	1.800433	11.155577
C	-4.277102	3.056742	12.503099
C	-3.240369	-0.878390	10.033585
C	-1.887774	3.077178	11.549781
C	-5.677873	-0.553762	9.303845
F	-5.569389	3.122100	12.152563
F	-1.988490	-0.509594	9.750957
C	-3.369506	3.553510	11.318072
C	-4.183281	-0.532414	8.824811
F	-1.028719	3.814704	10.825752
F	-6.499796	-0.569072	8.204456
F	-2.584234	5.632055	12.276371
F	-4.598138	-2.786611	8.030599
O	-3.885122	3.063700	10.155055
O	-3.906447	0.692441	8.284238
C	-3.375523	5.126615	11.313078
C	-3.962574	-1.651337	7.731978
F	-6.992193	3.161866	8.843418
F	-4.616977	5.604428	11.509925
F	0.102573	1.264761	8.668523
F	-2.668939	-1.906944	7.553099
Al	-3.580086	2.443949	8.578363
F	-2.937380	5.586242	10.132314
F	-4.446123	-1.247676	6.507819
C	-7.081955	3.628240	7.597049
C	0.334853	2.257583	7.800259
F	-7.554543	2.599394	6.822944
F	0.887572	3.276166	8.476588
O	-4.769839	3.072549	7.375014
O	-1.972905	2.785747	8.069360
F	-5.608383	5.474237	8.996626
F	-1.265167	0.412897	6.456682
F	-7.983742	4.609674	7.559010
F	1.231354	1.820350	6.893925
C	-5.668132	4.058702	7.070759
C	-1.013018	2.708750	7.121259
C	-5.270403	5.440143	7.701719
C	-1.384931	1.670936	6.004154
F	-3.949192	5.614529	7.596223
F	-2.692710	1.829342	5.641850
F	-5.713880	3.029557	4.868558
F	-0.841112	5.076994	7.405541
C	-5.717978	4.246332	5.505672
C	-0.816133	4.125542	6.468370
F	-5.888238	6.462025	7.083418
F	-0.650784	1.795716	4.894273
F	-6.820789	4.882283	5.102602
F	0.343822	4.218610	5.794705
F	-4.645951	4.903146	5.067454
F	-1.820332	4.380374	5.601468
Ag	-5.330694	1.023586	6.311110

[(4FB)Cu{Al(OR^{F})$_4$}]$_{(gas)}$

Cu	-2.003107	11.244368	23.587259
Al	-3.706183	12.954229	21.861972
O	-4.005041	12.474558	23.571421
C	-4.569360	12.917826	24.744882
C	-3.509274	13.758434	25.534741
F	-2.536951	12.937096	26.014531
F	-4.032874	14.421795	26.570084
F	-2.914554	14.631799	24.717078
C	-5.842945	13.806361	24.503361
F	-6.547412	13.309554	23.480768
F	-5.492563	15.063519	24.209859
F	-6.634150	13.841587	25.589185
C	-5.017442	11.674237	25.596294
F	-6.150602	11.150508	25.118822
F	-5.192688	11.978567	26.888783
F	-4.078649	10.699951	25.540142
O	-4.075780	14.576803	21.412292
C	-4.806024	15.445817	20.649722
C	-4.372577	15.382539	19.139071
F	-4.767287	14.210504	18.607281
F	-4.908664	16.380099	18.416112
F	-3.043479	15.463155	19.007916
C	-4.560531	16.906358	21.185234
F	-4.576880	16.932125	22.522217
F	-3.361573	17.368190	20.788738
F	-5.499786	17.757563	20.733622
C	-6.347690	15.117953	20.733056
F	-6.877884	15.590896	21.874112
F	-7.047232	15.639412	19.714567
F	-6.526007	13.786446	20.717220
O	-4.369380	11.759054	20.814822
C	-4.763598	10.498732	20.527593
C	-5.566360	9.878669	21.723776
F	-4.711735	9.541562	22.728188
F	-6.246304	8.775608	21.384550
F	-6.426242	10.770930	22.220377
C	-5.689984	10.545371	19.254445
F	-5.171492	11.360404	18.332172

F	-6.908250	11.009139	19.579757
F	-5.840418	9.324818	18.705037
C	-3.515809	9.597726	20.214276
F	-3.009701	9.880055	19.007737
F	-3.791097	8.286788	20.263090
F	-2.536118	9.842014	21.122240
O	-1.900138	12.631796	22.076761
C	-0.745838	13.031327	21.413100
C	-0.451879	14.543969	21.736581
F	-1.253531	15.339703	21.023153
F	0.822468	14.873354	21.468974
F	-0.680642	14.779111	23.035957
C	0.463096	12.177021	21.913035
F	0.134898	10.865738	21.922032
F	0.789735	12.506371	23.179343
F	1.541421	12.325599	21.143864
C	-0.888836	12.816186	19.859956
F	-0.589577	11.554129	19.513169
F	-0.089576	13.636722	19.167107
F	-2.159499	13.039428	19.496621
F	-1.619923	7.515997	22.732668
F	1.079766	7.726818	22.767943
F	2.291010	9.514263	24.383985
F	0.823194	11.100881	26.017978
C	-1.192833	10.162523	25.211974
H	-1.733295	10.640697	26.023104
C	-1.830652	9.234649	24.343917
H	-2.885022	8.995077	24.445104
C	-1.044027	8.415874	23.527026
C	0.339044	8.496418	23.554083
C	0.967111	9.419515	24.395345
C	0.205405	10.242258	25.207650

$[Cu\{Al(OR^F)_4\}]_{(gas)}$

F	-3.908209	1.773228	13.030980
F	-3.750951	-0.267812	11.048929
F	-1.480189	3.297835	12.950956
F	-6.075879	-1.644723	10.008541
F	-4.088656	3.857075	13.636691
F	-3.268680	-2.144761	10.068269
F	-6.119632	0.531169	10.034786
F	-1.642604	1.715570	11.467409
C	-4.226018	3.008188	12.602505
C	-3.364812	-0.815260	9.891116
C	-1.829528	3.024906	11.682346
C	-5.849589	-0.541381	9.286557
F	-5.516124	2.993069	12.240370
F	-2.154846	-0.337194	9.575831
C	-3.322886	3.419745	11.384823
C	-4.380360	-0.469774	8.745065
F	-0.994146	3.693364	10.866157
F	-6.713841	-0.534679	8.241108
F	-2.638517	5.646887	12.058391
F	-4.754659	-2.688345	7.841518
O	-3.796394	2.777022	10.281386
O	-4.163374	0.789146	8.227833
C	-3.401734	4.973354	11.185365
C	-4.187924	-1.511439	7.582847
F	-6.611114	3.375763	9.090575
F	-4.666516	5.408080	11.316198
F	0.404619	1.341123	8.668428
F	-2.895580	-1.692253	7.308896
Al	-3.533153	2.451076	8.619712
F	-2.988188	5.285698	9.939829
F	-4.766317	-1.037499	6.427241
C	-6.875053	3.635213	7.808363
C	0.464361	2.390600	7.839946
F	-7.310454	2.472005	7.244929
F	0.849639	3.460292	8.553450
O	-4.521929	3.301938	7.392059
O	-1.882735	2.532450	8.152271
F	-5.529475	5.829171	8.753396
F	-0.863510	0.356534	6.468127
F	-7.870153	4.522610	7.733992
F	1.409414	2.141499	6.912981
C	-5.571952	4.115253	7.077076
C	-0.944754	2.648004	7.185062
C	-5.281085	5.617735	7.454986
C	-1.173579	1.589973	6.049060
F	-4.002261	5.910184	7.212541
F	-2.495842	1.561469	5.696471
F	-5.716568	2.723560	5.077933
F	-1.147502	5.005312	7.520351
C	-5.786796	4.051369	5.518211
C	-0.969649	4.091135	6.560934
F	-6.055407	6.456788	6.740394
F	-0.478331	1.852825	4.937501
F	-6.974751	4.497124	5.126349
F	0.161543	4.382472	5.898153
F	-4.826684	4.695844	4.868143
F	-1.998125	4.206922	5.691441
Cu	-4.981782	1.284452	6.443157

Computed IR spectrum of $[Cu(C_6H_6)_3]^+$ at the RI-r^2SCAN-3c(D4)/def2-mTZVPP level of theory

Mode	freq	eps	Int	T**2	TX	TY	TZ
	cm**-1	L/(mol*cm)	km/mol	a.u.			
6:	11.24	0.000001	0.00	0.000016	(0.001006	-0.003378	0.001886)
7:	16.47	0.000000	0.00	0.000007	(0.000698	-0.002247	-0.001186)
8:	29.10	0.000000	0.00	0.000004	(-0.001111	-0.000383	-0.001682)
9:	68.10	0.000002	0.01	0.000008	(-0.002514	-0.000925	0.000663)
10:	81.36	0.000097	0.49	0.000373	(0.004835	-0.010101	0.015732)
11:	82.64	0.000101	0.51	0.000381	(-0.002936	0.014290	0.012966)
12:	93.60	0.000067	0.34	0.000223	(0.014813	-0.000346	-0.001726)
13:	98.07	0.000031	0.16	0.000098	(-0.003742	-0.008249	-0.003987)
14:	98.66	0.000027	0.14	0.000086	(0.004475	-0.006508	0.004900)
15:	107.37	0.000017	0.09	0.000051	(-0.001469	0.005402	-0.004413)
16:	107.96	0.000013	0.07	0.000038	(0.000569	-0.001441	-0.005975)
17:	116.30	0.000013	0.06	0.000034	(-0.005327	-0.002301	-0.000592)
18:	221.33	0.000130	0.66	0.000184	(0.000419	0.003748	0.013020)
19:	224.50	0.000315	1.59	0.000438	(0.004815	0.000970	0.020356)
20:	225.99	0.000374	1.89	0.000517	(0.010282	-0.020253	0.001011)
21:	397.78	0.000013	0.07	0.000010	(-0.002796	-0.000461	-0.001466)
22:	398.61	0.000009	0.04	0.000007	(0.002245	-0.000455	-0.001296)
23:	399.51	0.000010	0.05	0.000008	(0.000505	0.001808	-0.002043)
24:	418.54	0.000011	0.05	0.000008	(-0.002348	-0.001037	-0.001113)
25:	423.61	0.000038	0.19	0.000028	(0.002042	-0.002899	0.003926)
26:	426.47	0.000034	0.17	0.000025	(0.001469	-0.003796	-0.002943)
27:	612.10	0.000219	1.11	0.000112	(-0.005219	0.006156	-0.006818)
28:	612.42	0.000100	0.50	0.000051	(0.000226	0.004273	0.005700)
29:	612.74	0.000112	0.56	0.000057	(-0.005502	0.003016	0.004188)
30:	612.93	0.000073	0.37	0.000037	(0.000737	0.004069	0.004452)
31:	613.15	0.000040	0.20	0.000021	(-0.000805	-0.004409	0.000651)
32:	614.32	0.000031	0.16	0.000016	(-0.002343	-0.002674	0.001812)
33:	679.64	0.000108	0.55	0.000050	(-0.000461	-0.005550	-0.004310)
34:	680.13	0.000129	0.65	0.000059	(0.000046	-0.003808	0.006699)
35:	680.68	0.000104	0.53	0.000048	(-0.005568	0.003437	-0.002231)
36:	717.42	0.000522	2.64	0.000227	(0.001397	-0.012458	0.008351)
37:	719.87	0.033948	171.56	0.014716	(0.041275	-0.025175	0.111260)
38:	720.06	0.034262	173.14	0.014849	(0.046392	-0.105080	-0.040676)
39:	871.69	0.000012	0.06	0.000004	(-0.000095	0.001236	0.001668)
40:	872.03	0.000006	0.03	0.000002	(0.000576	-0.001166	0.000611)
41:	877.31	0.000324	1.64	0.000115	(0.009625	0.004341	-0.001957)
42:	890.59	0.001240	6.27	0.000434	(-0.008583	0.018492	0.004336)
43:	891.43	0.001308	6.61	0.000458	(-0.005898	0.001900	-0.020478)
44:	897.47	0.000016	0.08	0.000006	(-0.000073	-0.002217	0.000880)
45:	971.32	0.000604	3.05	0.000194	(-0.005515	0.012678	-0.001725)
46:	972.29	0.000726	3.67	0.000233	(-0.001158	-0.003013	-0.014915)
47:	973.88	0.000135	0.68	0.000043	(0.006203	-0.000008	0.002208)
48:	986.28	0.000225	1.14	0.000071	(-0.001394	-0.000227	-0.008319)
49:	986.48	0.000272	1.37	0.000086	(-0.004021	0.008357	0.000179)

50:	989.04	0.000076	0.38	0.000024	(0.004454 0.001740 -0.001079)
51:	998.73	0.000045	0.23	0.000014	(0.003006 -0.002190 -0.000599)
52:	999.32	0.000182	0.92	0.000057	(0.002714 -0.006961 0.000986)
53:	999.72	0.000200	1.01	0.000062	(0.000981 0.001469 0.007694)
54:	1009.24	0.000013	0.06	0.000004	(0.000543 -0.000320 0.001877)
55:	1009.54	0.000013	0.07	0.000004	(0.000725 -0.001823 -0.000412)
56:	1010.63	0.000000	0.00	0.000000	(0.000009 0.000165 0.000231)
57:	1027.29	0.000026	0.13	0.000008	(-0.001429 0.002270 -0.000744)
58:	1027.38	0.000317	1.60	0.000096	(0.000021 -0.003747 -0.009066)
59:	1027.49	0.000333	1.68	0.000101	(0.005618 -0.007529 0.003606)
60:	1050.36	0.000579	2.93	0.000172	(0.009268 -0.009285 -0.000087)
61:	1050.54	0.000893	4.52	0.000265	(-0.001183 -0.004163 -0.015706)
62:	1050.64	0.000479	2.42	0.000142	(-0.000965 0.011358 -0.003528)
63:	1057.63	0.000069	0.35	0.000020	(-0.001400 -0.002695 -0.003340)
64:	1058.04	0.000407	2.06	0.000120	(-0.010576 -0.002867 -0.000139)
65:	1058.85	0.000801	4.05	0.000236	(-0.012503 -0.007534 0.004791)
66:	1182.07	0.000713	3.60	0.000188	(0.011149 0.006969 -0.003923)
67:	1184.00	0.000195	0.99	0.000052	(-0.003878 -0.001119 -0.005934)
68:	1184.52	0.000165	0.83	0.000043	(-0.001198 0.006038 0.002344)
69:	1203.75	0.000211	1.06	0.000055	(-0.006147 -0.003864 0.001373)
70:	1204.37	0.000027	0.14	0.000007	(-0.002184 -0.001249 0.000873)
71:	1204.69	0.000019	0.09	0.000005	(0.002160 0.000323 0.000263)
72:	1205.85	0.000039	0.20	0.000010	(-0.002932 -0.001110 0.000409)
73:	1206.64	0.000023	0.11	0.000006	(-0.000478 0.002246 0.000759)
74:	1206.95	0.000018	0.09	0.000005	(-0.000079 -0.000816 0.002017)
75:	1364.32	0.000455	2.30	0.000104	(-0.004083 0.007396 -0.005724)
76:	1364.96	0.000466	2.35	0.000106	(-0.001014 0.006857 0.007644)
77:	1368.76	0.000416	2.10	0.000095	(-0.008816 -0.003635 0.001977)
78:	1375.18	0.000004	0.02	0.000001	(0.000174 0.000883 0.000217)
79:	1375.25	0.000002	0.01	0.000000	(-0.000247 -0.000208 0.000627)
80:	1375.41	0.000006	0.03	0.000001	(0.001187 0.000133 -0.000067)
81:	1498.19	0.005443	27.50	0.001134	(-0.024697 -0.019146 0.012536)
82:	1498.57	0.003839	19.40	0.000799	(-0.023353 -0.008531 -0.013462)
83:	1498.60	0.002474	12.50	0.000515	(0.014105 -0.017719 -0.001474)
84:	1499.36	0.002371	11.98	0.000493	(-0.008626 0.010618 0.017502)
85:	1499.46	0.001601	8.09	0.000333	(0.000721 -0.010874 0.014643)
86:	1499.87	0.000023	0.11	0.000005	(-0.000845 0.001875 -0.000701)
87:	1601.26	0.000118	0.60	0.000023	(-0.000379 0.001295 0.004605)
88:	1601.49	0.000146	0.74	0.000028	(0.004061 -0.003086 0.001539)
89:	1602.63	0.000476	2.41	0.000093	(-0.007970 -0.005029 0.001968)
90:	1614.77	0.000475	2.40	0.000092	(0.006108 0.006672 0.003144)
91:	1615.19	0.000372	1.88	0.000072	(0.007761 -0.003204 -0.001214)
92:	1615.71	0.000400	2.02	0.000077	(-0.003741 -0.004289 0.006698)
93:	3162.31	0.000523	2.64	0.000052	(-0.001091 -0.001589 -0.006919)
94:	3163.97	0.000629	3.18	0.000062	(-0.000449 -0.002396 -0.007489)
95:	3164.37	0.001079	5.45	0.000106	(0.005193 -0.008592 0.002372)
96:	3177.62	0.000006	0.03	0.000001	(-0.000478 -0.000547 0.000284)
97:	3178.06	0.000012	0.06	0.000001	(-0.000447 0.000826 0.000587)
98:	3178.07	0.000013	0.07	0.000001	(0.000439 -0.000828 0.000633)

99:	3183.44	0.000017	0.09	0.000002	(0.000127	0.000212	-0.001280)
100:	3184.42	0.000016	0.08	0.000002	(0.001023	-0.000660	0.000207)
101:	3184.55	0.000022	0.11	0.000002	(-0.000231	0.001280	0.000670)
102:	3191.68	0.000033	0.17	0.000003	(-0.000805	0.001595	-0.000073)
103:	3192.22	0.000030	0.15	0.000003	(-0.000068	-0.000366	-0.001677)
104:	3192.51	0.000008	0.04	0.000001	(-0.000661	0.000409	-0.000428)
105:	3199.13	0.000058	0.29	0.000006	(-0.002345	-0.000243	-0.000366)
106:	3199.37	0.000084	0.42	0.000008	(0.002240	0.000326	-0.001745)
107:	3199.56	0.000190	0.96	0.000018	(0.003211	0.002828	-0.000432)
108:	3206.39	0.000037	0.19	0.000004	(-0.000598	-0.000426	-0.001749)
109:	3206.71	0.000030	0.15	0.000003	(0.000356	-0.001215	0.001141)
110:	3206.78	0.000041	0.21	0.000004	(-0.001101	0.001487	0.000749)

Computed IR spectrum of $[Cu(1FB)_3]^+$ at the RI-r^{2}SCAN-3c(D4)/def2-mTZVPP level of theory

Mode	freq	eps	Int	T**2	TX	TY	TZ
	cm**-1	L/(mol*cm)	km/mol	a.u.			
6:	6.86	0.000008	0.04	0.000361	(0.016208	0.004836	0.008655)
7:	14.11	0.000025	0.13	0.000554	(-0.019194	-0.006467	0.012005)
8:	19.86	0.000264	1.34	0.004152	(-0.053110	-0.034818	0.010896)
9:	54.33	0.000271	1.37	0.001554	(-0.019728	0.034112	-0.001043)
10:	54.49	0.000278	1.40	0.001591	(0.005036	0.004645	0.039296)
11:	63.40	0.000025	0.12	0.000121	(-0.010977	0.000397	-0.000454)
12:	76.99	0.000004	0.02	0.000018	(0.004199	0.000182	-0.000133)
13:	77.70	0.000049	0.25	0.000198	(-0.001041	-0.004788	0.013203)
14:	80.09	0.000074	0.37	0.000286	(-0.003955	0.014283	0.008172)
15:	88.55	0.000084	0.42	0.000296	(-0.017038	-0.000405	-0.002412)
16:	100.10	0.000019	0.09	0.000058	(-0.002939	0.000173	-0.007038)
17:	101.30	0.000031	0.16	0.000095	(0.000725	-0.008283	0.005092)
18:	171.45	0.000011	0.05	0.000020	(0.002892	0.003188	0.000989)
19:	175.87	0.000060	0.30	0.000107	(-0.004540	0.005525	-0.007444)
20:	179.12	0.000060	0.30	0.000105	(0.002825	-0.005435	-0.008188)
21:	295.27	0.000121	0.61	0.000128	(-0.000671	-0.004653	-0.010297)
22:	299.05	0.000100	0.51	0.000104	(0.001965	-0.003996	0.009200)
23:	300.22	0.000124	0.63	0.000129	(0.005292	-0.010048	0.000321)
24:	408.78	0.000069	0.35	0.000053	(0.004169	0.005099	-0.003093)
25:	409.06	0.000055	0.28	0.000042	(0.004361	0.004340	0.002083)
26:	409.99	0.000145	0.73	0.000111	(0.010365	0.001498	-0.000988)
27:	414.06	0.001258	6.36	0.000948	(-0.027460	-0.013486	0.003543)
28:	414.62	0.000005	0.03	0.000004	(-0.000303	-0.000683	-0.001832)
29:	414.80	0.000046	0.23	0.000034	(0.005462	0.002042	0.000646)
30:	504.90	0.000070	0.36	0.000044	(0.004605	0.002924	-0.003716)
31:	507.17	0.002934	14.83	0.001805	(-0.001016	0.016797	0.039017)
32:	507.37	0.002993	15.12	0.001841	(0.019901	-0.034056	0.016876)
33:	521.80	0.001949	9.85	0.001165	(-0.013210	0.029767	0.010234)
34:	521.91	0.001782	9.01	0.001066	(0.007371	-0.007551	0.030890)
35:	524.77	0.000037	0.19	0.000022	(0.000513	0.002545	-0.003922)
36:	617.45	0.000008	0.04	0.000004	(0.000841	0.001803	-0.000498)

37: 617.58 0.000005 0.03 0.000003 (0.000981 0.000218 0.001299)
38: 617.82 0.000154 0.78 0.000078 (-0.007883 -0.003879 0.000827)
39: 660.86 0.000024 0.12 0.000011 (0.001232 0.000858 -0.003019)
40: 662.71 0.001923 9.72 0.000906 (-0.001706 -0.004857 -0.029648)
41: 663.26 0.001981 10.01 0.000932 (0.014754 -0.026585 0.002722)
42: 801.03 0.027782 140.40 0.010823 (-0.003918 0.039440 0.096188)
43: 801.56 0.024363 123.12 0.009485 (0.044272 -0.077275 0.039416)
44: 803.43 0.004559 23.04 0.001771 (-0.022596 0.035335 -0.003402)
45: 826.95 0.004963 25.08 0.001873 (-0.017396 0.036797 -0.014706)
46: 827.09 0.004897 24.75 0.001848 (-0.005647 0.014644 0.040017)
47: 827.88 0.000337 1.70 0.000127 (0.008066 0.002656 0.007397)
48: 842.09 0.000128 0.65 0.000047 (0.001445 -0.000910 0.006674)
49: 842.36 0.000149 0.75 0.000055 (0.003894 -0.006081 -0.001778)
50: 846.12 0.000279 1.41 0.000103 (-0.008666 -0.005263 0.000259)
51: 905.79 0.000406 2.05 0.000140 (-0.000179 -0.008859 -0.007832)
52: 906.32 0.002979 15.05 0.001026 (-0.014739 0.023701 -0.015703)
53: 906.96 0.002819 14.24 0.000970 (-0.004505 0.014019 0.027440)
54: 958.27 0.000648 3.27 0.000211 (0.005639 -0.013251 -0.001882)
55: 959.19 0.000808 4.08 0.000263 (0.002921 -0.001130 0.015908)
56: 963.97 0.000085 0.43 0.000027 (-0.005199 -0.000502 -0.000290)
57: 975.67 0.000437 2.21 0.000140 (0.000896 0.001435 0.011705)
58: 976.45 0.000434 2.19 0.000139 (0.004702 -0.010721 0.001274)
59: 978.76 0.000027 0.14 0.000009 (-0.002879 0.000504 0.000459)
60: 1010.85 0.000558 2.82 0.000172 (-0.005225 0.011497 -0.003574)
61: 1011.63 0.000428 2.16 0.000132 (-0.003098 0.007412 0.008216)
62: 1012.03 0.000417 2.11 0.000128 (-0.001114 0.001128 -0.011224)
63: 1027.12 0.000075 0.38 0.000023 (-0.001447 0.004542 0.000455)
64: 1027.71 0.000552 2.79 0.000168 (0.005760 -0.011568 0.000741)
65: 1027.89 0.000621 3.14 0.000189 (0.001462 0.001035 0.013614)
66: 1087.02 0.000016 0.08 0.000005 (0.000492 -0.000359 0.002080)
67: 1087.22 0.000617 3.12 0.000177 (0.010812 0.007757 0.000042)
68: 1087.61 0.001125 5.69 0.000323 (0.016873 0.005355 -0.003078)
69: 1175.22 0.001885 9.53 0.000501 (-0.018715 -0.011330 0.004692)
70: 1177.23 0.001753 8.86 0.000465 (-0.004230 0.017219 0.012261)
71: 1177.77 0.001458 7.37 0.000386 (0.007945 -0.009567 0.015221)
72: 1183.48 0.001082 5.47 0.000285 (-0.000150 -0.012981 -0.010810)
73: 1183.93 0.003497 17.67 0.000922 (-0.002712 0.003130 -0.030075)
74: 1184.69 0.003023 15.28 0.000796 (-0.014166 0.024382 -0.001112)
75: 1272.91 0.035680 180.31 0.008747 (0.016894 -0.054316 -0.074240)
76: 1273.07 0.036181 182.85 0.008869 (-0.036099 0.065348 -0.057407)
77: 1274.40 0.000440 2.22 0.000108 (0.008129 0.005212 0.003794)
78: 1326.89 0.000092 0.46 0.000022 (-0.004407 -0.000705 0.001278)
79: 1327.05 0.000080 0.40 0.000019 (0.003160 0.002654 -0.001339)
80: 1327.14 0.000558 2.82 0.000131 (-0.009986 -0.005538 0.000848)
81: 1375.03 0.000596 3.01 0.000135 (-0.004663 0.008737 -0.006097)
82: 1375.29 0.000536 2.71 0.000122 (-0.001521 0.006322 0.008910)
83: 1380.42 0.000095 0.48 0.000021 (-0.003897 -0.002338 0.000845)
84: 1469.62 0.002625 13.26 0.000557 (0.021133 0.010134 -0.002830)
85: 1471.75 0.000115 0.58 0.000024 (0.002324 -0.004354 -0.000301)

```
 86:   1472.10   0.000072    0.37  0.000015  (-0.000648 -0.000484 -0.003835)
 87:   1511.92   0.028597  144.52  0.005903  (-0.031494  0.069199 -0.011054)
 88:   1512.18   0.029135  147.24  0.006013  ( 0.002398  0.012455  0.076496)
 89:   1513.50   0.000579    2.93  0.000119  (-0.010642  0.001321  0.002091)
 90:   1601.04   0.000860    4.35  0.000168  (-0.002375  0.010306 -0.007468)
 91:   1601.56   0.000643    3.25  0.000125  ( 0.000446  0.006645  0.008998)
 92:   1603.91   0.002299   11.62  0.000447  (-0.019101 -0.008484  0.003247)
 93:   1619.58   0.025451  128.62  0.004904  ( 0.020645 -0.052924 -0.040950)
 94:   1619.84   0.024244  122.52  0.004671  ( 0.020104 -0.035939  0.054542)
 95:   1620.53   0.002194   11.09  0.000423  (-0.009165  0.000234 -0.018399)
 96:   3170.18   0.000351    1.77  0.000035  ( 0.000654  0.000553  0.005812)
 97:   3171.44   0.000569    2.88  0.000056  (-0.001485  0.004729  0.005610)
 98:   3172.33   0.000764    3.86  0.000075  (-0.004030  0.006320 -0.004352)
 99:   3185.38   0.000008    0.04  0.000001  (-0.000703  0.000309 -0.000439)
100:   3185.93   0.000003    0.02  0.000000  (-0.000371 -0.000292  0.000331)
101:   3186.08   0.000001    0.00  0.000000  (-0.000047 -0.000236  0.000050)
102:   3194.51   0.000087    0.44  0.000009  ( 0.001698  0.000635  0.002284)
103:   3195.41   0.000095    0.48  0.000009  ( 0.000117  0.002976 -0.000669)
104:   3195.88   0.000098    0.49  0.000010  (-0.002587  0.000794  0.001483)
105:   3211.11   0.001072    5.42  0.000104  (-0.009023 -0.004719  0.000712)
106:   3211.15   0.000217    1.10  0.000021  ( 0.004392  0.001337  0.000145)
107:   3211.36   0.000851    4.30  0.000083  (-0.007937 -0.004280  0.001172)
108:   3211.56   0.000101    0.51  0.000010  (-0.001370 -0.002110 -0.001872)
109:   3211.94   0.000083    0.42  0.000008  (-0.000461  0.002506 -0.001241)
110:   3211.99   0.000735    3.71  0.000071  (-0.007813 -0.002563  0.001937)
```

Computed IR spectrum of $[Cu(3FB)_2]^+$ at the RI-r^{2}SCAN-3c(D4)/def2-mTZVPP level of theory

```
Mode   freq       eps      Int      T**2       TX       TY       TZ
       cm**-1  L/(mol*cm) km/mol    a.u.
----------------------------------------------------------------------------
  6:     6.63   0.000408    2.06  0.019202  (-0.016489 -0.116568  0.073092)
  7:    13.67   0.000848    4.29  0.019374  ( 0.042905  0.075151  0.109023)
  8:    25.43   0.000256    1.30  0.003145  (-0.021077 -0.031613 -0.041253)
  9:    43.39   0.000000    0.00  0.000000  ( 0.000084  0.000009  0.000007)
 10:    51.11   0.000048    0.24  0.000292  (-0.017042  0.000428 -0.001342)
 11:    54.35   0.000000    0.00  0.000000  (-0.000260  0.000021  0.000029)
 12:    83.07   0.000967    4.89  0.003632  (-0.005112  0.047190  0.037129)
 13:   137.35   0.000000    0.00  0.000000  ( 0.000003 -0.000002  0.000000)
 14:   145.85   0.000168    0.85  0.000359  ( 0.004905  0.013598  0.012244)
 15:   207.53   0.000000    0.00  0.000000  ( 0.000010  0.000012  0.000010)
 16:   238.77   0.000129    0.65  0.000168  (-0.007365  0.009531  0.004809)
 17:   281.60   0.000028    0.14  0.000030  (-0.000446 -0.005201  0.001796)
 18:   281.81   0.000000    0.00  0.000000  (-0.000001 -0.000049  0.000016)
 19:   288.29   0.000000    0.00  0.000000  (-0.000033  0.000002 -0.000017)
 20:   304.18   0.000207    1.05  0.000213  (-0.012333  0.007133  0.003139)
 21:   308.76   0.000000    0.00  0.000000  ( 0.000032  0.000000 -0.000003)
 22:   309.27   0.000504    2.55  0.000508  ( 0.021804 -0.002276 -0.005279)
 23:   343.62   0.000000    0.00  0.000000  ( 0.000023  0.000048  0.000046)
```

24:	363.50	0.001304	6.59	0.001119	(-0.010151 -0.021152 -0.023850)
25:	478.28	0.000032	0.16	0.000021	(-0.002308 0.002132 0.003340)
26:	479.67	0.000000	0.00	0.000000	(0.000006 0.000001 -0.000002)
27:	500.57	0.000577	2.92	0.000360	(-0.000017 -0.015119 0.011464)
28:	501.50	0.000000	0.00	0.000000	(-0.000002 -0.000017 -0.000009)
29:	558.09	0.000453	2.29	0.000253	(-0.006230 -0.014640 0.000460)
30:	561.25	0.000000	0.00	0.000000	(0.000042 0.000066 0.000013)
31:	578.88	0.000792	4.00	0.000427	(0.001630 0.013030 -0.015947)
32:	579.06	0.000000	0.00	0.000000	(0.000022 0.000135 -0.000155)
33:	587.39	0.000432	2.19	0.000230	(-0.002307 -0.012492 0.008267)
34:	591.65	0.000000	0.00	0.000000	(0.000004 -0.000000 0.000013)
35:	698.65	0.000002	0.01	0.000001	(-0.000655 0.000426 0.000498)
36:	699.19	0.004751	24.01	0.002120	(-0.034378 0.020098 0.023119)
37:	705.19	0.000006	0.03	0.000003	(-0.001620 0.000151 0.000191)
38:	705.60	0.001872	9.46	0.000828	(-0.028634 0.001714 0.002251)
39:	826.24	0.019896	100.54	0.007514	(-0.044423 -0.050127 -0.055029)
40:	830.63	0.000000	0.00	0.000000	(-0.000153 -0.000214 -0.000220)
41:	840.55	0.005922	29.93	0.002199	(-0.014596 0.027161 0.035324)
42:	841.18	0.000001	0.00	0.000000	(0.000175 -0.000351 -0.000436)
43:	891.59	0.000000	0.00	0.000000	(0.000002 0.000008 0.000002)
44:	897.80	0.000322	1.63	0.000112	(0.008210 -0.006358 0.002029)
45:	963.37	0.000000	0.00	0.000000	(0.000078 0.000215 0.000014)
46:	964.97	0.001411	7.13	0.000456	(-0.009208 -0.019227 -0.001309)
47:	1049.92	0.000000	0.00	0.000000	(0.000009 -0.000106 0.000104)
48:	1050.98	0.031989	161.66	0.009498	(-0.010833 0.080031 -0.054555)
49:	1056.50	0.007256	36.67	0.002143	(0.027769 0.010514 -0.035518)
50:	1056.77	0.000000	0.00	0.000000	(-0.000126 -0.000111 0.000209)
51:	1177.33	0.001148	5.80	0.000304	(-0.001369 0.012940 -0.011617)
52:	1178.46	0.000000	0.00	0.000000	(0.000012 -0.000097 0.000090)
53:	1265.98	0.000002	0.01	0.000000	(-0.000196 -0.000414 0.000536)
54:	1266.06	0.019452	98.30	0.004794	(0.017205 0.042771 -0.051664)
55:	1280.40	0.017334	87.60	0.004225	(-0.053298 0.033730 0.015694)
56:	1280.65	0.000002	0.01	0.000000	(-0.000546 0.000358 0.000171)
57:	1327.58	0.031811	160.76	0.007478	(0.055244 -0.055345 -0.036915)
58:	1332.66	0.000000	0.00	0.000000	(0.000050 -0.000083 -0.000041)
59:	1360.24	0.003358	16.97	0.000770	(-0.002923 0.018305 0.020661)
60:	1368.86	0.000000	0.00	0.000000	(0.000005 -0.000053 -0.000062)
61:	1481.46	0.000006	0.03	0.000001	(-0.000018 -0.000864 0.000676)
62:	1482.18	0.034530	174.50	0.007270	(-0.003105 -0.066707 0.053015)
63:	1535.47	0.138754	701.20	0.028200	(-0.131286 0.081296 0.065992)
64:	1541.56	0.000000	0.00	0.000000	(0.000102 -0.000138 -0.000086)
65:	1590.34	0.028994	146.52	0.005689	(0.015293 -0.059255 0.044094)
66:	1592.64	0.000001	0.01	0.000000	(0.000105 -0.000361 0.000275)
67:	1614.60	0.025771	130.23	0.004981	(0.019202 0.042644 -0.052855)
68:	1614.91	0.000311	1.57	0.000060	(-0.002098 -0.004696 0.005803)
69:	3189.68	0.000624	3.15	0.000061	(-0.003834 -0.004881 0.004746)
70:	3189.77	0.000046	0.23	0.000004	(-0.001085 -0.001301 0.001266)
71:	3199.86	0.004466	22.57	0.000436	(-0.014548 0.011865 -0.009115)
72:	3199.99	0.000913	4.61	0.000089	(-0.006566 0.005373 -0.004131)

73: 3216.88 0.004325 21.86 0.000420 (0.014871 0.008803 -0.010995)
74: 3216.94 0.000604 3.05 0.000059 (0.005559 0.003290 -0.004107)

7. References

[1] T. A. Engesser, C. Friedmann, A. Martens, D. Kratzert, P. J. Malinowski, I. Krossing, *Chem. Eur. J.* **2016**, *22*, 15085.

[2] J. Willrett, M. Schmitt, V. Zhuravlev, M. Sellin, P. J. Malinowski, I. Krossing, *Angew. Chem. Int. Ed.* **2024**, *63*, e202405330.

[3] R. Schneider, T. A. Engesser, C. Näther, I. Krossing, F. Tuczek, *Angew. Chem. Int. Ed.* **2022**, *61*, e202202562.

[4] I. Raabe, K. Wagner, K. Guttsche, M. Wang, M. Grätzel, G. Santiso-Quiñones, I. Krossing, *Chem. Eur. J.* **2009**, *15*, 1966.

[5] J. Cosier, A. M. Glazer, *J. Appl. Crystallogr.* **1986**, *19*, 105.

[6] a) Bruker, *SAINT, V 8.40B*. Bruker AXS Inc., Madison, Wisconsin, USA; b) Bruker, *TWINABS*. Bruker AXS Inc., Madison, Wisconsin, USA.

[7] G. M. Sheldrick, *Acta Crystallogr., Sect. A: Found. Crystallogr.* **2015**, *71*, 3.

[8] G. M. Sheldrick, *Acta Crystallogr., Sect. C: Cryst. Struct. Commun.* **2015**, *71*, 3.

[9] C. B. Hübschle, G. M. Sheldrick, B. Dittrich, *J. Appl. Crystallogr.* **2011**, *44*, 1281.

[10] a) D. Kratzert, J. J. Holstein, I. Krossing, *J. Appl. Crystallogr.* **2015**, *48*, 933; b) D. Kratzert, I. Krossing, *J. Appl. Crystallogr.* **2018**, *51*, 928.

[11] D. Kratzert, *FinalCif, V73*. https://www.xs3.uni-freiburg.de/research/finalcif.

[12] C. R. Groom, I. J. Bruno, M. P. Lightfoot, S. C. Ward, *Acta Crystallogr., Sect. B: Struct. Sci* **2016**, *72*, 171.

[13] F. Neese, F. Wennmohs, U. Becker, C. Riplinger, *J. Chem. Phys.* **2020**, *152*, 224108.

[14] S. Grimme, A. Hansen, S. Ehlert, J.-M. Mewes, *J. Chem. Phys.* **2021**, *154*, 64103.

[15] a) F. Weigend, R. Ahlrichs, *Phys. Chem. Chem. Phys.* **2005**, *7*, 3297; b) F. Weigend, *Phys. Chem. Chem. Phys.* **2006**, *8*, 1057.

[16] a) M. Sierka, A. Hogekamp, R. Ahlrichs, *J. Chem. Phys.* **2003**, *118*, 9136; b) R. Ahlrichs, *Phys. Chem. Chem. Phys.* **2004**, *6*, 5119.

[17] E. Caldeweyher, C. Bannwarth, S. Grimme, *J. Chem. Phys.* **2017**, *147*, 34112.

[18] V. Barone, M. Cossi, *J. Phys. Chem. A* **1998**, *102*, 1995.

[19] C. Armbruster, M. Sellin, M. Seiler, T. Würz, F. Oesten, M. Schmucker, T. Sterbak, J. Fischer, V. Radtke, J. Hunger et al., *Nat. Commun.* **2024**, *15*, 6721.

[20] M. Ligare, *Am. J. Phys.* **2010**, *78*, 815.
